# M2 polarization of macrophages: Manipulation of spinal cord injury repair

**DOI:** 10.4103/NRR.NRR-D-24-01579

**Published:** 2025-09-29

**Authors:** Yiran Lu, Hantian Yin, Lingwei Lou, Zhonglin Liu, Haiming Zhu, Chunyi Gu, Can Zhang, Junjuan Wang

**Affiliations:** 1 Hangzhou Medical College, Hangzhou, Zhejiang Province, China; 2 Hunan Research Center of the Basic Discipline for Cell Signaling, College of Biology, Hunan University, Changsha, Hunan Province, China

**Keywords:** activation, biomaterial, clinical trial, inflammatory, macrophage, neuron regeneration, polarization, spinal cord injury, therapeutic strategy, tissue repair

## Abstract

Spinal cord injury results in lasting sensory and motor dysfunction with limited regenerative capacity. Macrophages play a crucial role in orchestrating secondary pathogenesis and repair mechanisms through polarization dynamics. Following spinal cord injury, these immune cells deploy context-dependent responses via divergent regulatory pathways, mediating phagocytic clearance, inflammatory modulation, and neural tissue remodeling. M1 macrophage polarization exacerbates tissue damage through cytokine storms, reactive oxygen species generation, and subsequent neuronal apoptosis, axonal fragmentation, and glial scarring. Conversely, dominant M2 polarization provides neuroprotection by resolving inflammation and promoting axonal sprouting. Strategic manipulation of macrophage plasticity is a promising frontier in spinal cord injury recovery therapy. This review comprehensively examines the regulatory mechanisms that govern macrophage polarization after spinal cord injury, the functional distinctions between resident microglia and peripheral macrophages, the pathophysiological cascades that occur across injury subtypes, and the emerging interventions that span nanotherapeutics, engineered exosomes, electroactive biomaterials, and photobiomodulation. However, there is still a lack of clinical therapies centered around macrophages due to a lack of human trials targeting macrophage reprogramming and excessive reliance on rodent models without validation in non-human primates. However, given the accelerated development of immunomodulatory biomaterials and the expanding mechanistic insights into polarization pathways, the precision targeting of macrophages warrants prioritized investigation for transformative spinal cord injury therapeutics.

## Introduction

Spinal cord injury (SCI) is one of the most severe types of neurological trauma. It is usually caused by direct or indirect external forces, such as vertebral fractures or dislocations. SCI results in significant impairment of motor and sensory abilities below the level of injury, potentially leading to lifelong disabilities (Lu et al., 2024b). SCI not only causes dual physical and psychological harm to patients but also inflicts incalculable damage and losses on both society and families (Jazayeri et al., 2023). Current clinical treatments for SCI are suboptimal. Existing approaches are limited to nonspecific interventions, such as surgical decompression, high-dose steroid pulse therapy, neuroprotective drug administration, and rehabilitative training (Alvi et al., 2024).

Macrophages, consisting of spinal cord-resident microglia and peripherally derived macrophages (PDMs), play a crucial role in SCI treatment (Zhou et al., 2025). These cells exhibit three key functional characteristics. First, they have a large cytoplasm with abundant lysosomes and phagosomes, which gives them the ability to engulf and degrade microorganisms, cellular debris, and apoptotic cells. Second, they serve as antigen-presenting cells (APCs), activating adaptive immunity through major histocompatibility complex (MHC)-mediated antigen presentation to T lymphocytes. Furthermore, macrophages secrete various cytokines and chemokines that modulate inflammatory responses, attract new immune cells, and promote tissue repair and regeneration (Zhao et al., 2023). Macrophages exhibit unique plasticity during immune responses. Upon exposure to specific stimuli, they transiently change from quiescent states to activated forms through macrophage activation. Significantly, these cells demonstrate polarizability: promoting pro-inflammatory responses as M1-polarized macrophages while suppressing inflammation through their M2-polarized counterparts (Zhu et al., 2023c). M1-polarized macrophages primarily mediate the clearance of pathogens and tumor cells, but their hyperactivation can cause tissue damage and hinder repair. Conversely, M2-polarized macrophages primarily facilitate chronic inflammation resolution and promote tissue remodeling, angiogenesis, and immunomodulation. The activation and polarization states of macrophages directly shape the inflammatory microenvironment following SCI. At lesion sites, rapid infiltration of inflammatory cells occurs, particularly macrophages (Wang et al., 2024a). These cells secrete pro-inflammatory mediators that trigger oxidative stress, thereby exacerbating neuroinflammation and accelerating neuronal death and demyelination.

Currently, therapeutic strategies targeting macrophage activation and polarization are under extensive investigation, which mainly focus primarily encompassing pharmacological agents, stem cells, exosomes, and photobiomodulation. These approaches have demonstrated varying degrees of efficacy and even some clinical studies. Their definitive therapeutic outcomes remain uncertain, necessitating further rigorous evaluation. Consequently, elucidating the regulatory mechanisms governing macrophage polarization transitions, identifying pivotal therapeutic targets, and formulating precision-targeted strategies represent critical scientific imperatives to enhance post-SCI repair processes and improve patient quality of life (Wang et al., 2024c).

In this review, we commence by examining the pathological mechanisms and repair challenges of SCI, discussing how macrophage activation and polarization modulate inflammatory responses, and exploring regulatory determinants of macrophage polarization. We focus on solving the central role of macrophages in SCI progression and clinical outcomes, alongside polarization-modulating therapeutic strategies, concluding with a synthesis of current advancements and prospective research.

## Search Strategy

The literature cited in this manuscript was reviewed primarily using the PubMed database with keywords such as “macrophage,” “spinal cord injury,” “inflammatory response,” “polarization,” and “activation,” either individually or in combination. Specifically, the search aimed to elucidate the pathogenic mechanisms of various types of SCI and the specific involvement of macrophages. Preference was given to publications from 2023 to 2025, particularly focusing on articles from high-impact journals. The abstract and conclusion section were the focus for identifying key themes and trends. Additionally, relevant literature cited in or citing these articles was explored to uncover consistencies and discrepancies. The meticulous approach facilitated a comprehensive analysis of the diverse functions of macrophages in SCI and repair, encompassing their regulatory function in inflammation, tissue regeneration, and interactions with various types of cells. Such analysis identified research gaps and trends, informing experimental designs and future studies in SCI repair.

## Macrophage-based Regulation of Pathological Processes in Spinal Cord Injury

### Pathological mechanism of spinal cord injury

SCI is classified through multiple methodologies, according to injury completeness into complete injury. It is marked by the total absence of sensory or motor abilities in the sacral S4–S5 segments or a total loss of all sensory and motor functions below the injury level, and incomplete injury, characterized by the retention of partial sensory or motor function below the lesion (Snider et al., 2023). Etiologically, SCI is classified as either traumatic or non-traumatic. Pathophysiologically, SCI constitutes a complex dynamic process involving primary mechanical insults and secondary inflammatory responses (Tian et al., 2023; Zhang et al., 2024a).

**Figure 1:**
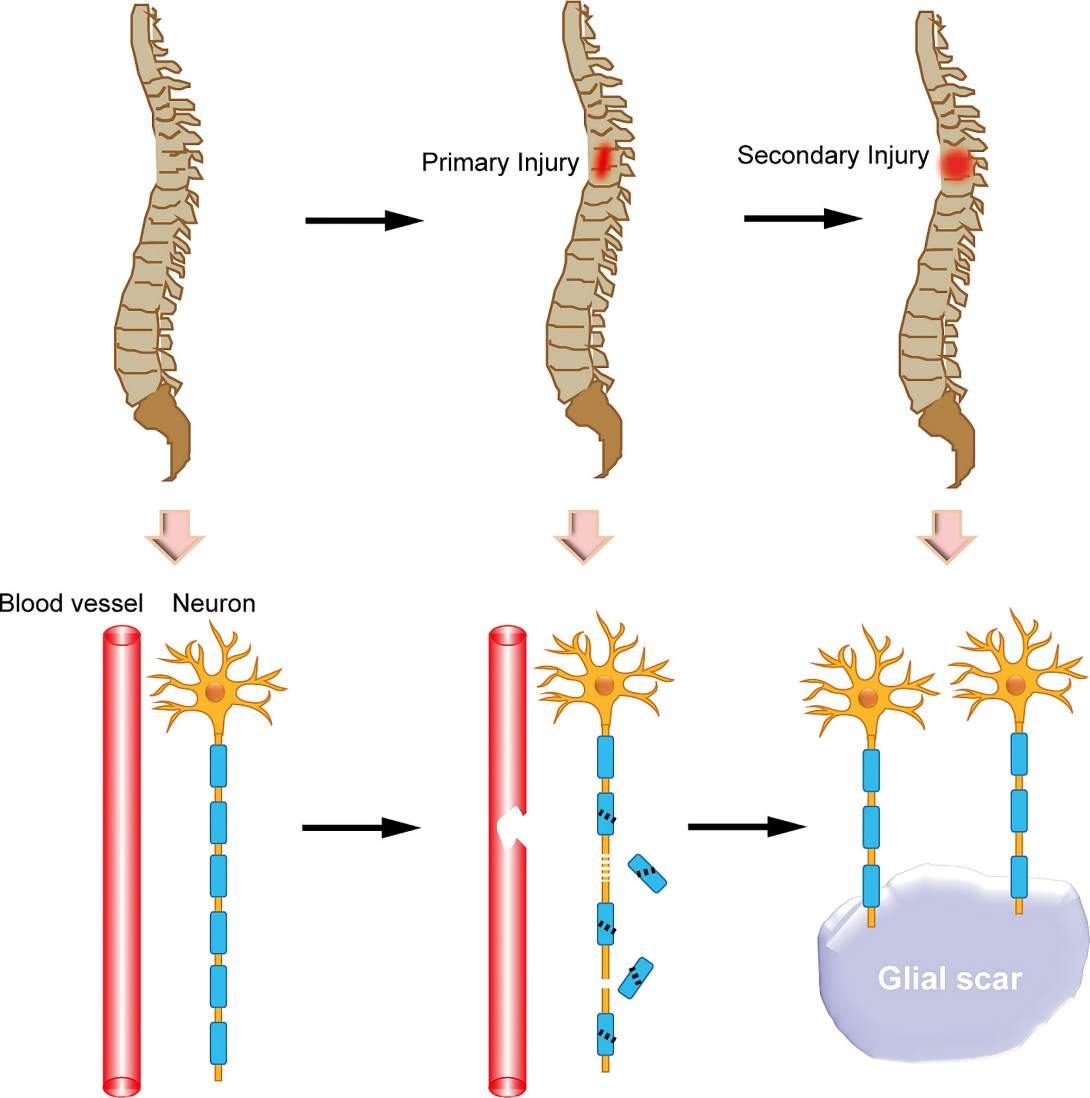
Primary and secondary injuries following spinal cord injury. Primary injury affects the spinal cord by disrupting the myelin sheath, causing bone fragments, and tearing ligaments; secondary injury activates inflammation, develops glial scars, and impedes healing. Reprinted from Li et al. (2024a).

Primary injuries of SCI are mostly caused by sudden spinal cord trauma, such as contusion, compression, dislocation, traction, transection, etc., also known as mechanical injuries; less frequently, non-traumatic causes are involved including tumors and infections. Primary injuries triggered mainly by mechanical forces can result in direct damage to the structure of peripheral neurons and blood vessels, axonal rupture, damage to a large number of nerve fibers, rupture of the blood-spinal cord barrier and even massive hemorrhage within the spinal cord parenchyma, which in turn can lead to acute cellular dysfunction and cellular death (Sterner and Sterner, 2022; Zhou et al., 2023). Primary injury causes secondary injury, which produces further biological and chemical damage to the site of injury at multiple levels: molecular, cellular, tissue, and organ. Secondary injury can trigger an inflammatory cascade response that contributes to the formation of glial scarring while impeding the process of injury repair (Li et al., 2024a; **Figure 1**).

SCI is temporally classified into acute (< 48 hours), subacute (48 hours to 14 days), intermediate (14 days to 6 months), and chronic phases (> 6 months) (Tang et al., 2023a). During the acute secondary injury phase, pathological alterations primarily include vascular damage, excitotoxic effects, ionic imbalance, oxidative stress, axonal demyelination, inflammatory responses, and immune cell activation (Hu et al., 2023b; Shafqat et al., 2023). The ensuing subacute secondary damage phase is marked by neuronal death, Wallerian degeneration, activation of reactive astrocytes, axonal reorganization, and the preliminary creation of glial scars. In the intermediate to chronic phases, cystic cavity expansion, persistent axonal degeneration, and maturation of glial scars dominate. Notably, cellular responses constitute the fundamental units of SCI pathophysiological mechanisms and exhibit distinct spatiotemporal dynamics (Hu et al., 2023b). However, the exact mechanisms governing SCI advancement remain inadequately clarified. Therefore, systematically dissecting temporal and spatial changes in cellular quantity, morphology, and functionality at lesion sites is critical for identifying therapeutic targets and developing interventions.

Chronologically, SCI is categorized into acute (less than 48 hours), subacute (48 hours to 14 days), intermediate (14 days to 6 months), and chronic (more than 6 months) stages (Reyes and Mokalled, 2024). During the acute secondary injury phase, the primary manifestations include vascular damage, excitotoxic effects, ionic balance imbalance, oxidative stress damage, axonal demyelination, inflammatory responses, and immune cell activation (Jin et al., 2023; Han et al., 2024). It is succeeded by the subacute secondary injury phase, distinguished by neuronal apoptosis, Wallerian degeneration, activation of reactive astrocytes, axonal remodeling, and the initiation of neuroglial scarring. In the medium to chronic secondary injury stage, it is characterized by growth of the cystic lumen, axonal death and maturation of the glial scar (Saremi et al., 2022; Shafqat et al., 2023). The cellular response is the basic unit in the pathophysiological mechanism of SCI, possessing distinct temporal and spatial properties (Li et al., 2022a). Nevertheless, the many mechanisms underlying the degenerative phase of SCI remain inadequately comprehended. Therefore, elucidating the mechanisms of the number, morphology, and functional changes of various types of cells at the location of injury is of great significance for the intervention of SCI therapeutic targets.

As soon as the injury occurs, the local vascular endothelial cells in spinal cord are damaged, their permeability increases dramatically, and inflammatory blood cells like neutrophils and monocytes quickly penetrate the area of injury. Among these, neutrophils are the first to enter the spinal cord and become activated following SCI. They secrete many inflammatory mediators, chemokines, and proteolytic enzymes, including interleukin (IL)-1β, tumor necrosis factor-α (TNF-α), IL-6, among others. Positive feedback also encourages the activation of inflammatory cells, including microglial cells and self- and blood-borne macrophages, which sets off a chain of cascade reactions (Ding and Chen, 2023; Lu et al., 2024c). Neutrophils will peak within a day and gradually decrease over a week (Orr and Gensel, 2018; He et al., 2022). Thereafter, the infiltrating core cells turn into macrophages or microglia. In addition, activated microglia contribute to the activation of type A1 reactive astrocytes via insulin-like growth factor 1, which amplify the inflammatory response and produce neurotoxicity, which in turn leads to the demise of neurons and oligodendrocytes and encourages glial scarring (Wang et al., 2021b).

The early inflammatory response helps to remove tissue debris and stimulate neuronal regeneration. As the inflammation advances, many cytokines, ROS and proteases are released and oxidative stress is exacerbated, further exacerbating tissue damage (Li et al., 2022d). Particularly during the medium and chronic stages of secondary SCI, the ongoing inflammatory response hampers the restoration of neurological function (Liu et al., 2024).

In SCI, three primary types of cell death are observed: apoptosis, necrosis, and autophagy (Yin et al., 2022). Among them, apoptosis, as one of the key pathological features of secondary injury, primarily occurs and directly determines the difficulty of motor function recovery in SCI patients (Ding and Chen, 2024). Vascular damage caused by primary injury can result in the blockage of glucose and oxygen transport, impaired mitochondrial function, reduced adenosine triphosphate production, and ablation of ionic gradient to allow Ca^2+^ influx into the cell, which triggers excessive reactive oxygen species (ROS) and reactive nitrogen species production (Wang et al., 2024e). In addition, a large accumulation of free radicals and free radical-induced damage can also trigger a series of oxidative stress responses, such as ROS and reactive nitrogen species production, accumulation of oxidative damage markers, lipid peroxidation, and protein and DNA damage, leading to neuronal iron death and impaired neurological function; at the same time, the reactive substances mentioned above triggered oxidative damage and induced mitochondrial dysfunction, which would, in turn, trigger the production of more reactive substances and exacerbate oxidative damage and ultimately contribute to cell death, leading to pathological progression and preventing neuronal self-repair (Slater et al., 2022). As described by Schmidt and Quintá (2023), the inhibition of ROS production safeguards damaged spinal cord tissues from neuronal apoptosis, mitochondrial oxidative stress, and axonal degeneration. How to reduce ROS production, attenuate mitochondrial oxidative stress and its dysfunction, and minimize cell death is a significant target for intervening in secondary injury in SCI.

Axonal degeneration and demyelination occur during the secondary injury phase of SCI (Du et al., 2022), chronic demyelination plays an important role in persistent axonal loss, but persistent inflammation poses difficulties in promoting re-myelination and repair. At present, there are few treatment methods for reducing inflammatory demyelination through immunomodulatory therapy (Mey et al., 2023). Regional macrophage indicators in diminished myelin sheaths exhibit differential expression of pro- and anti-inflammatory markers relative to the spinal cord in SCI. It increases the possibility of chronic and progressive inflammation in the surrounding spinal cord region leading to demyelination and re-myelination failure (Mahajan et al., 2025). Hematopoietic stem cell-derived phagocytic macrophages transduced by Nogo receptor-Fc are crucial for the delivery of fusion peptides. The phenotype exhibits anti-inflammatory properties and can alter its polarization to expedite the removal of myelin debris, hence facilitating the preservation, regeneration, and re-myelination of axons (Ye et al., 2023). Some scholars have also pointed out the significant role of Schwann cells in self-secreting signals during Waller degeneration, and have identified potential mechanism targets for accelerating myelin clearance and improving demyelinating diseases (Huang et al., 2024). Some scholars have attempted to explore that the natural product icariin can serve as a potential drug for demyelination treatment by regulating Nuclear factor erythroid 2-related factor 2/Heme oxygenase 1 mediated antioxidant stress and toll-like receptor (TLR)4/nuclear factor-κB (NF-κB)-mediated inflammatory responses (Song et al., 2024b). Some scholars have emphasized the core role of the TREM2 signal in re-myelination and neuroprotection. These findings provide insights into how chronic demyelination leads to axonal damage and help identify new neuroprotective therapeutic targets for multiple sclerosis (Wang et al., 2023c). Myelin integrity is critical for the recovery of physiological functions at the site of SCI. Demyelination after SCI is closely associated with massive death of oligodendrocytes (Al Mamun et al., 2024). Demyelination of surviving axons occurs because of continued oligodendrocyte apoptosis, coupled with Wallerian degeneration of the distal stumps of transected axons and proximal axonal death (Shafqat et al., 2023). When SCI occurs, the communication between myelin glial cells and axons is disrupted, and axonal demyelination begins to occur along with the loss of neuronal function. A recent study has shown that clemastine treatment can maintain myelin integrity, reduce axonal loss, and enhance functional recovery (Myatich et al., 2023). The processes of axonal degeneration and demyelination after SCI may be causally related to each other, leading to a malignant progression of both sides.

In the subacute and intermediate chronic phases of SCI, astrocytes undergo proliferation, hypertrophy, and an upregulation of glial fibrillary acidic protein, exhibiting the morphological traits of reactive astrocytes (Tamaru et al., 2023).

They commence migration around the periphery of the damaged tissue, where they, in conjunction with inflammatory cells, will encircle the core of the lesion to establish a neuroglial scar, typically accompanied by a cavity filled with chondroitin sulfate proteoglycan (Tomé et al., 2023). The neuroglial scar, which begins to form in the subacute phase and matures in the mid-chronic phase, is a multifaceted process involving a variety of cell types, including reactive astrocytes, fibroblasts, microglia, macrophages and oligodendrocytes (Patil et al., 2023). Although glial scars are often regarded as a difficult obstacle to axonal regeneration, the data from BM can open up new avenues for advancing the regeneration research of central nervous system (CNS) injuries (including SCI) (Sekiya and Holley, 2025), which has traditionally been regarded as one of the main reasons why glial scarring leads to impaired recovery after SCI (Perez-Gianmarco and Kukley, 2023). It has been suggested that fibroblast growth factor 2 is able to modulate the inhibitory environment that arises after SCI, mitigating the inflammatory response, reducing astrocyte proliferation, and lowering chondroitin sulfate proteoglycan levels to facilitate axonal regeneration (Tomé et al., 2023). As a promising new method, spinal cord fusion has effectively restored morphological continuity and electrical reconnection at both ends of the cross-sectional spinal cord, offering a novel strategy for future SCI repair (Shen et al., 2024b). The expanding process of fluid-filled cysts surrounded by glial scar tissue can further compress residual neural tissue and cause continued deterioration of neurological function.

It is crucial to recognize that among the several mechanisms contributing to secondary SCI, inflammation directly or indirectly governs the progression of SCI and serves as the primary pathophysiological foundation of secondary injury (An et al., 2021). However, some scholars have pointed out that cells rich in serum-free regulated medium may mitigate the biosafety issues linked to animal-derived components, rendering it a viable option for clinical applications in SCI treatment (Subbarayan et al., 2024). Some scholars have also shown that intramural injection of bioactive agents can easily reach the injury site and fill post-traumatic cysts, which indicates that the delivery of bioactive agents by cerebrospinal fluid to the cyst cavity can represent a promising therapeutic strategy (Telegin et al., 2023). With further research, scholars have confirmed that neural communication between cells involved in the inflammatory response and nerve cells is abnormally active during the pathogenesis of SCI (Hu et al., 2023b). Therefore, controlling the inflammatory response following SCI is crucial for facilitating tissue healing and the restoration of neurological function at the damaged site.

### Role of macrophages in spinal cord injury

Macrophages, essential components of the mononuclear phagocyte system, are ubiquitously distributed across tissues and originate from bone marrow hematopoietic stem cells. They exhibit potent phagocytic and degradative capacities, enabling clearance of pathogens, foreign substances, and apoptotic cellular debris. As essential innate immune cells, macrophages commence immune responses by identifying pathogen-associated molecular patterns and damage-associated molecular patterns (DAMPs), while additionally functioning as APCs in adaptive immunity. Furthermore, they critically contribute to tissue repair, inflammation regulation, and maintenance of tissue homeostasis. In the CNS, resident immune cells known as microglia, originate from embryonic neural crest cells, which constitute approximately 20% of total CNS glial cells and are spread across the brain and spinal cord. Their primary functions include maintaining neural equilibrium, conducting immune surveillance, and participating in tissue damage repair (Presta et al., 2018).

Following SCI, investigations into cellular mechanisms at the lesion site have revealed intricate interactions among diverse cell types. Astrocytes, oligodendrocytes, microglia, and macrophages are essential for tissue repair and functional restoration. Research on brain-derived neurotrophic factor expression in astrocytes, microglia/macrophages, and oligodendrocytes indicates that these cells promote neuronal survival and functionality through brain-derived neurotrophic factor release, providing a theoretical foundation for neuroprotection and regeneration post-SCI (Dougherty et al., 2000). The discovery establishes the basis for understanding glial cell involvement in neuroprotection and repair. Emerging research clarifies the evolving roles of microglia and macrophages in post-injury healing processes. Notably, they mitigate secondary damage and promote tissue repair through lesion containment and scar compaction (Zhou et al., 2020b). These findings highlight the plasticity of immune cells, revealing that macrophages and microglia can adopt beneficial phenotypes to facilitate repair in damaged tissues.

In recent years, advances in stem cells, biomaterial scaffolds, and cellular reprogramming strategies following SCI have unveiled cellular regenerative potential, driving progress in cell replacement therapies. In addition, inhibition of the NF-κB/dynamin-related protein 1 axis has been demonstrated to block pro-inflammatory phenotypic polarization, proposing novel neuroprotective approaches for SCI (Song et al., 2024a). The emergence of single-cell RNA sequencing technology has further enabled comprehensive profiling of post-SCI cellular heterogeneity and intercellular crosstalk, delineating how immune response dynamics govern spinal repair (Milich et al., 2021). Additionally, investigations into age-related immune and lymphatic responses reveal that aging exacerbates post-traumatic neuroinflammation and impedes functional recovery, providing critical insights for optimizing therapeutic paradigms in elderly SCI populations (Salvador et al., 2023).

Recently, researchers have also discovered that exosomes generated from Schwann cells, which contain milk fat globule-epidermal growth factor-factor 8 (MFG-E8), modulate macrophage and microglial polarization through the suppressor of cytokine signaling-3/signal transducer and activator of transcription 3 (STAT3) pathway, therefore reducing inflammation and enhancing recovery following SCI (Ren et al., 2023). Concurrently, mesenchymal stem cells (MSCs) have shown the capability to affect macrophage plasticity, offering novel strategies to promote spinal cord regeneration and regulate immune responses (Fu et al., 2023).

#### Role of M1-polarized macrophages in spinal cord injury

Neuronal death, axonal rupture, neurite retraction, and demyelination constitute critical pathological substrates of spinal cord dysfunction. Notably, neural cell death and demyelination are mechanistically linked to macrophage activity in SCI. M1-polarized macrophages extensively release pro-inflammatory cytokines (e.g., IL-1 and TNF-α) and increased ROS post-SCI, inducing oxidative stress, resulting in oxidative stress, extensive demyelination, and neuroinflammatory responses in spinal tissues. Lentivirus-mediated suppression of microglial M1 polarization has been shown to significantly attenuate neuroinflammation and enhance functional recovery. For example, lentiviral targeting of mucosa-associated lymphoid tissue lymphoma translocation protein 1 in rat SCI models reduces M1-polarized microglia during injury, establishing a neuroprotective immune microenvironment (Zhang et al., 2023b). Additionally, M1 macrophages inhibit post-SCI neural regeneration through alternative mechanisms. Chondroitin sulfate proteoglycans, established axonal growth inhibitors, activate TLR4 receptors on microglia/macrophages, impeding M1-to-M2 phenotypic conversion and perpetuating pro-inflammatory M1 dominance, thereby severely obstructing neural regeneration. Consequently, reducing M1 macrophage infiltration in SCI lesions is essential for mitigating neurite retraction and neuronal loss (Yang et al., 2024b). Furthermore, M1 macrophages express elevated leukotrienes, bioactive lipids synthesized via 5-lipoxygenase and cyclooxygenase pathways, that mediate secondary inflammatory cascades post-SCI. These leukotrienes suppress regenerative capacity at injury sites while exacerbating localized inflammation. An *in vitro* study confirmed that M2 macrophage-conditioned media promote robust neurite outgrowth in dorsal root ganglion neurons, whereas M1-conditioned media induce stunted, aberrant neurites with pathological morphology (David et al., 2015).

#### Role of M2-polarized macrophages in spinal cord injury

In undamaged spinal cord tissues, most macrophages display an M2 phenotype, recognized for its properties that reduce inflammation. *In vitro*, activated macrophages demonstrate reduced expression of pro-inflammatory cytokines (e.g., TNF-α and IL-12) following myelin phagocytosis (Freyermuth-Trujillo et al., 2022). Post-SCI, M2 macrophages, derived from stimulated microglia or phenotypic conversion of M1 macrophages, are critical for mitigating localized pro-inflammatory microenvironments. These M2-polarized cells downregulate pro-inflammatory cytokines while upregulating arginase-1 expression, thereby fostering neural regeneration (Wang et al., 2023a). The detrimental consequences of insufficient M2-mediated inflammation resolution post-SCI are evident: persistent inflammation compromises neuronal viability, impedes functional recovery, and exacerbates secondary damage (Tang et al., 2023a). Consequently, modulating macrophage polarization is imperative. Schwann cell-derived exosome composite patches have been demonstrated to shift macrophage polarization from pro-inflammatory M1 to anti-inflammatory M2, with *in vitro* studies showing increased M2 proportions and reduced neuronal apoptosis. *In vivo*, these patches enhance neuronal survival by suppressing inflammation and apoptosis (Zhu et al., 2023a). Furthermore, M2 macrophages activate the mitogen-activated protein kinase (MAPK)/c-Jun N-terminal kinase signaling pathway to promote neural stem cell (NSC) differentiation into neurons, facilitating SCI repair and motor recovery (Gensel and Zhang, 2015). Concurrently, M2-derived exosomes induce microvascular endothelial cells to express angiogenesis-related genes, stimulating neovascularization to enhance nutrient/oxygen supply and metabolic waste clearance, thereby optimizing the regenerative milieu (Guo et al., 2024a). These exosomes additionally downregulate apoptotic markers while upregulating anti-apoptotic proteins, further preserving neurons and improving locomotor function. The neurorestorative capacity of M2 macrophages and their exosomes underscores the critical role of immune modulation in SCI recovery. By transitioning the microenvironment from pro-inflammatory to pro-regenerative, M2 macrophages confer neuroprotection and stimulate repair mechanisms. Enhancing M2 activity and leveraging their exosomes may thus represent a promising therapeutic strategy to improve SCI rehabilitation outcomes (Zhu et al., 2023a).

#### Temporal dynamics of macrophage action in the different stages of spinal cord injury

Within minutes of SCI onset, a cascade of cellular events initiates, with calcium dysregulation emerging as one of the most critical and immediate early consequences. Under physiological conditions, intracellular and extracellular calcium concentrations are tightly regulated to maintain cellular functions. However, SCI disrupts the homeostatic control, triggering massive calcium influx into cells that exceed physiological regulatory capacity. The calcium overload induces plasma membrane permeability alterations and precipitates ischemic damage in adjacent tissues. Intracellular calcium accumulation activates multiple deleterious pathways, including proteolytic enzyme activation, oxidative stress, and mitochondrial dysfunction. These pathological cascades ultimately lead to the death of neurons and glial cells, resulting in functional cell loss within SCI lesions. Early-stage mass cell death not only impedes primary repair mechanisms but also exacerbates secondary damage, constraining recovery potential (Brennan et al., 2022). During the acute SCI phase, microglia, the resident macrophages of the CNS, exhibit minimal immune reactivity. In this period, they predominantly maintain a quiescent state, functioning to preserve neuronal microenvironmental homeostasis by actively surveilling neuronal integrity and suppressing excessive immune activation, thereby preventing collateral damage to vulnerable injured spinal tissues.

Within 3 to 6 hours post-SCI, localized microglia and macrophages undergo rapid activation and commence secretion of pro-inflammatory cytokines such as TNF-α, IL-6, and IL-1β. The release of these cytokines exacerbates inflammatory responses, amplifies immune recruitment, and further propagates microglial and immune cell proliferation. Sustained elevation of pro-inflammatory mediators aggravates tissue damage and delays repair processes. Notably, distinct macrophage subtypes exhibit differential responsiveness to specific cytokines and fulfill unique functional roles. For instance, interferon-gamma (IFN-γ)-activated macrophages demonstrate neurotoxic properties that intensify neuronal injury, whereas IL-10-stimulated macrophages exhibit anti-inflammatory characteristics, protecting tissues from excessive immune reactivity and accelerating tissue repair by promoting wound healing (Kopper et al., 2021; Hu et al., 2023b). The balance between pro-inflammatory and anti-inflammatory responses is essential for effective immunological regulation and functional recovery following SCI.

Within the first 2 days post-initial SCI, axonal retraction commences, exacerbating the injury and amplifying neurological deficits. Concurrently, monocytes from the circulation move to the lesion site and develop into macrophages within the local microenvironment. These newly differentiated macrophages are crucial in both inflammatory responses and tissue repair processes post-injury. Among key cytokines regulating macrophage activity, IL-4 and IL-10, serve critical roles in promoting macrophage activation toward an anti-inflammatory phenotype. The phenotypic activation is important for initiating tissue repair and regeneration within the injured CNS. Morphologically and phenotypically, these PDMs closely resemble activated microglia post-injury. During the acute phase, microglia recruit PDMs to infiltrate the lesion area. Through cytokine release, these macrophages participate in initiating inflammatory responses and modulating the local immune microenvironment. When appropriately regulated, the inflammatory response facilitates the healing of tissues and restoration of function (Ma et al., 2024; Peng et al., 2024).

SCI originates from resident microglia and PDMs the latter being bone marrow-derived cells that infiltrate the lesion site post-SCI. By day 5 post-injury, PDMs and activated microglia emerge as the predominant inflammatory cell populations, with PDMs predominantly localized to necrotic regions. At the lesion border, a dense cellular interface forms between reactive astrocytes and infiltrating PDMs, where microglia undergo robust proliferation.

**Figure 2:**
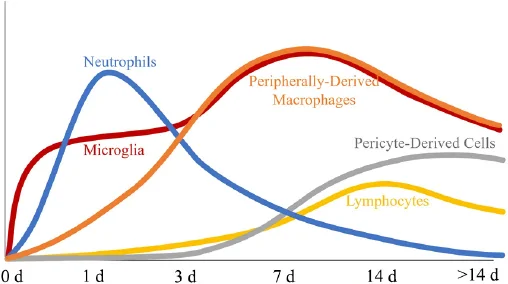
Primary injury of spinal cord injury causes cell membrane disruption and rupture of blood vessels leading to secondary injury with extensive upregulation of cytokines/chemokines and infiltration of immune cells. Microglia exhibit rapid activation with morphological convergence toward peripheral macrophages. Rat injury models demonstrate neutrophil dominance (24 hours peak, declining over 7–10 days) versus delayed lymphocyte infiltration at lower magnitudes. Pericyte migration manifests during late-phase responses. Reprinted from Hellenbrand et al. (2021).

During the first 7 days post-SCI, chemokine levels are markedly elevated, exacerbating neuroinflammation. Key mediators include MIP-1α, which shows increased expression on days 1, 3, and 7 post-SCI (Freyermuth-Trujillo et al., 2022), driving the recruitment of dendritic cells, T cells, and monocytes to lesion sites, thereby amplifying the extent and intensity of inflammatory responses (Freyermuth-Trujillo et al., 2022). The process is orchestrated by a dynamic cytokine network that modulates inflammatory cell recruitment/activation, ultimately establishing a complex immune microenvironment at SCI sites (Hellenbrand et al., 2021; **Figure 2**).

Macrophages are essential immune cells in the CNS and play a significant role in SCI recovery. They serve as the principal source for the absorption of myelin-derived cholesterol, an essential lipid that facilitates axonal regeneration. Following SCI, macrophage activation peaks between 7 and 14 days post-injury, which is crucial for tissue repair. Macrophages and microglia are highly polarizable cells that can shift between two different phenotypes: M1 and M2. The balance between these two phenotypes influences the local immune microenvironment. M1 macrophages are generally pro-inflammatory, and their presence can be detrimental to healing by releasing large amounts of inflammatory cytokines. They dominate the acute phase after SCI due to the high levels of ROS that worsen tissue destruction. However, M1 macrophages can transition to an M2 phenotype by processes like phagocytosing myelin sheaths, which give them anti-inflammatory properties and aid in tissue repair. In a mouse model of SCI, researchers have noted that macrophages correlate with the spatial distribution of platelet-derived growth factor receptor beta-positive pericytes at the damage epicenter on days 3, 7, and 14 post-trauma. Macrophage is M2 polarized at 3 and 7 days after injury, while macrophage transition to an M1-polarized state at 14 days post-injury. M2 can secrete recombinant platelet derived growth factor subunit B, which in turn acts on PDGFRβ and promotes the migration of PDGFRβ+ pericytes, which then has a negative effect on SCI repair negatively (Li et al., 2021b). However, the low number of M2 macrophages early on in SCI, which decreases even further after 7 days, is negatively affected by the injury microenvironment. It limits the conversion of M1 to M2 (Wu et al., 2022).

Myelin, being lipid-rich, necessitates timely clearance of excessive myelin debris post-SCI to prevent lipid peroxidation at lesion sites. Studies have demonstrated that modulating macrophage phagocytic activity via the IL-3/IL-3 receptor subunit alpha pathway enhances lipid clearance and promotes SCI repair, validating the therapeutic efficacy of myelin debris removal (Li et al., 2024b). However, excessive phagocytic removal of myelin fragments disrupts intracellular lipid homeostasis, triggering foam cell formation that exacerbates inflammatory responses and worsens neurological deficits. Although early investigations failed to effectively prevent foam cell formation to reduce neuronal apoptosis, they highlighted the potential value of inhibiting the process for SCI management. A recent study employs suppression of receptor-independent endocytosis to inhibit or reverse foam cell generation, which not only reduces neuronal apoptosis but also enhances axonal regeneration (Zhang et al., 2025a). Consequently, balanced clearance of myelin debris and prevention of foam macrophage emergence post-SCI is critical for neural repair.

During this phase, MHC II is expressed by macrophage, which also produces anti-inflammatory cytokines like transforming growth factor (TGF)-β1 and IL-10. Additionally, macrophage upregulates the expression of important enzymes that are necessary for the creation of pro-resolving mediators, which effectively reduces inflammation. Monocyte-derived macrophages are crucial for removing myelin debris. Studies in a substantial rat epidural balloon crush model of SCI have demonstrated that macrophages persist in phagocytosing myelin at the injury location for as long as 16 weeks after the incident. They engage in the removal of non-conditioned and conditioned myelin particles through receptor-mediated phagocytosis within the spinal cord, thereby facilitating myelin regeneration (Van Broeckhoven et al., 2021).

The initial phase of cellular inflammation post-SCI is predominantly characterized by macrophages/microglia, neutrophils, and T cells. During the final stage of inflammation, which occurs 14 days after the injury, these three cell types remain detectable and continue their presence up to 180 days post-injury, with macrophages/microglia reaching their peak at 60 days post-injury. Moreover, the late stage of cellular inflammation is not consistent with motor function outcomes as measured by open-field testing in animal models following SCI. Nonetheless, blocking the chemokine C5a-driven inflammatory response 14 days post-SCI hinders motor function recovery and myelination in the damaged spinal cord, suggesting that the late inflammatory response also plays a reparative role (Beck et al., 2010). Unfortunately, our understanding of the specific role of macrophages in the remodeling phase following SCI remains limited. Current literature, as indicated by the search, offers few studies that explore the role of macrophages across different stages of tissue repair, which could further elucidate the macrophages’ role post-SCI.

#### Modulation of macrophage polarization homeostasis affects spinal cord injury regression

The surrounding environment of the damaged spinal cord undergoes a series of severe pathological changes, including neuronal degeneration, endothelial cell damage, myelin disintegration, and activation of glial cells and other immune cells, which collectively drive the production of additional pro-inflammatory mediators (Ma et al., 2024). Following SCI, immune and glial cells collaboratively establish a defensive shield that facilitates tissue repair, wound closure, debris clearance, and suppression of inflammatory responses, thereby generating a dense extracellular matrix (Zhou et al., 2020b). SCI-associated inflammation can be categorized into distinct phases: neutrophil stimulation and infiltration, monocyte recruitment, and scar resolution via anti-inflammatory macrophages and axonal regeneration. Studies demonstrate that a distinct neuroinflammatory response persists throughout the secondary injury phase post-SCI. The stimulation of glial cells triggers the secretion of pro-inflammatory factors, accelerating neural deterioration through the cascade mechanism. Concurrently, the process prompts vascular endothelial cells to express cell adhesion molecules and chemokines, recruiting additional inflammatory factors. Circulating monocytes differentiate into phagocytic macrophages, which, alongside microglia, constitute the first line of innate immune defense. Their roles involve clearing cellular debris and providing nutrients and anti-inflammatory factors critical for promoting tissue repair. However, these cells also secrete pro-inflammatory cytokines, leading to exacerbation of secondary damage (Wang et al., 2023a).

Following SCI, macrophage polarization within the pathological microenvironment becomes imbalanced. At the lesion site, M1 and M2 macrophages exhibit a relatively balanced distribution, displaying a hybrid functional phenotype (hybrid-macrophage). These macrophages co-express elevated levels of M1 markers (e.g., inductible nitric oxide synthase (iNOS)), M2 markers (e.g., arginase and chitinase 3-like 1 (Ym1)), and pro-inflammatory mediators such as IL-6. Concurrently, TNF-α, IL-12, and IL-1β levels surge acutely post-SCI. Macrophages in the hybrid state fail to effectively clear damaged tissues and neutrophils, while their depolarized accumulation disrupts the self-limiting inflammatory process. Over time, macrophages ultimately polarize toward the M1 phenotype, peaking 7–14 days post-injury (Sun et al., 2016).

Multiple studies demonstrate that modulating NF-κB, Janus kinase (JAK), STAT, phosphatidylinositol 3-kinase-protein kinase B (Akt), and Notch signaling pathways can influence macrophage polarization to improve SCI outcomes (Wang et al., 2023a). Research by Gu and colleagues revealed that the angiotensin-(1–7)/G protein-coupled receptor Mas axis plays a pivotal role in mitigating inflammation and oxidative stress. By elevating angiotensin-(1–7) levels at injury sites, they promoted the phenotypic shift of macrophages from pro-inflammatory M1 to anti-inflammatory M2, thereby attenuating the inflammatory microenvironment in SCI (Gu et al., 2023). Similarly, Zhang et al. (2025b) utilized biomimetic conductive aligned nanofiber mats to upregulate cytochrome c oxidase subunit 5A and STAT6, while downregulating IL-1β, CD36, phosphorylated extracellular signal-regulated kinase, nuclear factor kappa B subunit 2 (NF-κB2), and NF-κB signaling, driving macrophage polarization toward the M2 phenotype to ameliorate SCI-related inflammation. Guo et al. (2023) identified that inflammatory cytokines such as IL-6, TNF-α, and IL-1β exacerbate secondary SCI by polarizing macrophages and microglia post-injury. Within cytokine responses, the JAK/STAT pathway serves as a critical signaling mechanism from the extracellular milieu to the nucleus. Upon cytokine binding to membrane receptors, glycoprotein dimerization occurs, activating JAK and inducing STAT1 phosphorylation in the cytoplasm. Phosphorylated STAT1 translocate to the nucleus to initiate transcription of target genes. Post-SCI pathophysiological changes primarily occur via this pathway. Additionally, tauroursodeoxycholic acid has been shown *in vitro* to suppress NF-κB signaling while activating Akt and cyclic adenosine monophosphate pathways, reducing apoptosis. Hou et al. (2025) confirmed that tauroursodeoxycholic acid significantly decreases monocyte/macrophage infiltration, alters their distribution, and promotes M2 polarization, thereby improving the injury microenvironment and restoring normal migration/proliferation of spinal NSCs to enhance neural regeneration post-SCI.

In summary, macrophage polarization is critical for modulating inflammatory responses and promoting neural regeneration following SCI. At lesion sites, the balance between M1 and M2 macrophages becomes disrupted, leading to prolonged inflammation and secondary damage. Targeting signaling pathways such as NF-κB and JAK/STAT to regulate macrophage polarization holds significant therapeutic potential. By promoting M2 polarization while suppressing M1 activation, it is possible to attenuate inflammation, enhance tissue repair, and improve functional recovery. Thus, macrophage modulation represents a pivotal strategy for advancing SCI treatment and optimizing patient outcomes.

### Role of macrophages in different types of spinal cord injury

Generally, SCI can be classified into primary SCI and secondary SCI according to the degree and mechanism of injury. However, due to the different types of injury, such as contusion, extrusion, pull, dislocation, transection, ischemia, tumor, infection and so on, macrophages play a variety of complex roles in the process of different types of SCI.

#### Role of macrophages in primary spinal cord injury

Primary SCI is primarily caused by mechanical forces, such as fractures or dislocations, leading to direct physical damage to the spinal cord tissue. Within a short time after injury, macrophages are activated. Resident macrophages, specifically microglia around blood vessels, are the first to detect damage signals. They recognize DAMPs through pattern recognition receptors (PRRs) on their surface. These DAMPs include adenosine triphosphate and IL-33, which are released due to cell damage and death. These compounds stimulate PRRs, inducing pathogenic self-reactivity such as the release of extracellular matrix components, and high mobility group protein 1 in damaged cells.

A study has shown that the microglia in the initial inflammatory response of injury will undergo morphological changes after being activated (Green and Rowe, 2024). They shift from their ramified resting state under normal conditions to an active “fighting” state, ready to perform immune functions. Activated microglia release pro-inflammatory cytokines such as IL-1, IL-6, and TNF-α. It initiates a local inflammatory response. While controlled inflammation aids in clearing cellular debris, excessive inflammation exacerbates tissue damage (Scherlinger et al., 2023). The inflammatory cascade further induces chemokine production, driving the infiltration of macrophages and neutrophils alongside complement system activation, culminating in amplified neuroinflammation (Saad et al., 2025). If left unregulated, it hyperactivated state inflicts additional harm on the vulnerable of spinal cord, limited-regenerative-capacity neural tissue, posing significant challenges for therapeutic intervention and functional recovery.

Macrophages are mainly involved in initiating the inflammatory response during primary SCI (Chen et al., 2024). The priority is to combat pathogens and remove damaged tissue debris. Effective defense mechanisms must be established quickly to restore tissue integrity. Although microglia-mediated phagocytosis is crucial for shaping cell structure (Beiter et al., 2024), it is limited in clearing neural tissue fragments during primary SCI. The sudden and severe injury leads to a chaotic local environment with tissue fragments and bleeding. Additionally, damage to the blood-spinal barrier allows blood components to enter the spinal cord tissue, interfering with macrophage phagocytosis (Cui et al., 2024). Thus, macrophages focus on maintaining the inflammatory response initially, only shifting to phagocytosis and cleaning of neural debris once the environment stabilizes.

Further research has revealed that primary SCI triggers a cascade of secondary pathological alterations, including hemorrhage, ischemia, thrombosis, oxidative stress, inflammation, apoptosis, and scar formation. Among these secondary injury mechanisms, hyperactivation of systemic immune responses and neuroinflammation emerge as pivotal determinants of injury severity (Hu et al., 2023b). Through integrated animal experimentation, clinical observation, and histopathological analysis, the classification of SCI has been refined based on injury severity and mechanisms, providing a critical theoretical framework for developing therapeutic strategies and mechanistic investigations.

#### Role of macrophages in secondary spinal cord injury

After initial mechanical injury causes bleeding and cell death, secondary damage can occur through cascading reactions such as apoptosis, blood flow disruption, free radical generation, and inflammation (Wang et al., 2023a). These secondary injuries can significantly impede tissue healing and exacerbate functional deficits (Wu et al., 2025). However, macrophages in secondary SCI can regulate the body activities through their diverse functions.

Secondary SCI refers to further damage that occurs hours to days after the primary injury. A key function of macrophages is to remove cell debris and facilitate tissue remodeling. Microglia and macrophages, as specialized phagocytes, play a crucial cleaning role at this stage (Poppell et al., 2023). These cells phagocytose cellular fragments and myelin residues, promoting regenerative microenvironments. Macrophages act as a protective shield against neuronal and glial cell fragment toxicity through phagocytosis, while also emitting neurotrophic factors (Yamanaka et al., 2023). Such elements enhance the survival of neurons, stimulate the differentiation of NSCs, and augment the count of neurons (Zheng et al., 2024), and stimulate axonal growth to rebuild neural connections (Hashimoto et al., 2023; Zhang et al., 2023a). Generally, the combined effect of phagocytosis and nutrient emission establishes macrophages as a key cell variety in repairing SCI.

In secondary SCI, macrophages are key regulators of angiogenesis, secreting elements like vascular endothelial growth factor to encourage the development of blood vessels in the affected region (Peng et al., 2023). Vascular remodeling critically supports spinal cord repair through enhanced oxygenation, nutrient delivery, and metabolic clearance, thereby aiding overall healing.

#### Role of macrophages in different types and complications of spinal cord injury

Various causes can lead to SCI, such as dull pressure, bone breaks, stretching, muscle ischemia, etc. Although these injuries have distinct causes, they collectively initiate macrophage activation. Initially, macrophages take on a pro-inflammatory form and emit cytokines like TNF-α and IL-4 to draw. Gradually, they evolve into an anti-inflammatory M2 phenotype, facilitating tissue healing and axonal renewal. The M1-to-M2 shift is uniform among all SCI varieties, yet its specific functions may differ based on distinct pathological characteristics.

In contusive SCI caused by localized mechanical compression, macrophages initially function as pro-inflammatory cells, releasing cytokines such as TNF-α and IL-4 to initiate immune responses, followed by gradual transition toward pro-regenerative M2 phenotypes (Ding et al., 2023). Conversely, compressive injuries induced by fractures or disc herniation are often accompanied by prolonged ischemia and hypoxia, creating a microenvironment detrimental to later-stage repair (Sun et al., 2023b). In stretch injuries resulting from spinal hyperflexion or traction, macrophages clear damaged tissues and support neural repair through neurotrophic factor secretion (Mosser et al., 2021). For subluxation injuries involving vertebral misalignment, macrophages release pro-inflammatory mediators to eliminate damaged tissue, though chronic fibrotic responses may constrain neural regeneration (Yu et al., 2022). Injuries with complete spinal cord rupture represent the most severe form, where macrophage-mediated necrotic debris clearance occurs, yet substantial repair remains unattainable due to anatomical discontinuity. Current research focuses on modulating macrophage functions to enhance axonal regeneration at injury sites (Huang et al., 2024). Ischemic SCI caused by vascular disruption induces localized hypoxia and cellular death; here, macrophages mitigate ischemia by phagocytosing necrotic cells and secreting angiogenic factors (Tang et al., 2023b). In infectious SCI, macrophages phagocytose pathogens and damaged tissue to reduce inflammation. However, polarization disequilibrium between M1/M2 macrophages may exacerbate inflammatory cascades, further damaging spinal tissues (Marrufo and Flores-Mireles, 2024).

In studies of SCI, the role of macrophages is usually focused on repairing damage and modulating the immune response. However, in the spinal cord tumor environment, the role of macrophages is more complex. Tumor-associated macrophages (TAMs) play a dual role in tumor development: on one hand, they are able to exert an anti-tumor effect by recognizing and phagocytosing tumor cells; on the other hand, tumor cells inhibit the normal function of macrophages by secreting cytokines, prompting them to transform into TAMs, and these transformed macrophages support tumor growth and metastasis instead. Therefore, the dynamic interaction between TAMs and other immune cells is considered an important target in future tumor immunotherapy. Modern medicine can modify the role of macrophages in tumor development by modulating their number and phenotype, and thus develop a variety of therapeutic strategies targeting macrophages to influence tumor growth and progression (Lu et al., 2024a).

Primary and secondary injuries after SCI can lead to a variety of sensory/motor complications such as neuropathic pain, heterotopic ossification, stress injuries, and excretory dysfunction (Irgens et al., 2020; Yolcu et al., 2020; Gong et al., 2021; Sun et al., 2023a). Among them, neuropathic pain is the most common and important symptom after SCI, which is highly likely to cause debilitating and even depressive psychiatric symptoms (Miranpuri et al., 2021; Richards et al., 2024). However, its specific mechanism is currently unclear. However, increasing evidence suggests that the development of neuropathic pain may be closely related to microglia/macrophages-mediated neuroinflammatory responses after SCI (Yang et al., 2023), and that pro-inflammatory mediators released by these cells play a key role in pain signaling (Miranpuri et al., 2021; Widerström-Noga, 2023; Hua et al., 2024). Macrophages with two different inflammatory profiles after SCI can differentially affect local neuronal circuits (Richards et al., 2024), and their alterations in different cellular functions and interactions with surrounding cells can directly modulate neuronal intrinsic properties (Kohno and Tsuda, 2025). In other words, microglia/macrophages have a dual role in neuropathic pain after SCI, and their different polarization phenotypes are key factors (Sun et al., 2023a). For the M1 phenotype: pro-inflammatory macrophages secrete pro-inflammatory factors and injurious mediators that act on the receptors of pain projection neurons and interneurons in the dorsal horn of the spinal cord, triggering their depolarization and thus enhancing nociceptive signaling. Therefore, M1 infiltration at the site of SCI injury far beyond M2 macrophages may inhibit SCI functional recovery and cause chronic neuropathic pain (Nakajima et al., 2020). For the M2 phenotype: Due to the powerful anti-inflammatory and tissue-repairing effects of the M2 phenotype (Shahrezaei et al., 2024), increasing the polarization of the M2 phenotype of macrophages may significantly ameliorate a variety of complications caused by neuroinflammation triggered after SCI, especially neuropathic pain (Zhu et al., 2023b). A study indicated that red light at 670 nm did not lead to upregulation of M1 phenotype, but increased the proportion of M2 macrophages, which could effectively improve neuropathic pain during the subacute phase of SCI (Hu et al., 2020). Increased programmed cell death ligand 1 expression after SCI could promote increased M2 phenotype through phosphorylation of p38 and extracellular regulated protein kinases, improving neuropathic pain, and contributing to the functional recovery after SCI (Kong et al., 2021). Resolvin D2 promotes macrophage polarization towards the M2 phenotype by down-regulating the NF-κB/miR-155 signaling pathway, thereby reducing neuroinflammation and neuropathic pain after SCI (Yang et al., 2024a). Interestingly, a study found that macrophages act differently for neuropathic pain in different spinal cord segments, with functional recovery of neuropathic pain observed only in the cervical vertebra but not in the lumbar vertebra (Brown et al., 2021). Further experiments are worthwhile to verify the role of the two phenotypes for neuropathic pain at different neuropathic pain sites. Metabolic reprogramming of macrophages plays a major role in regulating neuroinflammation (Richards et al., 2024), but there is no evidence yet on how altered polarization phenotypes contribute to neuropathic pain development following SCI through metabolic reprogramming. Furthermore, when exploring the association between macrophage phenotypic characteristics and neuropathic pain after SCI, the dynamic changes in macrophage polarization phenotype should be clarified in the context of the differences in the predominant macrophages infiltrated at different times after SCI to gain a deeper understanding of neuro-immune interactions.

## Modulating Macrophage Activation and Polarization Influences Spinal Cord Injury Repair

### Activation and polarization of macrophages

In the body, macrophages in a resting state recognize pathogen-associated molecular patterns, DAMPs, cytokines, and other environmental signals. The recognition activates intracellular signaling pathways, governing transcription and translation to fulfill immunological activities (Shapouri-Moghaddam et al., 2018). Macrophage activation can be categorized into pro-inflammatory (classical activation or M1) and anti-inflammatory (alternative activation or M2) kinds, contingent upon the triggering events, which exhibit distinct surface receptors, secretion profiles, and functional characteristics.

Compared to the resting state, activated macrophages undergo significant changes in function and phenotype. Morphologically, activated macrophages display increased cell volume, pseudopodia formation, and enhanced membrane dynamics (Mylvaganam et al., 2021), which improve their phagocytic capacity, enabling more efficient migration and phagocytosis of pathogens or apoptotic cells. Also, stimulation upregulates surface PRR density. For example, in response to bacteria, viruses, or fungi, the expression of PRRs, such as TLRs and C-type lectin receptors increases (Gensel et al., 2015). Meanwhile, during the clearance of apoptotic cells or endogenous damage molecules, scavenger receptors (such as CD36 and scavenger receptor A) play a key role (Kazakova et al., 2022). The enhanced expression of these receptors facilitates the recognition and uptake of both foreign antigens and apoptotic cells.

Antigen presentation is a critical function of macrophages. In the activated state, lysosomal function is enhanced, allowing for more efficient degradation of antigens into small peptide fragments. Activation stimulates the transcription and translation of lysosome-associated protein molecules, such as lysosome-associated membrane proteins 1 and 2 (Zhang et al., 2021b). Through the regulation of RAB family small GTPases (e.g., RAB7) and soluble N-ethylmaleimide-sensitive factor attachment protein receptor, the efficiency of phagosome-lysosome fusion is improved (Taefehshokr et al., 2021), expediting the degradation of phagocytic particles and antigen processing.

MHC I and MHC II molecules are greatly expressed in active macrophages. MHC I primarily present endogenous antigens to CD8^+^ T lymphocytes, which leads to cytotoxic T cell immune responses (Colbert et al., 2020). The upregulation is associated with cytokines such as IFN-γ (Wang et al., 2024g). MHC II delivers external antigens, such as bacteria or their pieces, to CD4^+^ T lymphocytes, promoting T cell activation in adaptive immunity. The enhanced expression of MHC II molecules is driven by classical activation signals (e.g., lipopolysaccharide (LPS) and IFN-γ) via STAT1 and NF-κB pathways (Mussbacher et al., 2023). The significant increase, particularly in MHC II, amplifies macrophages’ function as APCs.

The elevated production of substances that stimulate each other is a characteristic of activated macrophages functioning as APCs. These molecules provide the “second signal” that synergizes with the “first signal” presented by MHC molecules, effectively activating T cells and enhancing adaptive immune responses (Kumar et al., 2018). Key co-stimulatory molecules include CD86 (B7-2), CD80 (B7-1), and CD40. CD86 CD80 and interact with CD28 on T cells, delivering the “second signal” vital to the activation of T cells (Bonelli and Scheinecker, 2018). CD40, in addition to binding CD40L on T cells to enhance T cell activity, recruits TRAF proteins (e.g., recombinant TNF receptor associated factor 2, TNF receptor-associated factor 6) via its intracellular domain. It activates the MAPK and NF-B pathways, which causes macrophages to secrete huge amounts of cytokines (such as IL-6, IL-12, TNF-α) and chemokines (such as C-C motif chemokine ligand (CCL)5, CCL2) (Tian et al., 2024), which attract other immune cells (like T cells and neutrophils) to the infection area (Tian et al., 2024).

### Plasticity of macrophage polarization regulates the inflammatory response

**Figure 3:**
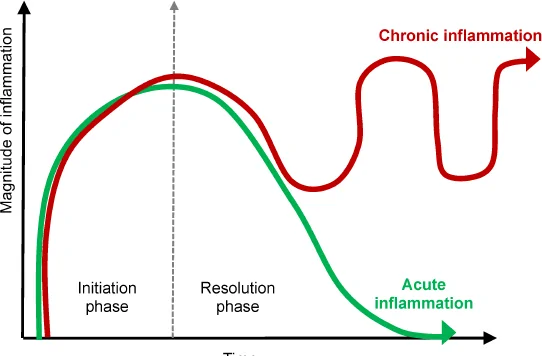
Dynamics of inflammation after spinal cord injury. The acute phase inflammatory cascade constitutes an evolutionarily conserved self-limiting homeostatic mechanism, typically achieving complete resolution under conditions of physiological homeostasis. Dysregulation of these resolution pathways induces persistent inflammatory microenvironments characterized by maladaptive tissue restructuring, extracellular matrix dysregulation, and subsequent clinical pathogenesis. Reprinted from Oronsky et al. (2022).

Inflammation serves as a cornerstone of the host’s defense arsenal, effectively neutralizing pathogens and facilitating tissue repair, yet concurrently posing a risk to healthy tissues due to the noxious molecules emitted by inflammatory cells (Freyermuth-Trujillo et al., 2022). The process is typically divided into acute and chronic inflammation according to the disease course (**Figure 3**). Acute inflammation helps clear debris, pathogens, and dead cells from damaged tissue, occurring mainly in the early phases of injury with a rapid onset and resolution within days. Chronic inflammation, however, is a persistent response due to continued immune cell recruitment, molecule accumulation, or autoimmune reactions, often difficult to resolve (Hassanshahi et al., 2019). However, disturbances in the statement and regulation of various cytokines that cause inflammation involved in acute inflammatory response will lead to a shift in the nature of the inflammation at the site of injury to subacute or even chronic inflammation, thus triggering uncontrollable secondary damage and transformation from granulation tissue to scar tissue, leading to fibrosis (Mata et al., 2021). Neutrophils, macrophages and other immune cells, conversely, a variety of secretions are produced endogenous inflammatory mediators (bioactive lipids, ROS, and nitric oxide [NO]) involved in the context of the inflammatory response (Sikora et al., 2023). Leuti et al. (2020) also have suggested that endogenous bioactive lipids might contribute to the deleterious change in the inflammation from acute to chronic, through imbalances in the four major lipid groups caused by overactivation of arachidonoids, underproduction of specialized pro-resolving mediator, or aberrant activity of the endocannabinoid and sphingolipid-related signaling axes. Thus, immune cells, cytokines, and the inflammation-regulating microenvironment play vital roles in this shift. In-depth study of the inflammatory microenvironment is crucial to inhibit chronic inflammation and promote damage repair. Identifying and targeting key regulatory nodes holds significant clinical importance for managing inflammation-related diseases.

Macrophage is the primary regulator of the inflammatory response. It is now known that if macrophage is depleted in the primary post-injury phase, which can affect the inflammatory stage. The inflammatory response is usually greatly attenuated and results in reduced wound debridement, repair and regeneration efficiency (Zhang et al., 2012). It is evident that macrophages work effectively in the inflammatory response. Tissue-resident macrophages are the prime cells to undergo injury and induce an inflammatory response by releasing hydrogen peroxide. It triggers sequential infiltration of circulating neutrophils followed by monocytes (Li et al., 2021a). Blood-borne macrophage migrates from monocytes in the peripheral blood circulation to the site of damage and is guided by a series of signaling molecules and cytokines to differentiate into M0-type macrophages, which can secrete cytokines through a variety of regulatory mechanisms to polarize them into ‘classically activated’ M1 or ‘alternatively activated’ M2 (Li et al., 2018).

#### M1 macrophage phenotype and function

Classically activated M1 macrophages are initially generated through the polarization of M0-type in response to stimulation such as LPS and IFN-γ. M1 macrophages can trigger an inflammatory response by secreting pro-inflammatory cytokines, like lowered levels of IL-1β, IL-10, and TNF-α (Zhu et al., 2024). M1 macrophages have a potent phagocytic and antigen-presenting ability, which draws neutrophils and intensifies the inflammatory reaction during the initial stages of inflammation. Pro-inflammatory cytokines are released to start the inflammatory response, which is effective in facilitating the removal of damaged tissue (Xia et al., 2022). Soluble protein mediators that change the extracellular matrix and encourage the formation of vascular endothelial cells, such as vascular endothelial growth factor, are also produced (Maldonado-Lasunción et al., 2018). In addition, the creation of ROS, stimulated by M1 macrophage through a series of reactions to scavenge pathogens, has potent antimicrobial and antitumor activity effects. M1 macrophage-mediated early inflammation has a positive impact on debris removal and tissue repair at the site of inflammation (Xia et al., 2023). However, if M1 macrophage is overproduced, large amounts of substances that cause inflammation such as ROS and reactive nitrogen are produced. The situation will likely lead to oxidative stress and deterioration of the lesion site, impairing tissue regeneration and delaying wound healing (An et al., 2021).

#### M2 macrophage phenotype and function

Alternatively activated M2 macrophage is usually formed by polarization through multiple pathways such as activation of STAT6 through IL-4 receptor α and STAT3 by IL-10 receptor (Hu et al., 2023a). Type M2 macrophage can be divided into subtypes: M2a secretes significant quantities of anti-inflammatory cytokines, including IL-4 and IL-13 to initiate the proliferative phase of repair and expresses elevated mannose receptor C-type 1 levels and fibronectin to aid wound healing and fibrotic lesion formation; type M2a triggers the proliferative repair phase by releasing cytokines that reduce inflammation, promoting cell movement and proliferation through the release of Ym1 and arginase, and promoting the start of formation of tissue by secreting growth factors that facilitate the formation of fibrotic lesions and the closure of wounds (Li et al., 2022c). The M2b type expresses high levels of chemokine ligand 1 with a C-C motif and TNF superfamily member 14 and secretes large amounts of the anti-inflammatory IL-10, IL-12, IL-1β and TNF-α to promote Th2 differentiation and humoral immunity (Wang et al., 2019). M2c cells emit large quantities of IL-10, TGF-β, and overexpression of Mer receptor tyrosine kinase, resulting in anti-inflammatory activity and effective phagocytosis of apoptotic cells (Shapouri-Moghaddam et al., 2018). M2d type (also known as TAM) expresses an elevated degree of TGF-β, and IL-10. and constitutes a major inflammatory component in tumor tissue, contributing to angiogenesis and cancer metastasis (Kiseleva et al., 2023). M2 macrophage secretes a wide range of anti-inflammatory cytokines through multiple isoforms, has a strong debris and apoptotic cell clearance capacity, promotes angiogenesis, and inhibits the tissue-damaging effects of over-activated M1 macrophage. M2 macrophage inhibits inflammatory responses by regulating the anti-inflammatory mechanism, and promotes tissue repair and wound healing (Han et al., 2021).

#### Plasticity of macrophage polarization

Macrophages can switch their phenotype to different polarization states in response to different external stimuli. The M1 and M2 phenotypes are not the end stage of their differentiation, but rather a ‘continuum’ of activation states (Atri et al., 2018). It can be repolarized in a specific direction under the regulation of the corresponding gene transcription, and relevant cytokines. Macrophage polarization is a dynamic and counterbalancing process, fluctuating towards either M1 or M2 phenotypes, depending on the tissue microenvironment and changes in the various types of T cell derived cytokines. An *in vitro* study regulating macrophage polarization showed that when growth factors (granulocyte-macrophage colony stimulating factor and macrophage colony-stimulating factor) were switched separately, the M1 and M2 phenotypes could also switch between each other and reversibly undergo functional redifferentiation (Xu et al., 2013). In addition, a monoamine neurotransmitter, serotonin (5-hydroxytryptamine), mediates macrophage phenotype switching by influencing immune cells’ cytokine production (Funes et al., 2018). The plasticity and polarizable features of macrophages directly shadow the inflammatory microenvironment at the scene of the accident, which lays the theoretical basis for therapeutic interventions to alter the phenotype and function of macrophages in different pathological environments (David et al., 2012).

#### Macrophage regulates tissue repair by modulating the M1/M2 phenotypic polarization balance

As noted above, macrophage polarization critically regulates inflammation initiation, progression, and resolution, and has a ‘double-edged sword’ effect. Its anti- or pro-inflammatory properties depend upon the various macrophage phenotypes. In recent years, many studies have achieved some results by regulating specific macrophage phenotypic polarization in the management of several inflammatory illnesses.

Microglia and macrophages act as M1 (pro-inflammatory) and M2 (anti-inflammatory and pro-regenerative) phenotypes. M1 generates macrophage responses and activates, phagocytic, innate and adaptive immune responses. The M2 phenotype releases specific factors such as TGF-β, platelet-derived growth factor, vascular endothelial growth factor, and insulin-like growth factor 1 (Anjum et al., 2020). In the inflammatory situation of acute lung injury, Wali et al. used dexamethasone loaded enhanced polymer nanoparticles responsive to ROS to eliminate excess ROS and prevent the activation of NOD-like receptor family pyrin domain containing 3 inflammatory vesicles, which enhanced M2 phenotypic polarization while suppressing M1 phenotypic polarization, which in turn suppressed infiltration of neutrophils and proteins and inflammatory substances secreted by the lungs, and ultimately alleviated lung injury (Muhammad et al., 2022). In an inflammatory experiment involving acute peritonitis, Liu et al. (2021) utilized lycium barbarum polysaccharides, through NF-κB translocation, dropping NO and pro-inflammatory cytokine expression levels, thereby exerting anti-inflammatory effects and reducing the production of inflammatory substances and damage to a wide range of organs; In the acute inflammatory phase of burns, treatment with isostearyl alcohol increased matrix metalloproteinase 9 expression, significantly lowered TNF-α and IL-6 levels, and elevated the number of M2 macrophages, which attenuated the inflammatory response and promoted wound healing (Qiao et al., 2022).

In addition, chronic inflammation regulation of macrophage has been investigated with some success, e.g. in the treatment of chronic inflammation in metabolic illnesses linked to obesity. Rabeprazole suppresses the separation of macrophage towards the M1 phenotypic and promotes the polarization of the M2 macrophage through activating STAT6 signaling pathways and inhibiting NF-κB pathways, therefore improving glucose tolerance and insulin sensitivity in mice fed a high-fat diet (Li et al., 2024d). Another treatment for persistent colon inflammation is Baitouweng Tang. It can improve pathological damage in the colon by reducing the number of M1 macrophages and the production of pro-inflammatory cytokines such as IL-6, IL-1β, and TNF-α (Xuan-Qing et al., 2021). Sitagliptin phosphate exerts anti-inflammatory effects by shifting macrophage polarization from M1 to M2 phenotypes, a process critically regulated through IL-10 through peroxisome proliferator-activated receptor-γ-dependent suppression of NF-κB and mechanistic target of rapamycin complex 1 (mTORC1) signaling (Hu et al., 2025). He et al. (2025a) found that hyperglycemia exacerbates vascular chronic inflammation in type 2 diabetes by driving macrophage M1 polarization through myocyte enhancer factor 2d alternative splicing-mediated autophagy suppression and potassium calcium-activated channel subfamily M alpha 1 downregulation. In the treatment of inflammation induced by diabetic nephropathy, miR-146-5p-modified human umbilical cord MSCs decreased systemic and local renal inflammation by targeting the recombinant TNF receptor associated factor 2-STAT6 signaling pathway to promote macrophages transition from M1 to M2 phenotype. In the treatment of infection caused by rheumatoid arthritis, inhibition of phosphorylating of NF-κB and p38 by aconite both prevented the conversion to the M1 phenotype, increased nuclear translocation of peroxisome proliferator-activated receptor-γ and STAT2 to promote switching to the M2 phenotype, and attenuated synovial inflammation (Lin et al., 2023).

**Figure 4:**
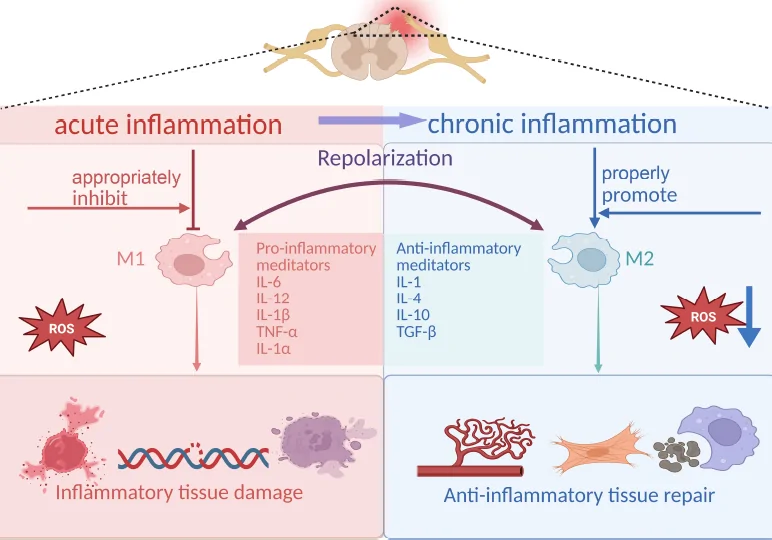
Polarization and plasticity of macrophages after spinal cord injury. Acute inflammation: Pro-inflammatory response dominates (M1), releasing mediators such as IL-6 and TNF-α, leading to tissue damage (e.g., excessive ROS production, extracellular matrix degradation). At this stage, excessive inflammation should be appropriately inhibited. Chronic inflammation: Anti-inflammatory repair dominates (M2), producing mediators like IL-10 and TGF-β, promoting tissue repair (excessive ROS reduction, angiogenesis, extracellular matrix remodeling). Here, the repair process should be promotedin a controlled manner. Transition: “Repolarization” dynamically regulates the M1/M2 balance to shift from tissue damage to repair. Created with BioRender.com. IL: Interleukin; ROS: reactive oxygen species; TGF: transforming growth factor; TNF: tumor necrosis factor.

It can be seen that the reduction of M1 macrophage during the acute inflammatory phase may be to reduce the self-injury caused by excessive tissue clearance and to avoid the conversion of beneficial acute inflammation to harmful chronic inflammation to a certain extent; the reduction of the M1 macrophage and the promotion of M2 macrophage polarization during the chronic inflammatory phase may be to hasten the reduction of the inflammatory reaction while lowering the development of scarring, fibrosis, and secondary damage. Therefore, studying the process of macrophage polarization, recruitment and functional transition, controlling the balance of M1/M2 phenotype, quantity and distribution, inhibiting harmful inflammation and promoting tissue repair, provides new ideas for developing new methods of disease intervention (**Figure 4**).

### Factors regulating macrophage activation and polarization

Macrophages can be activated from the resting state through various regulatory methods such as immune metabolism regulation, transcriptional regulation and epigenetic regulation, and then produce activation phenotypes with corresponding enhanced functions. For example, macrophage activation is closely related to its increased phagocytic capacity (Li et al., 2023b). Immuno-metabolic regulation such as TLR-ligands, LPS and cytokines secreted by T helper-1 lymphocytes such as IFN-γ and TNF-α can activate macrophages to maintain immune responses and confer higher host defense (Marrocco and Ortiz, 2022). As mentioned above, macrophages are mainly recognized by PRRs, such as TLR and NOD-like receptor, which are able to recognize pathogen-associated molecular patterns and DAMPs, thereby subsequently triggering a signaling cascade that transitions macrophages from a resting state to an active state (Yi et al., 2022; Sundaram et al., 2024). In particular, TLR is a key driver of macrophage activation (Yan et al., 2024). TLR modulates gene expression and triggers metabolic reprogramming by activating phosphatidylinositol 3-kinase (PI3K)/Akt, PI3K/Akt/mammalian target of rapamycin (mTOR) and other signaling pathways, thus affecting the activation state and functional transition of macrophages (Kolliniati et al., 2022). Targeting TLR signaling has emerged as a promising therapeutic strategy to modulate macrophage activation and polarization in various diseases (Yan et al., 2024). MicroRNA (miRNA), a crucial modulator of the TLR signaling cascade, influences the function of TLR signaling pathway by controlling the expression of the TLR receptor itself, cascade molecules, adaptor proteins, cytokines, transcription factors and cascade repressor proteins (Rumpel et al., 2024), such as miRNA-21, miRNA-127, miRNA-155 (Sprenkle et al., 2023). In addition, miRNA can also mediate Notch1, STAT3, NF-κB and other signaling pathways to produce downstream effects (Khayati et al., 2023), and activate downstream substances such as IL-10, IL-4, LPS (Sprenkle et al., 2023), thus leading to the activation of macrophages. Different miRNAs have different effects on polarization. For example, miRNA-16, miRNA-19 and miRNA-23a can promote the polarization of M1 cells. miRNA-24, miRNA-32 and miRNA-27a can promote M2 polarization (Khayati et al., 2023; Kovaleva et al., 2023). Current research has established that that ROS generated by mitochondrial electron transport chain is the dominant force in macrophage activation (Geiß et al., 2022). Epigenetic control influences the metabolism and gene expression of macrophages through mechanisms such as lactate, NO-dependent metabolic remodeling and endogenous inflammatory signal reprogramming, thereby regulating their activation state and function. For example, increased lactate levels during glycolysis can promote the activation of microglia by up-regulating the expression of NF-κB, IL-6 and IL-8 in microglia by promoting histone lactate (Pan et al., 2022; Wei et al., 2023).

Based on the clarification of the triggering mechanism of macrophage activation, its polarization is further finely guided by cytokines and metabolic signals in the microenvironment, which ultimately determines the direction of the balance between inflammatory response and tissue repair.

#### Reactive oxygen species

ROS are involved in immune defenses and signal transduction, and are important for the maintenance of homeostasis in the body (Manoharan et al., 2024), including host defense and immune suppression, playing a crucial role in both innate and adaptive immune responses, and enhancing the defensive capabilities of immune cells against pathogens (Liu et al., 2023). Thus, ROS are critical for signaling, polarization, inflammation, and phagocytic activity, especially for macrophages (Shah et al., 2024). During inflammation, elevated ROS concentrations cause immune cell damage and alter macrophage polarization. It has been shown that in inflamed joints, there is an increase in ROS concentration and subsequent activation of inflammatory genes (IL-1β and IL-6), which in turn leads to tissue damage.

ROS are mainly generated by nitrogen oxide, mitochondria and NO synthase. Of these, nitrogen oxide acts as a transmembrane complex, transferring electrons to the phagocytic membrane of macrophage, reducing oxygen to superoxide O_2_^–^, and releasing into vesicles (Tran and Mills, 2024). The important mechanism of mitochondria as the main endogenous source of ROS is oxidative phosphorylation to produce adenosine triphosphate for energy supply. However, generally the energy supply mechanism is counteracted by antioxidant defenses in the mitochondria used to maintain the redox state of the cell. In unpolarized macrophages, NO stimulates ROS production by increasing mitochondrial ROS. During inflammation, ROS production, as well as cytokine production, is exacerbated, all of which alter the macrophage phenotype and lead to further impairment of function in the injured spinal cord. ROS generation leads to increased phagocytic activity and inflammation, which indirectly contributes to the progression of tissue injury, and thus inhibition of the M1 inflammatory phenotype by blocking ROS overproduction is expected to counteract the mitochondrial stress and apoptotic neuronal responses within the injured spinal cord, oxidative stress and neuronal apoptosis (Zhang et al., 2025b).

The effects of ROS on macrophages mainly include the promotion of M1, which demonstrates robust M1 activation characteristics through the specific M1 signaling pathway (NF-κB), which in turn exerts toxic and antimicrobial effects through the production of oxidative substances and modulates the activity of regulatory transcription factors (Geiß et al., 2022). In inflammation, ROS amplifies cytokine production, driving M1 macrophage polarization via NF-κB and PI3K/Akt pathways, which upregulate hypoxia-inducible factor 1 alpha and iNOS. These signaling transcription factors and pathways are essential in maintaining the activated state of macrophage and promoting M1 polarization (Zhong et al., 2023; Wang et al., 2024b). High ROS levels may result in cellular damage and oxidative stress, while also affecting cellular phagocytosis, signal transduction and monocyte/macrophage recruitment.

The function of mitochondrial ROS in macrophage pro-inflammatory signaling during bacterial infection was elucidated for the first time. Thus, the induction of cytoplasmic mitochondrial ROS production through the ETC of assembly complex III is essential for pro-inflammatory signaling. Meanwhile, mitochondrial ROS also modulates the inflammatory response of infected macrophages by influencing NEMO to regulate the inhibitor of kappaB kinase (IKK) complex and promote cytokine secretion (Herb and Schramm, 2021). On the other hand, demonstrated through flow cytometry analysis that M1-polarized bone marrow-derived macrophage treatment significantly increased ROS levels in bEnd.3 cells, and GW4869M1, an exosome secretion inhibitor, partially reversed the increase (Ge et al., 2021). It also confirms that ROS are associated with blood-spinal cord barrier interruption and that regulation of ROS levels plays a critical role in the promotion of functional recovery following injury to the CNS.

In a nutshell, modest quantities of ROS foster immunity and injury healing. However, excessive build-up of ROS at the region of injury disturbs oxidative homeostasis, which leads to oxidation of DNA, lipids and proteins, which then induces apoptosis or death of cells (Zhang et al., 2024b). In addition, abnormally elevated ROS can promote pyroptosis in macrophages, sustaining damage to spinal cord tissues and impeding the repair process. Therefore, eliminating excess ROS by intervening in the antioxidant system *in vivo* can help stop cellular senescence damage and promote inflammation and SCI repair.

#### Interleukin family

IL-4 is a member of the helper T cell factor (Th) 2 family that antagonizes Th1-driven pro-inflammatory immune responses (Bernstein et al., 2023). IL-4 inhibits pro-inflammatory macrophage polarization and promotes M2 anti-inflammatory polarization via STAT6 signaling, clearing pro-inflammatory debris to shape an immunomodulatory microenvironment (Pan et al., 2025). Inflammatory vesicle activation and IL-1β expression can also be inhibited by co-activation of the pathway with peroxisome proliferator-activated receptor-γ, thereby limiting the inflammatory reaction (Hsu et al., 2023; Li et al., 2023a). However, it is still unclear whether IL-4 influences NLRP3 directly or not via macrophages (Li et al., 2023a). Regarding the neuroprotective role of IL-4 in macrophage polarization, findings vary. Li et al. (2025a) showed IL-4-activated extracellular vesicles increase miR-21-5p and suppress programmed cell death 4, driving M1-to-M2 conversion and reducing neuronal apoptosis in SCI. Nevertheless, Liu et al. (2016) showed that IL-4 did not exhibit neuronal protective properties, although it could suppress the M1 phenotype and facilitating the induction of the M2 phenotype polarization. These two studies had the same direction of macrophage polarization but opposite neuroprotective properties, potentially attributable to variations in illness models, induction durations, etc., in which IL-4 acts. Furthermore, it has also been demonstrated that IL-4 even produces neurotoxicity through oxidative stress and polarization of the M2 phenotype macrophage to the M1 phenotype macrophage, exacerbating striatal degeneration (Jang et al., 2022). It suggests that IL-4 may have both anti-inflammatory and pro-inflammatory properties, and points to the fact that the pro-inflammatory causality of IL-4 is mainly due to oxidative stress.

**Figure 5:**
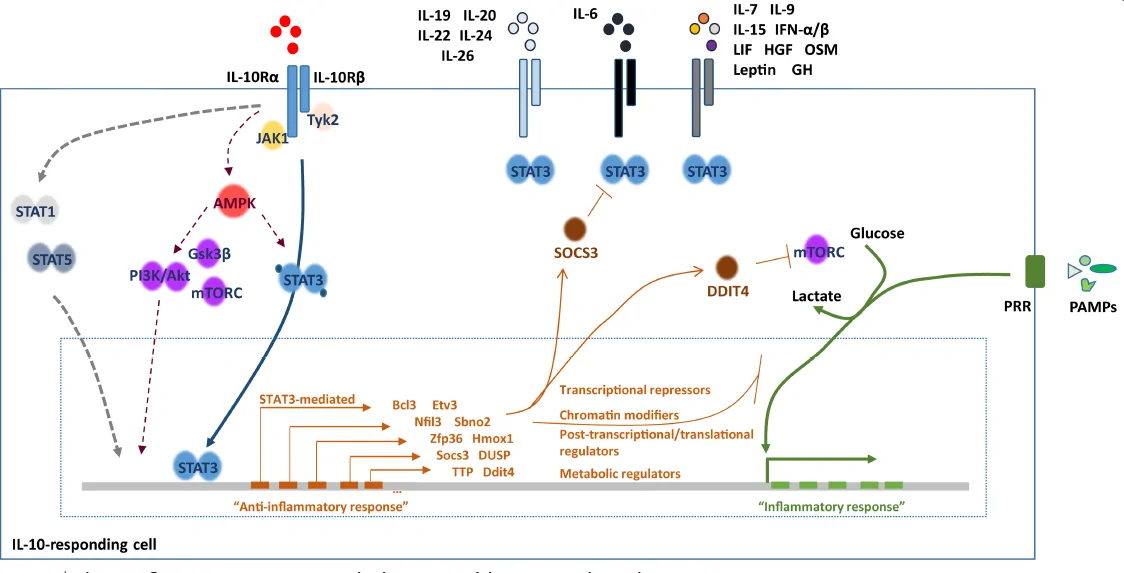
The anti-inflammatory response initiates the downstream of the IL-10R signaling pathway. IL-10 binding to IL-10R triggers JAK1-TYK2-STAT3 signaling, inducing STAT3-mediated anti-inflammatory responses via transcriptional/post-translational regulators that suppress PRR-driven inflammation. STAT3-dependent metabolic reprogramming limits TLR4-induced glycolysis. Alternative pathways involve STAT1/STAT5 and PI3K-Akt, while SOCS3 blocks STAT3 activation specifically downstream of IL-6R but not IL-10R. Reprinted from Saraiva et al. (2020). Akt: Protein kinase B; AMPK: adenosine monophosphate-activated protein kinase; DUSP: dual-specifity phosphatase; Gsk3β: glycogen synthase kinase 3β; GH: growth hormone; GLIK: glycogen synthase kinase; GLUT4: glucose transporter 4; JAK: Janus kinase; IFN: interferon; IL-10R: interleukin-10 receptor; mTORC: mammalian target of rapamycin complex; OSM: oncostatin M; PAMP: pathogen-associated molecular pattern; PI3K: phosphoinositide 3-kinase; PRR: pattern recognition receptor; Sbno2: protein strawberry Notch 2; SOCS: suppressor of cytokine signaling; STAT: signal transducer and activator of transcription; TTP: tristetraprolin; Tyk2: tyrosine kinase 2.

IL-10 is a cytokine that has pleiotropic immunosuppressive properties, such as IL-19, and IL-24. It serves as an anti-inflammatory agent by engaging with the IL-10 receptor, thereby triggering a sequence of downstream reactions (Liu et al., 2024). IL-10 lowers inflammatory cytokines, including IL-6, TNF-α, and IL-1β, and drives macrophage polarization towards M2 macrophages (Patilas et al., 2024). In addition, Wang et al. (2025) found that IL-10-loaded hyaluronic acid methacryloyl hydrogel facilitated a phenotypic shift toward M2 macrophages, as evidenced by a marked upregulation of the anti-inflammatory marker CD206 and concomitant suppression of the pro-inflammatory M1 marker CD86, thereby demonstrating the anti-inflammatory efficacy of IL-10 sustained release (**Figure 5**).

IL-6 is involved in most inflammatory conditions, and is a biomarker of systemic inflammation (Zhang et al., 2024c). It has some anti-inflammatory properties and exerts both pro- and anti-inflammatory impacts on the body (Wang et al., 2024d). For example, Lee et al. (2023) found that using Trichomonas vaginalis-induced IL-6 production by prostate epithelial cells promotes macrophage proliferation and polarization of the M2 phenotype through the IL-6 receptor/JAK signaling pathway. By comparison, other studies have reported that IL-6, as an upstream cytokine, activates STAT3, a major downstream signal transducer, to mediate the production of pro-inflammatory cytokines and M1 macrophage polarization (Chen et al., 2022b; Guo et al., 2024b). Recent research has found that IL-6 promotes the conversion of macrophage to the M2 phenotype only above a certain threshold (5.5 ng/L). When the level of IL-6 falls below the threshold, it is negatively associated with the amount of the M2 macrophage (Wu et al., 2023b). It implies that chronic low-dose IL-6 is pro-inflammatory, whereas short-term/pulsatile high-dose IL-6 is likely to be anti-inflammatory. In addition, IL-6 enhances M1 inflammation in the acute phase and promotes M2 repair in the late phase (Shipman et al., 2024). It suggests that the high concentration and its duration of action may be responsible for the anti-inflammatory and pro-inflammatory duality of IL-6. Most of the pro-inflammatory effects of IL-6 are mediated by trans-signaling pathways, while the anti-inflammatory function is attributed to classical signaling pathways (Fu et al., 2024), and inhibition of trans-signaling in the acute phase reduces inflammation, while promotion of classical signaling in the later stage promotes M2 phenotype-mediated tissue repair (Shipman et al., 2024).

The pro-inflammatory cytokine IL-38 down-regulates M1 macrophage-associated markers of inflammation (TNF-α, iNOS, and cyclooxygenase-2), and inhibits glucose transporter 1 expression, the NF-κB signaling pathway, and the MAPK signaling pathway, resulting in the suppression of M1 polarization (Luo et al., 2023). IL-37 pretreatment decreased the expression of iNOS, IL-6, and TNF-α in LPS-induced RAW264.7 macrophages and argumented the expression of CD206, IL-10, and arginase-1 in IL-4-induced RAW264.7 macrophages, which significantly contributed to the M2 phenotypic polarization (Yang et al., 2024a). These conversions promoted the polarization of macrophages towards the M2 subtype, thereby facilitating functional recovery. In addition, IL-33 activates TLR4 and TLR88 in a myeloid differentiation primary response 2-dependent way, increasing pro-inflammatory chemokines and cytokines and thus exhibiting a pro-inflammatory function (Rai et al., 2022). It is worth pointing out that the different effects of a cytokine on macrophage polarization (M1/M2) versus inflammatory response (pro-/anti-inflammatory) are potentially relevant to the experimental cells studied, the organs, and the experimental treatment of the injury model.

#### Interferon family

The most common type I interferons include IFN-β, IFN-α and IFN-γ. In general, most of the type I interferons are converted towards a pro-inflammatory phenotype in terms of macrophage polarization and exert pro-inflammatory effects. Therefore, under laboratory conditions, in addition to LPS, IFN-γ is usually used to induce macrophage that produces M1 macrophage, resulting in an inflammatory model. It has been shown that IFN-γ increases glycolysis through the JAK2/STAT1 signaling pathway and promotes the polarization of M1 macrophage in vascular epicardial lesions of Takayasu arteritis (Chen et al., 2022a). He et al. (2025b) used fisetin to reduce IFN-γ-induced pro-inflammatory and M1 marker genes, regulated by the JAK1/2-STAT1-interferon regulatory factor 1 pathway. Another study showed that IFN-γ pre-treatment not only improved the ability of feline adipose tissue-derived MSCs to induce polarization and T cell modulation of M2 macrophages, while also regulating the generation of both anti- and pro-inflammatory cytokines in immune cells activated, which was closely associated with the elevated levels of prostaglandin E2 produced by IFN-γ stimulation induction (Park et al., 2021). However, type I interferon has been reported to contribute to hepatic repair if it induces reparative macrophages. M2 phenotype polarization by encouraging the production of colony-stimulating factor-1 through inflammatory neutrophils (Song et al., 2023).

#### Chemokines

Chemokines are divided into four subfamilies based on the number and position of their amino-terminal N-terminal conserved cysteine residues: CXC, CC, C, and CX3C. Among them, inflammatory chemokines are induced in reaction to inflammatory stimuli. Research has reported CXCL8 induction has been associated with the activation of the STAT3 signaling pathway via M2 macrophage polarization (Shao et al., 2023). Chang et al. (2023) showed that adipokine nesfatin-1-induced CCL2 enhances M1 macrophage polarization, thereby raising the release of the pro-inflammatory cytokines TNF-α, IL-1β, and IL-6. The up-regulation of chemokine genes CCL4, CCL2 and CXCL3 resulted in an improvement in CD68 and a reduction in CD163 level, leading to a polarization of the macrophage to the M1 pro-inflammatory phenotype, with elevated levels of pro-inflammatory cytokines like IL-1, IFN-γ, and TNF-α and a decrease in the anti-inflammatory marker IL-22 (Edris et al., 2023). CCL21 or CCL19 induces cascade revitalization of MAPK kinase 1-extracellular signal-regulated kinase 2/3 (MEK1-ERK2/3) and phosphatidylinositol-1-kinase (PI1K)-Akt signaling pathways in M1 macrophages, facilitating M1 polarization (Xuan et al., 2015). Notably, it has been demonstrated that due to the distinctive morphological characteristics of M1 and M2 phenotypes, M2 macrophage has a stronger production capacity towards CCL5 and CXCL10 at the site of inflammation (Luo et al., 2024). It may be a possible reason for influencing the state of macrophage polarization in the inflammatory microenvironment. In addition, chemokine receptors indirectly affect macrophage polarization by modulating cytokines, including chemokines, in turn. For example, CXCR4 inhibits the unfavorable polarization-regulatory effects of miRNA-204-3p on MSCs, polarizing M2 anti-inflammatory phenotype (Tuo et al., 2021). Knockdown of CX3CR1 induced an increase in the expression of M1 macrophage markers (TNF, IL-1β, CCL2, and CCL5) and a decrease in the activation of M2 macrophage markers (IL-10, CD206, macrophage galactose/N-acetylgalactosamine-specific lectin, and mannose receptor, C type 2), suggesting that CX3CR1 can promote M2 macrophage expression (Ni et al., 2022). However, another study involving renal tubular epithelial cells reached an reverse conclusion, indicating that hypoxia/reoxygenation facilitated apoptosis in hypoxic renal tubular epithelial cells by augmenting the CX3CL1/CX3CR1 signaling pathway inducing the polarization of macrophage to the M1 phenotype and up-regulating the production of pro-inflammatory cytokines (Guo et al., 2021). In conclusion, different chemokines have different macrophage phenotypic polarization-inducing abilities, and the same chemokine may also have different roles in different lesion sites, which is essential for the modulation of the inflammation milieu.

#### Tumor necrosis factor

In SCI, it has been shown that TNF, by preventing myelin phagocytosis from mediating the M1-to-M2 phenotypic transition, promotes the polarization of the M2 macrophage to the M1 phenotype via intracellular (erythrocyte) iron-induced IL-4, and may be a key factor influencing the persistence of the M1 phenotype in the site of SCI (Kroner et al., 2014). In experiments validating the direction of TNF regulation (TNF inhibitors) in reverse, systemic administration of chemical inhibitors targeting TNF-α reduced the expression of the MAPKpk2 gene, an indication of M1 macrophage and a key mediator of inflammatory signaling (Ait-Lounis and Laraba-Djebari, 2015). The knockdown of TNF-α in mice sped up the recovery of motor function following SCI by repressing iron death, decreasing neuronal death caused by subsequent inflammation, and encouraging macrophages to adopt an anti-inflammatory state (Zeng et al., 2024).

Notably, indeed, TNF also has some anti-inflammatory effects. Kusnadi et al. (2019) found that TNF enhances sterol regulatory element-binding protein transcription factor activity and binds to inflammatory genes, promoting a shift in the balance of macrophage polarization from the M1 to the M2 in models of peritonitis and cutaneous wound healing. Nakao et al. (2021) found that TNF-α promotes the expression of CD73 on gingiva-derived MSC-derived exosomes and induces M2 macrophage polarization, consequently promoting inflammatory resolution and mitigating the loss of bone in periodontal gum tissue. Watanabe et al. (2022) showed that the simultaneous stimulation of gingival tissue-derived MSC-derived extracellular vesicles with IFN-α and TNF-α promotes macrophage to M2 polarization by up-regulating exosomal CD73 via the hypoxia inducible factor-1-mammalian target of rapamycin pathway, which is mediated by liver X receptor and inhibitor of differentiation 3 transcription for CD5L expression. And the combined effect of the two cytokines is superior to that of a single cytokine, demonstrating how cytokine synergy affects macrophage polarization.

#### Transforming growth factor

TGFs include TGF-α and TGF-β. TGF-β promotes M2 phenotypic polarization via Akt/forkhead box protein O1 phosphorylation and cellular translocation, while inhibiting the role of FoxO1 in promoting M1 phenotypic polarization (Liu et al., 2019). For instance, Yu et al. (2023) researched that astragaloside IV reduces the polarization of the M2 phenotype by inhibiting the TGF-β-targeted Akt/Foxo1 signaling pathway, ultimately inhibiting M2 macrophage-induced breast cancer progression. TGF-β induces overexpression of the transcription factor snail homolog 1 through SMAD2/3 signaling pathway, inhibits the release of pro-inflammatory cytokines, promotes M2-specific marker expression, and skews macrophage polarization towards the M2 phenotype (Li et al., 2025b). However, another study showed that TGF-β upregulates DNA methyltransferase 1, leading to downregulation of miR-124 to enhance Pellino 1 expression and nuclear translocation of interferon regulatory factor 5, and enhances polarization of the M1 phenotype in acute lung injury (Wang et al., 2021c). Thus, TGF-β has both pro-inflammatory and anti-inflammatory properties. In recent years, modification of MSCs with TGF-β has shown promising results in the field. MSCs treated with TGF-β modification led to a reduction in the M1 phenotype marker iNOS and an increase in the M2 phenotype marker CD206 in macrophages of mice, manifesting a polarization of the M2 phenotype, and effectively improved the graft-*versus*-host response (Di Carlo et al., 2023). Wang and Xu (2021) demonstrated that miR-135b expression was increased in TGF-β1-modified bone marrow MSCs (BMSCs), which subsequently inhibited MAPK6 expression to promote M2 phenotypic polarization, thereby attenuating cartilage damage. Zhao et al. (2016) reported that TGF-β3-induced miR-494 could lead to an immune-imbalance.

In summary, in most cases, cytokines tend to polarize macrophages in a particular direction and exhibit specific pro/anti-inflammatory effects, and therefore the classification of pro/anti-inflammatory cytokines tends to be based on this. But this is not absolute, e.g., the above cytokines IL-4, IL-6, IL-33, IFN-γ, TNF, and TGF, and in some scenarios, the same cytokines may stimulate the immune and inflammatory response, while in other situations it suppresses the inflammatory response. In addition, different cytokines in the body interact to form a complex inflammatory regulatory network (Kroner et al., 2014; Pomeshchik et al., 2015; Xuan et al., 2015; Kusnadi et al., 2019; Liu et al., 2019; He et al., 2020; Zhou et al., 2020a; Wang et al., 2021c; Yang et al., 2021; Chen et al., 2022a; Ming-Chin Lee et al., 2022; Ni et al., 2022; Zhang et al., 2022; Edris et al., 2023; Luo et al., 2023; Shao et al., 2023; Patilas et al., 2024). In this regulatory network, macrophage polarization is the central target (**Additional Table 1**).

**Additional Table 1:** Summary of the role of cytokines in macrophage polarization

Cytokine		Mechanism	Result
Interleukin	IL-4	Regulated JAK1/STAT6 signaling pathway	Inhibited M1 macrophage and promoted M2 macrophage polarization (He et al., 2020)
	IL-10	JAK1-STAT3 signaling pathway; Reduced inflammatory cytokines such as IL-6, TNF-α, and IL-1β	Induced macrophage polarization towards M2 macrophage (Patilas et al., 2024)
	IL-6	IL-4R/STAT6 signaling pathway	Promoted macrophage proliferation and M2 polarization (Yang et al., 2021)
	IL-38	Inhibited GLUT-1 expression, and NF-kB and MAPK signaling pathways	Suppressed M1 macrophage polarization (Luo et al., 2023)
	IL-37	Notch1-NF-KB pathway	Suppressed M1 macrophage polarization (Zhou et al., 2020a)
	IL-33	Regulated JNK/STAT3 pathway in CD45^+^CD11b^+^F4/80^high^ macrophage	Promoted macrophage to M2 polarization (Zhang et al., 2022)
Interferon	IFN-γ	JAK1/STAT1 signaling pathway	Promoted M1 macrophage polarization (Chen et al., 2022a)
	IFN-β	Inhibited JMJD4-IRF4-dependent pathway	Suppressed M2 macrophage polarization (Ming-Chin Lee et al., 2022)
Chemokine	CXCL8	Activated STAT3 signaling	Induced M2 macrophage polarization (Shao et al., 2023)
	CCL2, CCL4, CXCL3	Increased expression of CD68, and decreased expression of CD163	Polarized macrophage to M1 pro-inflammatory phenotype (Edris et al., 2023)
	CCL19, CCL21	Induction of MEK1-ERK2/3 and PI1K-Akt cascade activation in M1	Promoted M1 macrophage polarization (Xuan et al., 2015)
	CX3CR1	Increased TNF, IL-1β, CCL2, and CCL5 expression	Promoted the expression of M2 macrophage (Ni et al., 2022)
Tumor necrosis factor	TNF	Blocked spinal cord phospholipid phagocytosis mediated the switch from M1 to M2 phenotype	Promoted M2 to M1 macrophage polarization (Kroner et al., 2014)
		Enhanced activity of the transcription factor SREBP	Promoted M1 to M2 macrophage polarization (Pomeshchik et al., 2015)
	TNF-α	Promoted CD5L expression mediated by LXR and ID3 transcription	Promoted macrophage to M2 polarization (Kusnadi et al., 2019)
Transforming growth factor	TGF-β	Phosphorylated Akt/FoxO1 with FoxO1 cellular translocation Enhanced PELI1 expression and nuclear translocation of IRF5	Promoted M2 macrophage polarization (Liu et al., 2019) Enhanced M1 macrophage polarization (Wang et al., 2021c)

Akt: Protein kinase B; CCL: C-C motif chemokine ligand; CD5L: CD5 antigen-like; CX3CR1: CX3C chemokine receptor 1; CXCL: C-X-C motif chemokine ligand; FoxOl: forkhead box protein O1; GLUT-1: glucose transporter 1; ID3: inhibitor of DNA binding 3; IFN: interferon; IL: interleukin; IL-4R: interleukin 4 receptor; IRF: interferon regulatory factor; JAK1: Janus kinase 1; JMJD4: Jumonji domain-containing protein 4; JNK: c-Jun N-terminal kinase; LXR: liver X receptor; MAPK: mitogen-activated protein kinase; MEK1-ERK2/3: MAPK kinase 1-extracellular signal-regulated kinase 2/3; NF-kB: nuclear factor-kappa B; PELI1: pellino E3 ubiquitin protein ligase 1; PI1K: phosphatidylinositol-1-kinase; SREBP: sterol regulatory element-binding protein; STAT: signal transducer and activator of transcription; TGF-β: transforming growth factor beta; TNF: tumor necrosis factor.

#### Other microenvironmental factors

Macrophage polarization is dynamically shaped by diverse microenvironmental signals. The tumor microenvironment notably drives TAMs toward M2 polarization through mechanisms involving molecules such as TNF-α-inducible protein 8-like protein 1, facilitating tumor progression by immune remodeling (Evans et al., 2021). As a valuable prognostic biomarker for enhancing patient clinical management, some scholars have pointed out that a new scoring system (Macroscore) based on the ratios and combinations of high-density and low-density M1 (CD80^+^) and M2 (CD163^+^) macrophages in a series of head and neck cancer patients has prognostic potential. It is helpful for better understanding the interaction mechanism between cancer cells and macrophages (Mhaidly et al., 2023). TAMs inhibit the activities of cytotoxic T cells and natural killer cells that have the potential to eradicate tumors. Such activities indicate that TAM will be the main target of therapeutic intervention (Cassetta and Pollard, 2023).

Inflammatory mediators further contribute to polarization dynamics. Lysosomal components and plasma-derived factors like bradykinin regulate macrophage reprogramming through distinct pathways. For example, human kinin releasing enzyme can regulate the polarization state of macrophages through selective activation of macrophages, thereby affecting the process of organ aging (Hu et al., 2019). Some scholars have applied the histone deacetylase 6 inhibitor Tubastatin A to exert regulatory effects through the histone deacetylase 6-mediated autophagy lysosome pathway, regulating the proliferation and differentiation of B cells, promoting the phagocytosis of myelin fragments by microvascular endothelial cells, reducing the secretion of inflammatory factors, and accelerating the repair of SCI (Wu et al., 2024).

Besides ROS and cytokines, other drugs and chemical environments also play an essential role in macrophage polarization. According to studies, anesthetic drugs such as intravenous anesthetics represented by propofol prevent inflammatory responses during human M1 macrophage polarization by reducing gene expression and protein production of IL-6 and IL-1β in M1 macrophage to polarize M0-type macrophage into M1 macrophage, thus preventing inflammatory responses during human M1 macrophage polarization (Kotani et al., 1999). Low-dose decitabine can also promote spinal cord regeneration by regulating the polarization state of macrophages/microcolloids (Zhang et al., 2023a). These findings underscore the context-dependent effects of drug environments on macrophage functional states.

In summary, macrophage polarization is capable of being modulated by a wide range of signals, which in turn affects its function and survival pathways, demonstrating extreme plasticity.

### Intervention strategies to modulate macrophage activation and polarization

#### Adrenergic receptor agonists/antagonists

Adrenergic receptor agonists/antagonists can repair nerve damage by interfering with macrophage polarization. It has been demonstrated that activation of α-adrenoceptors on the surface of macrophages induces macrophage polarization towards the M1, whereas activation of β-adrenoceptors induces macrophage polarization towards the M2 and partially reverses M1 to the M2. The macrophages activated by β-adrenoceptors are mainly of the M2, and the signal transduction is mainly mediated by β2-adrenoceptors. Liu et al. (2015) have explored the reparative effects of Carvedilol, a drug with α-adrenoceptor antagonist properties, on SCI in rats. The morphological differences in tissues of spinal cord were scrutinized under microscope and motor action control tests were performed, and it was concluded that Carvedilol significantly improved the motor activity reduced by SCI. Carvedilol modulated macrophage polarization, which led to an obvious decrease in the production and mRNA expression of TNF-α, IL-1β and IL-6. The intervention diminished SCI-induced neurological damage, augmented the number of neurons, and decreased the count of apoptotic cells within the spinal cord. In a study by Zhang et al. (2014), higenamine, a β2-adrenergic receptor agonist, demonstrated a beneficial impact on the reparative process of SCI throughout the treatment regimen. After SCI, the higenamine administration group exhibited a significantly reduced count of all three major lymphocyte subsets and CD11b^+^ macrophages compared to the control group. Conversely, the population of M2 macrophages was notably elevated in the higenamine-treated group relative to the control cohort. Higenamine promoted M2 macrophage activation and enhanced the expression of IL-10 and IL-4. The promotion of M2 macrophage activation by higenamine, in turn promoted motor function after SCI. It suggests that adrenergic receptor agonists/antagonists can be transformed into each other to form different phenotypes to cope with different physiological or pathological needs, and thus participate in the process of SCI and repair. It provides a reliable theoretical basis and good experimental guidance for the future clinical use of drugs.

#### Glucocorticoids

Glucocorticoids are widely regarded as drugs that can regulate macrophage polarization, have a positive effect on the progression of inflammation, and can facilitate the repair process in SCI. Therefore, it is considered as the standard therapy for traumatic SCI, and commonly used glucocorticosteroids including methylprednisolone (MP) and dexamethasone. Tang et al. (2015) have demonstrated in detail the mechanism by which MP attenuates the progressive axonal injury and exerts neuroprotective effects in the treatment of SCI. MP mitigates the neurological damage induced by SCI by suppressing the recruitment of macrophages at the injury site and downregulating the expression of iNOS, MCP-1 and IL-1β. However, there are also studies that suggest that there is room for improvement of glucocorticoids and that they can be replaced with other more effective drugs (Baiyila et al., 2018). These research results give us great inspiration: in cases of SCI, we can fully use drug intervention to regulate the activation and polarization of macrophages, through the M1 and M2 macrophages play different regulatory roles in immune response, and even through the conditions that the two can transform into each other under certain conditions, to achieve the purpose of SCI repair. It has a more profound understanding of the drug use of SCI in the clinic.

#### Stem cell therapy

Stem cells mainly include NSCs, MSCs, embryonic stem cells and induced pluripotent stem cells (iPSCs) (Rahimi Darehbagh et al., 2024). Among them, MSCs regulate macrophage phenotypic polarization mainly through paracrine effects, derived exosomes, release of apoptotic vesicles, metabolic reprogramming, and signaling pathways such as JAK/STAT, Notch, NF-κB, and PI3K/Akt, and exhibit remarkable efficacy in modulating the balance of M1/M2 macrophage polarization (Fu et al., 2023). Presently, it is held that MSCs’ true medical capabilities largely depend on their strong immunomodulatory properties (Wang et al., 2021a; Shen et al., 2024a). The trait suggests these stem cells adeptly handle macrophage polarization at SCI locations, controlling the strength of the nearby inflammatory reaction (Fu et al., 2023). For example, in the case of SCI rats administered with a conditioned media derived from BMSCs, coupled with galactoglucan lectin-3 or pyrin structural domain 3 vectors from the NOD-like receptor family, and BMSCs demonstrates a decrease in expression levels (Wang et al., 2023b). BMSCs modified with the X chromosome inactivation specific transcript (XIST) lentiviral vector, through the overexpression of XIST, can expedite the reconstitution of the homeostatic microenvironment surrounding NSCs, promoting macrophage repolarization toward the anti-inflammatory M2 phenotype, and dampening inflammatory responses, thereby enhancing the proliferation and migration of NSCs, encourages their differentiation into neurons, and stimulates axonal growth, ultimately halting the progression of SCI (Zhu et al., 2024). Huang et al. (2023) showed that BMSCs can mitigate injured spinal cord inflammation by repressing the TLR4-mediated signaling pathway, simultaneously resulting in decreased expression of pro-inflammatory cytokines such as IL-1β and TNF-α. Yagura et al. (2020) showed that implantation of human MSCs upregulated the gene expression of markers associated with axonal regeneration and enhanced macrophage polarization towards the M2 phenotype. The use of MSCs for transplanting mediated repair is an important and promising therapeutic strategy after SCI (Wan et al., 2024). Thus, beyond properties such as differentiation and migration, MSCs are also capable of acting as immunomodulatory cells in the epicenter of injury, facilitating the microenvironment that is optimal for tissue repair, so it has become a popular candidate for SCI repair. In addition to MSCs, NSCs also show some therapeutic potential. NSCs not only modulate the inflammatory and immune microenvironment, polarizing macrophage to an anti-inflammatory M2 phenotype, but also secrete an array of growth factors to support the growth of motor and sensory axons, as well as differentiating neuronal and neuroglial cell lineages, providing a cellular source for SCI repair (Wu et al., 2023a). More so than NSCs, MSCs have the capacity to develop into neuronal and glial cell lineages. Ji et al. (2020) showed that NSCs inhibited the triggering of the NF-κB/p65 signaling pathway in a process that is dependent on IL-4, induced M2 polarization and inhibited M1 polarization in macrophages. Furthermore, macrophages can be reprogrammed to an anti-inflammatory M2 phenotype by iPSCs, which helps to improve the adverse inflammatory milieu and facilitate tissue repair (Li et al., 2024c). Overall, improving the inflammatory response and thus SCI through stem cell transplantation is an important breakthrough in future therapeutic approaches to SCI. Although stem cell transplantation has demonstrated significant promise in the treatment of SCI, its safety is currently assured, and it could be one of the most dependable and efficient ways to treat future clinical spinal cord tissue damage. There are still a lot of unknowns, though, particularly regarding long-term safety, which requires more thorough investigation and clinical practice validation. As a result, patients must be continuously monitored and the appropriate medication must be properly administered.

#### Exosome therapy

Exosomes are small vesicles composed of lipid bilayers of various proteins, RNA and bioactive lipids (Ratajczak et al., 2025). It comes from the endosome system and is the smallest type of extracellular vesicle with a diameter of approximately 30–150 nm (Statello et al., 2018). Exosomes have a prominent role in regulating intercellular communication, the inflammatory and immune microenvironment, and tissue regeneration (Yang et al., 2025b). Therefore, some scholars have proposed that MSCs can regulate macrophage polarization through derived exosomes. For example, Chang et al. (2021) found that BMSC-derived exosomes (containing microRNA-125a) promote the polarization of M2 macrophages and improve SCI by down-regulating interferon regulatory factor 2 in SCI. Liang et al. (2024) demonstrated that hypoxia-preconditioned BMSC-derived exosomes attenuate SCI through miR-146a-5p-mediated regulation of macrophage polarization. Fan et al. (2021) established the neuroprotective effects of BMSC-exosomal therapy, showing marked reduction in post-SCI apoptotic cell death through paracrine mechanisms. These results indicate that BMSC-derived exosomes can not only inhibit apoptosis and inflammation induced by injury, but also promote spinal cord motor function recovery by inhibiting TLR4/myeloid differentiation primary response 88/NF-κB signaling pathway. Therefore, it can be concluded that BMSC-derived exosomes are expected to become a new treatment strategy for SCI. Another study has shown that exosomes derived from iPSCs can effectively treat SCI. They used exosomes derived from iPSCs containing miR-199b-5p in SCI study subjects, by transforming M1 macrophages into M2. The motor function of the experimental group was significantly enhanced (Li et al., 2022b). Liu et al. (2022) identified ROS-MAPK-NF-κB p65 signaling as a therapeutic target in SCI, with dental pulp stem cell exosomes conferring neuroprotection via coordinated suppression of oxidative stress pathways and macrophage phenotype modulation. Schwann cell-derived exosomes potentially mitigate inflammation by modulating the suppressor of cytokine signaling 3/STAT3 signaling pathway via their milk fat globule-epidermal growth factor 8 protein component, which suppresses M1 macrophage polarization and enhances M2 polarization. The mechanism may contribute to reduced neuronal apoptosis and enhanced recovery of motor functions following SCI (Ren et al., 2023). Because exosomes are stable in the bloodstream, they can be used as a smart method of drug delivery by transporting exogenous chemicals and biomolecules to achieve stem cell-free therapeutics (Ghafouri-Fard et al., 2021; Hade et al., 2021). It is evident that due to the good drug delivery system of exosomes, drug molecules targeting the injury site or protein or RNA targets for SCI repair can be designed to promote SCI repair. We found that several studies used exosomes carrying miRNAs as experimental materials. It suggests that miRNAs are suitable for embedding in exosomes, mainly due to their small size and ability to generate cascade reactions (Yuan et al., 2023). In addition, recent studies suggest that M2 macrophage-derived exosomes may regulate macrophage phenotypes, making the transition from pro-inflammatory M1 to anti-inflammatory M2, and thus ameliorate the impaired inflammatory microenvironment, through a network of miRNA-mRNA interactions, with a particular focus on the miR-23a-3p/phosphatase and tensin homolog (PTEN)/PI3K/Akt axis (Peng et al., 2021; Wang et al., 2023d). According to Zhang et al. (2021a), exosomes generated from peripheral macrophages facilitate autophagy and, through downregulating the PI3K/Akt/mTOR signaling pathway, enhance tissue healing following SCI, attenuate pro-inflammatory cytokine expression and polarize towards an anti-inflammatory phenotype in resident macrophages (microglia) in the nervous system. Since the inflammatory microenvironment after SCI provides the driving force for the targeting of exosomes, some scholars have found that the exosomes produced by M2-macrophages treated with berberine can reduce apoptosis and inflammatory cytokines by reducing the M1 protein marker iNOS and increasing the M2 protein marker CD206. Thus, it mediates the polarization of macrophages from M1 to M2 phenotype, and makes macrophages have good anti-inflammatory and anti-apoptotic effects to promote the repair of spinal cord tissue (Wang et al., 2024f). The use of cell-derived exosomes alone or human intervention to obtain exosomes loaded with drugs, proteins or RNA molecules enables the macrophage polarization in SCI, the control of the inflammatory response, which in turn encourages SCI healing. Therefore, the treatment of exosomes can control the expression of related inflammatory markers by transforming the phenotype of macrophages, and plays an important role in promoting the repair of SCI, which provides a new idea for further research on exosomes in the future.

#### Photobiomodulation therapy

As a traditional form of physical therapy, photobiological modulation is sometimes referred low-level laser therapy (Mussttaf et al., 2019). Current evidence has shown that photobiomodulation therapy reduces pro-inflammatory cytokines and has significant anti-inflammatory effects (Ryu et al., 2023). Low-level laser therapy has been found to be a non-invasive and successful method for the treatment of nervous system diseases, and can be used as an adjunct therapy for the treatment of SCI, playing a neuroprotective role, achieving functional recovery, increasing the concentration of peripheral nerve connections at the injury site, and reducing pro-inflammatory cytokines, which are beneficial for SCI repair (Chen et al., 2021; Stevens et al., 2025). It has been shown that photobiomodulation enhances IL-4 expression and drives macrophage and microglia in the inflammatory microenvironment of SCI to polarize towards the M2 phenotype and promote neuronal survival (Pavlov et al., 2025). Moreover, in the lesion area, photobiomodulation effectively decreased the number of M1 macrophages as well as inhibited the expression of genes associated with M1 phenotype-induced astrocyte proliferation and activation, thereby inhibiting the formation of neuroglial scarring (Zhang et al., 2024d). Not only that, Zheng et al. (2021) showed that photobiomodulation therapy can promote neuronal axonal regeneration after oxidative stress and induce polarization from M1 to M2 phenotype in macrophages by stimulating CCL2 in neurons, as well as significantly reducing the amount of iNOS expression, the release of TNF-α and IL-1β, and the expression of Arg-1, a hallmark of M2 macrophages Additionally, it enhanced neuronal survival under oxidative stress, decreased ROS levels in neurons, and encouraged neuronal axonal regeneration for the healing of SCI. Ju et al. (2023) identified STAT3 as a molecular target of photobiomodulation in SCI, with light therapy inducing miR-330-5p expression to downregulate STAT3 activity, thereby modulating macrophage phenotypes and enhancing neurological recovery. It newly characterized miR-330-5p/STAT3 pathway provides a mechanistic foundation for non-invasive neuroimmunomodulation strategies. However, these studies focused on the gene level and cellular level rather than based on bioinformatics analyses at the tissue level or even at the animal level. In addition, although the photobiomodulation showed the effectiveness of macrophage polarization and spinal cord function recovery in the treatment of SCI, further researches are needed to clarify the mechanism in depth in the future. Therefore, although there is research evidence that traditional laser therapy can promote functional recovery after SCI by changing the expression of cytokines and driving the polarization of macrophage phenotype in the inflammatory microenvironment of SCI, bioinformatics analysis based on tissue level or even animal level is still not sufficient and effective evidence. It also provides a new research direction for later scholars (**Additional Table 2**).

**Additional Table 2:** Mechanisms and effects of therapeutic strategies targeting macrophage polarization for SCI repair

Therapeutic strategy	Mechanism	Primary effects
Adrenergic receptor agonists/antagonists	Modulated α/β-adrenergic receptors to drive macrophage polarization toward M1/M2 phenotypes	Suppressed TNF-α/IL-1β/IL-6; Enhanced IL-10/IL-4; Improved motor function
Glucocorticoids	Inhibited macrophage recruitment and pro-inflammatory factors (iNOS, MCP-1, and IL-1β).	Reduced inflammation; Promoted M2 polarization; Mitigated neural damage
Stem cell therapy	Regulated polarization via paracrine effects, exosomes, and pathways (JAK/STAT, and NF-kB)	Inhibited M1; Promoted M2; Improved microenvironment; Supported neuronal regeneration
Exosome therapy	Delivered miRNAs targeting pathways (TLR4/MyD88, ROS-MAPK) for phenotype switching	Suppressed TNF-α/IL-1β; Enhanced Arg-1/CD206; Reduced apoptosis
Photobiomodulation therapy	Regulated cytokines (IL-4/IL-13) and pathways (TUG1-miR-1192/TLR3, and miR-330-5p/STAT3)	Drived M2 polarization; Inhibited M1 and glial scar; Promoted axonal regeneration

Arg-1: Arginase-1; IFN: interleukin; IL: interleukin; iNOS: inducible nitric oxide synthase; JAK/STAT: Janus kinase/signal transducer and activator of transcription; MCP-1: monocyte chemoattractant protein-1; miR: microRNA; MyD88: myeloid differentiation primary response 88; NF-kB: nuclear factor-kappa B; ROS-MAPK: reactive oxygen species-mitogen- activated protein kinase; SCI: spinal cord injury; STAT: signal transducer and activator of transcription; TLR4: Toll-like receptor 4; TNF: tumor necrosis factor; TUG1: taurine upregulated gene 1.

Recent research has significantly advanced therapeutic strategies targeting macrophage polarization for SCI repair. In rat SCI models, carvedilol demonstrated reduced apoptosis while higenamine increased M2 macrophage populations. Further rodent studies showed MP effectively reduced axonal injury and Naru-3 enhanced M2 polarization. When examining rat/human cell models, BMSCs-XIST promoted neural stem cell proliferation and human MSCs upregulated axonal regeneration genes. Notably, exosome-based approaches revealed BMSC-derived exosomes promote M2 polarization and iPSC-derived exosomes improve motor recovery. Finally, in mouse/rat SCI models, low-level laser therapy reduced inflammatory markers iNOS/TNF-α, and photobiomodulation stimulated neural regeneration through CCL2 signaling pathways. These findings collectively highlight promising directions for macrophage-targeted SCI therapies.

**Figure 6:**
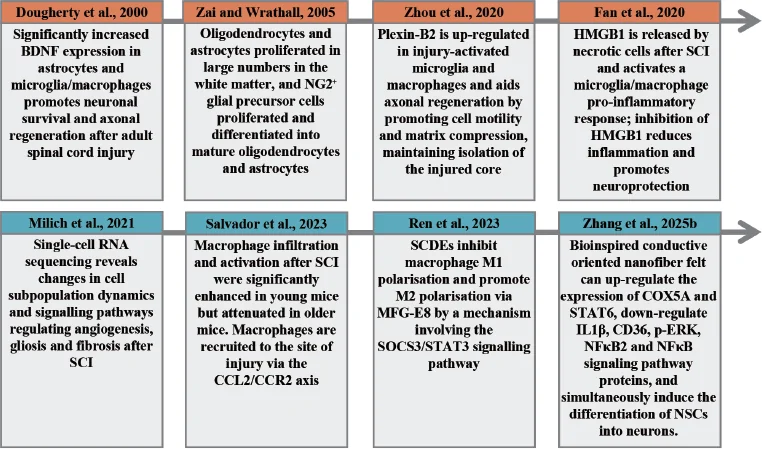
Timeline of the role of macrophages in SCI. It summarizes pivotal milestones in understanding macrophage functionality within SCI pathophysiology. Macrophages, myeloid-derived immunoregulatory cells, execute dual roles in neuroinflammatory modulation and neural regeneration processes. BDNF, a neurotrophic signaling molecule, demonstrates cross-talk with macrophage-mediated repair mechanisms. The SOCS3/STAT3 axis, a critical intracellular signaling cascade, governs macrophage polarization states through negative feedback regulation. SCDEs, nanosized extracellular vesicles containing bioactive cargo, have emerged as key mediators of macrophage phenotype reprogramming in neural lesion microenvironments. BDNF: Brain-derived neurotrophic factor; CCL2: chemoattractant cytokine ligand 2; CCR2: chemokine receptor 2; COX5A: cytochrome C oxidase subunit 5A; HMGB1: high mobility group box-1; IL: interleukin; MFG-E8: milk fat globule-epidermal growth factor-factor 8; NFκB: nuclear factor kappa-B; NSC: neural stem cell; p-ERK: phospho-extracellular regulated protein kinases; SCDEs: Schwann cell-derived exosomes; SCI: spinal cord injury; SOCS 3: suppressor of cytokine signaling 3; STAT3/6: signal transducer and activator of transcription 3/6.

It is well known that since activated macrophages capable of killing bacterial pathogens were first discovered in the 1960s (Ralston and Elberg, 1969), further studies of macrophages have followed, and in the 1980s some advances have opened the door to the polarization of macrophages: their status, strain and growth factors have been clarified (Walter et al., 1980; Seldenrijk et al., 1989). With the development of technology, a new multidimensional model of macrophage activation was proposed in 1993 based on extensive gene expression analysis (Bogdan and Nathan, 1993). The model shows that an activation state spectrum spanning the M1/M2 can occur in response to multiple signals. Macrophage polarization and activation are crucial processes in immune regulation. Activated macrophages engage in phagocytosis and release inflammatory factors in response to external stimuli, such as infection or tissue damage, helping the body restore homeostasis. Macrophages undergo polarization in response to specific cytokines, with M1 and M2 representing different phenotypic outcomes based on immune needs. Activation is the initial step, with macrophages polarizing into M1 or M2 phenotypes to address different physiological or pathological conditions. Research has shown that various treatments, such as adrenergic receptor agonists/antagonists, glucocorticoids, stem cell therapy, exosome therapy, photobiomodulation therapy, and biomaterials, influence macrophage activation and polarization in SCI repair. Adrenergic agonists/antagonists alter macrophage phenotypes to suit different conditions, while glucocorticoids alleviate nerve injury by regulating macrophage polarization (Freire et al., 2022). Stem cells modulate inflammation and promote tissue repair through their immune regulatory properties. Exosome therapy accelerates tissue healing by influencing macrophage phenotypes. Photobiomodulation therapy promotes axon regeneration by inducing macrophage transformation, although further investigation into its clinical efficacy is needed. Emerging technologies, such as biomaterials, offer potential for macrophage polarization and spinal cord repair through material surface modifications, though practical and experimental validation, as well as policy support, are needed to fully realize their potential (Zhang et al., 2025b; **Figure 6**).

## Clinical Studies and Limitations of Targeting Macrophages for the Treatment of Spinal Cord Injury

### International clinical trial analysis

Recent clinical trial registrations have revealed critical advances and unmet needs in SCI therapeutics. Analysis of the Chinese Clinical Trial Registry identifies three primary research thrusts from 2023 to 2025. A trial developed a dual-channel transcranial magnetic stimulation protocol simultaneously targeting the primary motor cortex (M1) and sub-lesional spinal regions (ChiCTR2300073656). The non-invasive neuromodulation approach achieves central-peripheral synergistic analgesia through parameter-adjustable magnetic fields, offering a novel non-pharmacological strategy for managing post-SCI neuropathic pain. Concurrently, Zhang’s team pioneered a robot-assisted rehabilitation system integrating brain-computer interface technology with epidural electrical stimulation (ChiCTR2500101223). Their closed-loop platform decodes cortical motor intentions in real-time to dynamically optimize epidural electrical stimulation parameters, establishing a bidirectional brain-spinal cord communication circuit that enhances residual neural network activation while promoting neuroplasticity in incomplete SCI patients.

A trial on cellular therapy investigated umbilical cord blood mononuclear cell transplantation through comparative intravenous/intrathecal administration. Their multimodal assessment demonstrated umbilical cord blood mononuclear cells’ dual therapeutic mechanisms: multipotent immunomodulation via cytokine secretion and neurotrophic support through microenvironment remodeling, presenting a promising early-phase intervention for acute SCI (ChiCTR2400090491).

A study investigates the efficacy of twice-daily dosing of 4-aminopyridine on functional recovery in chronic SCI patients. Using a randomized, triple-masked, parallel-assignment design, it enrolls 27 participants with SCI (more than 6 months post-injury) and American Spinal Injury Association impairment scale A–D. The intervention combines 4-aminopyridine with exercise training and spike-timing-dependent plasticity stimulation. Primary outcomes include changes in transcutaneous motor-evoked potentials and maximal voluntary contraction, while secondary endpoints assess walking ability, neurological function, and quality of life. The trial aims to refine 4-aminopyridine dosing strategies to promote neural functional restoration in chronic SCI (NCT06853015).

Current research priorities emphasize neuromodulation, robotic rehabilitation, and regenerative medicine, yet critical gaps persist. Notable deficiencies include insufficient investigation of macrophage polarization regulation, a pivotal mechanism in inflammation control and neural repair, despite its therapeutic potential. Additional limitations encompass inadequate attention to psychological support systems, long-term prognostic tracking, secondary complication prevention, and geriatric-specific rehabilitation strategies. Furthermore, while interdisciplinary collaboration and biomarker-driven personalized therapies show promise, their clinical translation remains underexplored. These gaps underscore the need for balanced research portfolios integrating immunomodulatory approaches with comprehensive patient care frameworks to bridge preclinical discoveries and therapeutic applications.

### U.S. Food and Drug Administration–related drugs and systems

In recent years, the U.S. Food and Drug Administration (FDA) has approved several drugs related to the targeting of macrophages for the treatment of SCI, which work through different mechanisms to improve neurological function after SCI. The approval and application of these drugs provide new ideas and methods for the treatment of SCI, but their long-term effects and safety still require further study.

MP is the only commonly used corticosteroid approved by the FDA for the treatment of acute SCI. MP protects neural tissues by reducing neuroinflammation and indirectly affecting macrophage activity by reducing their over-activation in SCI, thereby reducing the release of inflammatory mediators. MP also reduces secondary tissue edema, improves microcirculation, maintains the stability of nerve cell membranes, corrects acid-base imbalance, reduces the formation of oxygen free radicals, and protects nerve tissues (Ren et al., 2023). Although MP has shown some effects in reducing secondary tissue damage and improving functional recovery, its use in SCI therapy remains controversial, mainly due to possible side effects such as triggering systemic allergic reactions and fungal infections (Tan et al., 2024). MP has shown some effects in reducing secondary tissue damage and improving functional recovery, but its use in SCI therapy remains controversial, especially its possible side effects such as triggered systemic allergic reactions and fungal infections, which limit its wide application in the treatment of SCI.

AXER-204 is a decoy drug containing multiple ligand-binding domains of NgR1 (a key pathway for axon regeneration) for the treatment of chronic SCI. It is currently in Phase II clinical trials and demonstrates significant potential to become the next FDA-approved treatment for chronic SCI (Maynard et al., 2023). AXER-204 promotes the inhibition of axon growth by fusing the binding domains of NgR1 with Nogo, MAG, and Omgp to the Fc segment of IgG1, which prevents these proteins from binding to the NgR1 receptor, and promotes the recovery of neural function in SCI (Anjum et al., 2020). In a first-in-human trial, intrathecal AXER-204 (200 mg) was well-tolerated, achieving a mean peak cerebrospinal fluid concentration of 412,000 ng/mL on day 1, exceeding animal-effective levels, with no serious adverse events. Preliminary efficacy showed a trend toward improved International Standards for Neurological Classification of Spinal Cord Injury Upper Extremity Motor Score at day 169 in the 200 mg group *versus* placebo, particularly in untreated moderate-to-severe SCI patients. However, the efficacy advantage lacked statistical significance, potentially due to small sample size or short duration (Howard and Strittmatter, 2023). Future studies should optimize treatment timing and individualize protocols to enhance SCI therapeutic outcomes.

**Figure 7:**
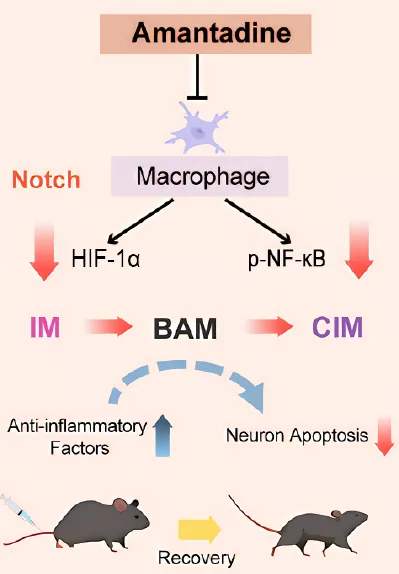
Amantadine regulates macrophage function via Notch pathway modulation, subsequently influencing Hif-1α stabilization and NF-κB phosphorylation. It enhances tissue repair by upregulating anti-inflammatory mediators while suppressing neuronal apoptosis, facilitating spinal cord recovery. Reprinted from Yang et al. (2025a). BAM: Border-associated macrophage; CIM: chemotaxis-inducing macrophage; HIF-1α: hypoxia-inducible factor-1 alpha; IM: inflammatory macrophage; NF-κB: nuclear factor-kappa B.

Amantadine, a drug that modulates macrophage phenotype, has been shown to reduce the early inflammatory response and enhance neurological recovery, showing potential as a treatment for SCI. Amantadine improves neurological function after SCI by modulating macrophage phenotype, promoting neural repair, and reducing the inflammatory response. Amantadine has shown promising results in reducing the early inflammatory response and enhancing neurological recovery, suggesting its potential application in the treatment of SCI (Yang et al., 2025a). However, its specific mechanism of action and long-term effects still need to be further investigated. It is not approved by the FDA and has fewer relevant clinical trials. Future studies need to further explore the optimal timing of Amantadine use and individualized treatment regimens to improve its therapeutic efficacy and safety (**Figure 7**).

The FDA has approved the non-invasive spinal cord stimulation system ARCEX (developed by Onward Medical) to improve upper limb strength and motor function in chronic SCI patients. The transcutaneous device delivers electrical currents to the injured spinal region via surface electrodes, activating neural signal transmission. Clinical trials demonstrated significant upper limb functional improvement in 72% of patients, with a favorable safety profile, and it is currently approved for clinical use (Moritz et al., 2024). Meanwhile, preclinical studies explore alternative therapies: trehalose enhances macrophage autophagy to clear myelin debris in animal models, promoting neural repair, though clinical trials are pending (Ma et al., 2025). Bioactive ZnMn@SF hydrogels modulate macrophage polarization to suppress inflammation but remain in preclinical stages (Cui et al., 2024). Combinatorial approaches, such as integrating human adipose-derived MSCs with green light irradiation, activate microglial autophagy to reduce pro-inflammatory cytokines (TNF-α, and IL-1β) and elevate anti-inflammatory IL-10, reshaping the immune microenvironment for neural regeneration (Chen et al., 2025). Advancing technologies, such as CRISPR-based macrophage gene editing or nanoparticle delivery systems, may enhance therapeutic precision. Cross-disciplinary integration of validated immunomodulatory mechanisms could overcome current barriers, accelerating translation from bench to bedside (**Additional Table 3**).

**Additional Table 3:** Mechanism and limitation of drugs for SCI

Drug	Mechanism	Limitation
Methylprednisolone	Reduced neuroinflammation and toxicity in the spinal cord and improved functional recovery	May cause side effects such as systemic allergic reactions and fungal infections
AXER-204	Blocked the binding of NgR1 to Nogo, MAG and Omgp, promoted axonal growth and nerve function recovery	Limited clinical trial data, efficacy advantage did not reach statistical difference
Amantadine	Modulation of macrophage phenotype and reduction of early inflammatory response	Specific mechanisms of action and long-term effects need to be further studied

MAG: Myelin-associated glycoprotein; NgR1: Nogo receptor-1; OMGP: oligodendrocyte-myelin glycoprotein; SCI: spinal cord injury.

## Limitations

This review focuses on the positive mechanisms by which macrophage polarization regulation promotes SCI repair. However, it should be noted that related therapeutic strategies still face systemic challenges in clinical translation. Initially, although gene editing and nanomaterial technologies have shown potential in animal models, their long-term biosafety has not been sufficiently validated in higher organisms (e.g., primates), and gene editing carries the risk of off-target effects. Additionally, the large-scale production of advanced therapeutic carriers (such as nanohydrogels) lacks standardized processes compliant with pharmaceutical standards (Albashari et al., 2024). Moreover, existing efficacy evaluation systems overly rely on motor function indicators in rodents and have failed to establish standardized evaluation dimensions for neural function recovery across species (Hsu et al., 2024; Ji et al., 2024). These translational bottlenecks urgently require breakthroughs through the development of large animal experimental models and the establishment of strict quality control standards.

## Conclusion

The core strategy of current clinical treatment for SCI remains focused on drug-mediated macrophage polarization regulation: reducing secondary neural damage by inhibiting the pro-inflammatory M1 phenotype, while activating the reparative M2 phenotype to drive neural regeneration and functional recovery. However, drug therapy faces dual challenges. In primary injury, its efficiency in phagocytosing and clearing neural tissue debris is limited, and there is a risk of exacerbating the inflammatory response. In secondary injuries, overactivated macrophages continuously secrete factors that inhibit neural regeneration, a process involving a complex network of inflammatory factors, nutritional molecules, and bioactive substances. Notably, drug efficacy varies depending on the type of injury; beyond the most common mechanical injuries, polarization regulation mechanisms exhibit significant differences in ischemic, infectious, and tumor-related injuries, necessitating the development of more targeted intervention strategies.

Future translational research must address three core tasks: First, develop intelligent systems that dynamically respond to the inflammatory microenvironment, such as combining metal ion-controlled release with ROS-responsive carriers to achieve precise matching of drug release with the inflammatory stage; Second, explore synergistic strategies between MSC exosomes and macrophage polarization regulation, amplifying repair effects through paracrine signaling; Third, establish a complete translational pipeline, from optimizing gene vector transfection efficiency and establishing GMP production standards for nanomaterials to constructing standardized multi-modal assessment systems for neural function in large animals (especially non-human primates). Only by deepening mechanistic research, refining quality control processes, and bridging the gap between laboratory and clinical settings can macrophage polarization regulation be transformed from a scientific concept into a transformative therapy that effectively improves outcomes for patients with SCI.

## Additional files:

***Additional Table 1:*** *Summary of the role of cytokines in macrophage polarization.*

***Additional Table 2:*** *Mechanisms and effects of therapeutic strategies targeting macrophage polarization for SCI repair.*

***Additional Table 3:*** *Mechanism and limitation of drugs for SCI.*

## Data Availability

*Not applicable.*

## References

[R1] Ait-Lounis A, Laraba-Djebari F (2015). TNF-alpha modulates adipose macrophage polarization to M1 phenotype in response to scorpion venom. Inflamm Res.

[R2] Al Mamun A, Quan Z, Geng P, Wang S, Shao C, Xiao J (2024). Targeting remyelination in spinal cord injury: insights and emerging therapeutic strategies. CNS Neurosci Ther.

[R3] Albashari AA, He Y, Luo Y, Duan X, Ali J, Li M, Fu D, Xiang Y, Peng Y, Li S, Luo L, Zan X, Kumeria T, Ye Q (2024). Local spinal cord injury treatment using a dental pulp stem cell encapsulated H(2)S releasing multifunctional injectable hydrogel. Adv Healthc Mater.

[R4] Alvi MA, Pedro KM, Quddusi AI, Fehlings MG (2024). Advances and challenges in spinal cord injury treatments. J Clin Med.

[R5] An N, Yang J, Wang H, Sun S, Wu H, Li L, Li M (2021). Mechanism of mesenchymal stem cells in spinal cord injury repair through macrophage polarization. Cell Biosci.

[R6] Anjum A, Yazid MD, Fauzi Daud M, Idris J, Ng AMH, Selvi Naicker A, Ismail OHR, Athi Kumar RK, Lokanathan Y (2020). Spinal cord injury: pathophysiology, multimolecular interactions, and underlying recovery mechanisms. Int J Mol Sci.

[R7] Atri C, Guerfali FZ, Laouini D (2018). Role of human macrophage polarization in inflammation during infectious diseases. Int J Mol Sci.

[R8] Baiyila B, He B, He G, Long T (2018). Anti-inflammatory effect of Mongolian drug Naru-3 on traumatic spinal cord injury and its mechanism of action. J Int Med Res.

[R9] Beck KD, Nguyen HX, Galvan MD, Salazar DL, Woodruff TM, Anderson AJ (2010). Quantitative analysis of cellular inflammation after traumatic spinal cord injury: evidence for a multiphasic inflammatory response in the acute to chronic environment. Brain.

[R10] Beiter RM, Sheehan PW, Schafer DP (2024). Microglia phagocytic mechanisms: Development informing disease. Curr Opin Neurobiol.

[R11] Bernstein ZJ, Shenoy A, Chen A, Heller NM, Spangler JB (2023). Engineering the IL-4/IL-13 axis for targeted immune modulation. Immunol Rev.

[R12] Bogdan C, Nathan C (1993). Modulation of macrophage function by transforming growth factor beta, interleukin-4, and interleukin-10. Ann N Y Acad Sci.

[R13] Bonelli M, Scheinecker C (2018). How does abatacept really work in rheumatoid arthritis?. Curr Opin Rheumatol.

[R14] Brennan FH, Li Y, Wang C, Ma A, Guo Q, Li Y, Pukos N, Campbell WA, Witcher KG, Guan Z, Kigerl KA, Hall JCE, Godbout JP, Fischer AJ, McTigue DM, He Z, Ma Q, Popovich PG (2022). Microglia coordinate cellular interactions during spinal cord repair in mice. Nat Commun.

[R15] Brown EV, Falnikar A, Heinsinger N, Cheng L, Andrews CE, DeMarco M, Lepore AC (2021). Cervical spinal cord injury-induced neuropathic pain in male mice is associated with a persistent pro-inflammatory macrophage/microglial response in the superficial dorsal horn. Exp Neurol.

[R16] Cassetta L, Pollard JW (2023). A timeline of tumour-associated macrophage biology. Nat Rev Cancer.

[R17] Chang JW, Liu SC, Lin YY, He XY, Wu YS, Su CM, Tsai CH, Chen HT, Fong YC, Hu SL, Huang CC, Tang CH (2023). Nesfatin-1 stimulates CCL2-dependent monocyte migration and M1 macrophage polarization: implications for rheumatoid arthritis therapy. Int J Biol Sci.

[R18] Chang Q, Hao Y, Wang Y, Zhou Y, Zhuo H, Zhao G (2021). Bone marrow mesenchymal stem cell-derived exosomal microRNA-125a promotes M2 macrophage polarization in spinal cord injury by downregulating IRF5. Brain Res Bull.

[R19] Chen H, Wang Y, Tu W, Wang H, Yin H, Sha H, Li Y (2021). Effects of photobiomodulation combined with MSCs transplantation on the repair of spinal cord injury in rat. J Cell Physiol.

[R20] Chen J, He Q, Xie H, Gu B, Zhou L, Jiang D, Xie H, Liang L, Zhou Z, Zhang H (2025). Autophagy modulation by hADSCs and green light therapy alleviates inflammation and promotes functional recovery after spinal cord injury. Stem Cell Res Ther.

[R21] Chen R, Wang J, Dai X, Wu S, Huang Q, Jiang L, Kong X (2022). Augmented PFKFB3-mediated glycolysis by interferon-γ promotes inflammatory M1 polarization through the JAK2/STAT1 pathway in local vascular inflammation in Takayasu arteritis. Arthritis Res Ther.

[R22] Chen R, Zhang H, Tang B, Luo Y, Yang Y, Zhong X, Chen S, Xu X, Huang S, Liu C (2024). Macrophages in cardiovascular diseases: molecular mechanisms and therapeutic targets. Signal Transduct Target Ther.

[R23] Chen X, Dou J, Fu Z, Qiu Y, Zou L, Huang D, Tan X (2022). Macrophage M1 polarization mediated via the IL-6/STAT3 pathway contributes to apical periodontitis induced by Porphyromonas gingivalis. J Appl Oral Sci.

[R24] Colbert JD, Cruz FM, Rock KL (2020). Cross-presentation of exogenous antigens on MHC I molecules. Curr Opin Immunol.

[R25] Cui X, Huang C, Huang Y, Zhang Y, Wu J, Wang G, Zhou XZ, Zhang J, Wang L, Cheng L, Zhang KQ (2024). Amplification of metalloregulatory proteins in macrophages by bioactive ZnMn@SF hydrogels for spinal cord injury repair. ACS Nano.

[R26] David S, Zarruk JG, Ghasemlou N (2012). Inflammatory pathways in spinal cord injury. Int Rev Neurobiol.

[R27] David S, Greenhalgh AD, Kroner A (2015). Macrophage and microglial plasticity in the injured spinal cord. Neuroscience.

[R28] Di Carlo SE, Raffenne J, Varet H, Ode A, Granados DC, Stein M, Legendre R, Tuckermann J, Bousquet C, Peduto L (2023). Depletion of slow-cycling PDGFRα(+)ADAM12(+) mesenchymal cells promotes antitumor immunity by restricting macrophage efferocytosis. Nat Immunol.

[R29] Ding JY, Chen MJ, Wu LF, Shu GF, Fang SJ, Li ZY, Chu XR, Li XK, Wang ZG, Ji JS (2023). Mesenchymal stem cell-derived extracellular vesicles in skin wound healing: roles, opportunities and challenges. Mil Med Res.

[R30] Ding Y, Chen Q (2023). The NF-κB pathway: a focus on inflammatory responses in spinal cord injury. Mol Neurobiol.

[R31] Ding Y, Chen Q (2024). Recent advances on signaling pathways and their inhibitors in spinal cord injury. Biomed Pharmacother.

[R32] Dougherty KD, Dreyfus CF, Black IB (2000). Brain-derived neurotrophic factor in astrocytes, oligodendrocytes, and microglia/macrophages after spinal cord injury. Neurobiol Dis.

[R33] Du W, Deng Y, Jiang R, Tong L, Li R, Jiang X (2022). Clemastine enhances myelination, delays axonal loss and promotes functional recovery in spinal cord injury. Neurochem Res.

[R34] Edris NA, Aboulhoda BE, El-Din SS, Fouad AM, Albadawi E, Rashed LA, Elessawy KB (2023). Toll-like receptor signaling in the pathogenesis of chronic dacryocystitis: implication of c-FOS transcription factor and its downstream effector chemokine genes CCL2, CCL4, CXCL3, CXCR4 with a shift of the M1/M2 macrophage phenotype. Comb Chem High Throughput Screen.

[R35] Evans L, Milward K, Attanoos R, Clayton A, Errington R, Tabi Z (2021). Macrophage plasticity and function in the lung tumour microenvironment revealed in 3D heterotypic spheroid and explant models. Biomedicines.

[R36] Fan H, Tang HB, Chen Z, Wang HQ, Zhang L, Jiang Y, Li T, Yang CF, Wang XY, Li X, Wu SX, Zhang GL (2020). Inhibiting HMGB1-RAGE axis prevents pro-inflammatory macrophages/microglia polarization and affords neuroprotection after spinal cord injury. J Neuroinflammation.

[R37] Fan L, Dong J, He X, Zhang C, Zhang T (2021). Bone marrow mesenchymal stem cells-derived exosomes reduce apoptosis and inflammatory response during spinal cord injury by inhibiting the TLR4/MyD88/NF-κB signaling pathway. Hum Exp Toxicol.

[R38] Freire BM, de Melo FM, Basso AS (2022). Adrenergic signaling regulation of macrophage function: do we understand it yet?. Immunother Adv.

[R39] Freyermuth-Trujillo X, Segura-Uribe JJ, Salgado-Ceballos H, Orozco-Barrios CE, Coyoy-Salgado A (2022). Inflammation: A target for treatment in spinal cord injury. Cells.

[R40] Fu GQ, Wang YY, Xu YM, Bian MM, Zhang L, Yan HZ, Gao JX, Li JL, Chen YQ, Zhang N, Ding SQ, Wang R, Li JY, Hu JG, Lü HZ (2024). Exosomes derived from vMIP-II-Lamp2b gene-modified M2 cells provide neuroprotection by targeting the injured spinal cord, inhibiting chemokine signals and modulating microglia/macrophage polarization in mice. Exp Neurol.

[R41] Fu SP, Wu XC, Yang RL, Zhao DZ, Cheng J, Qian H, Ao J, Zhang Q, Zhang T (2023). The role and mechanisms of mesenchymal stem cells regulating macrophage plasticity in spinal cord injury. Biomed Pharmacother.

[R42] Funes SC, Rios M, Escobar-Vera J, Kalergis AM (2018). Implications of macrophage polarization in autoimmunity. Immunology.

[R43] Ge X (2021). Exosomal miR-155 from M1-polarized macrophages promotes EndoMT and impairs mitochondrial function via activating NF-κB signaling pathway in vascular endothelial cells after traumatic spinal cord injury. Redox Biol.

[R44] Geiß C, Salas E, Guevara-Coto J, Régnier-Vigouroux A, Mora-Rodríguez RA (2022). Multistability in macrophage activation pathways and metabolic implications. Cells.

[R45] Gensel JC, Zhang B (2015). Macrophage activation and its role in repair and pathology after spinal cord injury. Brain Res.

[R46] Gensel JC, Wang Y, Guan Z, Beckwith KA, Braun KJ, Wei P, McTigue DM, Popovich PG (2015). Toll-like receptors and dectin-1, a C-type lectin receptor, trigger divergent functions in CNS macrophages. J Neurosci.

[R47] Ghafouri-Fard S, Niazi V, Hussen BM, Omrani MD, Taheri M, Basiri A (2021). The emerging role of exosomes in the treatment of human disorders with a special focus on mesenchymal stem cells-derived exosomes. Front Cell Dev Biol.

[R48] Gong D, Wang Y, Zhong L, Jia M, Liu T, Li K (2021). Excretory dysfunction and quality of life after a spinal cord injury: A cross-sectional study. J Clin Nurs.

[R49] Green TRF, Rowe RK (2024). Quantifying microglial morphology: an insight into function. Clin Exp Immunol.

[R50] Gu G, Zhu B, Ren J, Song X, Fan B, Ding H, Shang J, Wu H, Li J, Wang H, Li J, Wei Z, Feng S (2023). Ang-(1-7)/MasR axis promotes functional recovery after spinal cord injury by regulating microglia/macrophage polarization. Cell Biosci.

[R51] Guo H, Li Z, Xiao B, Huang R (2024). M2 macrophage-derived exosomes promote angiogenesis and improve cardiac function after myocardial infarction. Biol Direct.

[R52] Guo J, Tang X, Deng P, Hui H, Chen B, An J, Zhang G, Shi K, Wang J, He Y, Hao D, Yang H (2024). Interleukin-4 from curcumin-activated OECs emerges as a central modulator for increasing M2 polarization of microglia/macrophage in OEC anti-inflammatory activity for functional repair of spinal cord injury. Cell Commun Signal.

[R53] Guo S, Dong L, Li J, Chen Y, Yao Y, Zeng R, Shushakova N, Haller H, Xu G, Rong S (2021). C-X3-C motif chemokine ligand 1/receptor 1 regulates the M1 polarization and chemotaxis of macrophages after hypoxia/reoxygenation injury. Chronic Dis Transl Med.

[R54] Guo X, Jiang C, Chen Z, Wang X, Hong F, Hao D (2023). Regulation of the JAK/STAT signaling pathway in spinal cord injury: an updated review. Front Immunol.

[R55] Hade MD, Suire CN, Suo Z (2021). Mesenchymal stem cell-derived exosomes: applications in regenerative medicine. Cells.

[R56] Han B, Liang W, Hai Y, Sun D, Ding H, Yang Y, Yin P (2024). Neurophysiological, histological, and behavioral characterization of animal models of distraction spinal cord injury: a systematic review. Neural Regen Res.

[R57] Han GH, Kim SJ, Ko WK, Lee D, Han IB, Sheen SH, Hong JB, Sohn S (2021). Transplantation of tauroursodeoxycholic acid-inducing M2-phenotype macrophages promotes an anti-neuroinflammatory effect and functional recovery after spinal cord injury in rats. Cell Prolif.

[R58] Hashimoto S, Nagoshi N, Shinozaki M, Nakanishi K, Suematsu Y, Shibata T, Kawai M, Kitagawa T, Ago K, Kamata Y, Yasutake K, Koya I, Ando Y, Minoda A, Shindo T, Shibata S, Matsumoto M, Nakamura M, Okano H (2023). Microenvironmental modulation in tandem with human stem cell transplantation enhances functional recovery after chronic complete spinal cord injury. Biomaterials.

[R59] Hassanshahi A, Hassanshahi M, Khabbazi S, Hosseini-Khah Z, Peymanfar Y, Ghalamkari S, Su YW, Xian CJ (2019). Adipose-derived stem cells for wound healing. J Cell Physiol.

[R60] He X, Li Y, Deng B, Lin A, Zhang G, Ma M, Wang Y, Yang Y, Kang X (2022). The PI3K/AKT signalling pathway in inflammation, cell death and glial scar formation after traumatic spinal cord injury: Mechanisms and therapeutic opportunities. Cell Prolif.

[R61] He Y, Gao Y, Zhang Q, Zhou G, Cao F, Yao S (2020). IL-4 switches microglia/macrophage M1/M2 polarization and alleviates neurological damage by modulating the JAK1/STAT6 pathway following ICH. Neuroscience.

[R62] He Y, Luo J, Xie H (2025). High glucose-induced alternative splicing of MEF2D in macrophages promotes vascular chronic inflammation in type 2 diabetes mellitus by mediating M1 macrophage polarization. Biochem Biophys Res Commun.

[R63] He Z, Pan X, Xie K, Sakao K, Chen J, Komatsu M, Hou DX (2025). The effects of fisetin on gene expression profile and cellular metabolism in ifn-γ-stimulated macrophage inflammation. Antioxidants (Basel).

[R64] Hellenbrand DJ, Quinn CM, Piper ZJ, Morehouse CN, Fixel JA, Hanna AS (2021). Inflammation after spinal cord injury: a review of the critical timeline of signaling cues and cellular infiltration. J Neuroinflammation.

[R65] Herb M, Schramm M (2021). Functions of ROS in macrophages and antimicrobial immunity. Antioxidants (Basel).

[R66] Hou Y, Zhang Y, Ma L, Luo D, Wang W, E S, Huang C, Hou Y, Chen S, Zhan J, Xu L, Lin D (2025). Tauroursodeoxycholic acid regulates macrophage/monocyte distribution and improves spinal microenvironment to promote nerve regeneration through inhibiting NF-κB signaling pathway in spinal cord injury. Front Pharmacol.

[R67] Howard EM, Strittmatter SM (2023). Development of neural repair therapy for chronic spinal cord trauma: soluble Nogo receptor decoy from discovery to clinical trial. Curr Opin Neurol.

[R68] Hsu CC, Wu KLH, Peng JM, Wu YN, Chen HT, Lee MS, Cheng JH (2024). Low-energy extracorporeal shockwave therapy improves locomotor functions, tissue regeneration, and modulating the inflammation induced FGF1 and FGF2 signaling to protect damaged tissue in spinal cord injury of rat model: an experimental animal study. Int J Surg.

[R69] Hsu CH, Pan YJ, Zheng YT, Lo RY, Yang FY (2023). Ultrasound reduces inflammation by modulating M1/M2 polarization of microglia through STAT1/STAT6/PPARγ signaling pathways. CNS Neurosci Ther.

[R70] Hu D, Dong R, Yang Y, Chen Z, Tang Y, Fu M, Wang DW, Xu X, Tu L (2019). Human kallikrein overexpression alleviates cardiac aging by alternatively regulating macrophage polarization in aged rats. FASEB J.

[R71] Hu D, Moalem-Taylor G, Potas JR (2020). Red-Light (670 nm) Therapy reduces mechanical sensitivity and neuronal cell death, and alters glial responses after spinal cord injury in rats. J Neurotrauma.

[R72] Hu K, Shang Z, Yang X, Zhang Y, Cao L (2023). Macrophage polarization and the regulation of bone immunity in bone homeostasis. J Inflamm Res.

[R73] Hu X, Xu W, Ren Y, Wang Z, He X, Huang R, Ma B, Zhao J, Zhu R, Cheng L (2023). Spinal cord injury: molecular mechanisms and therapeutic interventions. Signal Transduct Target Ther.

[R74] Hu X, Li Y, Liu X (2025). Sitagliptin phosphate ameliorates chronic inflammation in diabetes mellitus via modulating macrophage polarization. Front Endocrinol (Lausanne).

[R75] Hua T, Kong E, Zhang H, Lu J, Huang K, Ding R, Wang H, Li J, Han C, Yuan H (2024). PRMT6 deficiency or inhibition alleviates neuropathic pain by decreasing glycolysis and inflammation in microglia. Brain Behav Immun.

[R76] Huang LY, Sun X, Pan HX, Wang L, He CQ, Wei Q (2023). Cell transplantation therapies for spinal cord injury focusing on bone marrow mesenchymal stem cells: Advances and challenges. World J Stem Cells.

[R77] Huang Y, Wu L, Zhao Y, Guo J, Li R, Ma S, Ying Z (2024). Schwann cell promotes macrophage recruitment through IL-17B/IL-17RB pathway in injured peripheral nerves. Cell Rep.

[R78] Irgens I, Hoff JM, Jelnes R, Alexander M, Stanghelle JK, Thoresen M, Rekand T (2020). Spinal cord injury and development of pressure injury during acute rehabilitation in Norway: a national retrospective cross-sectional study. Spinal Cord.

[R79] Jang J, Hong A, Chung Y, Jin B (2022). Interleukin-4 aggravates LPS-induced striatal neurodegeneration in vivo via oxidative stress and polarization of microglia/macrophages. Int J Mol Sci.

[R80] Jazayeri SB, Maroufi SF, Mohammadi E, Dabbagh Ohadi MA, Hagen EM, Chalangari M, Jazayeri SB, Safdarian M, Zadegan SA, Ghodsi Z, Rahimi-Movaghar V (2023). Incidence of traumatic spinal cord injury worldwide: A systematic review, data integration, and update. World Neurosurg X.

[R81] Ji R, Hao Z, Wang H, Su Y, Yang W, Li X, Duan L, Guan F, Ma S (2024). Fisetin promotes functional recovery after spinal cord injury by inhibiting microglia/macrophage M1 polarization and JAK2/STAT3 signaling pathway. J Agric Food Chem.

[R82] Ji Z, Jiang X, Li Y, Song J, Chai C, Lu X (2020). Neural stem cells induce M2 polarization of macrophages through the upregulation of interleukin-4. Exp Ther Med.

[R83] Jin Y, Song Y, Lin J, Liu T, Li G, Lai B, Gu Y, Chen G, Xing L (2023). Role of inflammation in neurological damage and regeneration following spinal cord injury and its therapeutic implications. Burns Trauma.

[R84] Ju C, Ma YG, Zuo XS, Wang XK, Song ZW, Zhang ZH, Zhu ZJ, Li X, Liang ZW, Ding T, Wang Z, Hu XY (2023). Potential targets and mechanisms of photobiomodulation for spinal cord injury. Neural Regen Res.

[R85] Kazakova E, Iamshchikov P, Larionova I, Kzhyshkowska J (2022). Macrophage scavenger receptors: Tumor support and tumor inhibition. Front Oncol.

[R86] Khayati S, Dehnavi S, Sadeghi M, Tavakol Afshari J, Esmaeili SA, Mohammadi M (2023). The potential role of miRNA in regulating macrophage polarization. Heliyon.

[R87] Kiseleva V, Vishnyakova P, Elchaninov A, Fatkhudinov T, Sukhikh G (2023). Biochemical and molecular inducers and modulators of M2 macrophage polarization in clinical perspective. Int Immunopharmacol.

[R88] Kohno K, Tsuda M (2025). Neuron-microglia interactions modulating neuropathic pain. Int Immunol.

[R89] Kolliniati O, Ieronymaki E, Vergadi E, Tsatsanis C (2022). Metabolic regulation of macrophage activation. J Innate Immun.

[R90] Kopper TJ, Zhang B, Bailey WM, Bethel KE, Gensel JC (2021). The effects of myelin on macrophage activation are phenotypic specific via cPLA(2) in the context of spinal cord injury inflammation. Sci Rep.

[R91] Kotani N, Hashimoto H, Sessler DI, Yasuda T, Ebina T, Muraoka M, Matsuki A (1999). Expression of genes for proinflammatory cytokines in alveolar macrophages during propofol and isoflurane anesthesia. Anesth Analg.

[R92] Kovaleva O, Sorokin M, Egorova A, Petrenko A, Shelekhova K, Gratchev A (2023). Macrophage - tumor cell interaction beyond cytokines. Front Oncol.

[R93] Kroner A, Greenhalgh AD, Zarruk JG, Passos Dos Santos R, Gaestel M, David S (2014). TNF and increased intracellular iron alter macrophage polarization to a detrimental M1 phenotype in the injured spinal cord. Neuron.

[R94] Kumar S, Leigh ND, Cao X (2018). The role of co-stimulatory/co-inhibitory signals in graft-vs.-host disease. Front Immunol.

[R95] Kusnadi A, Park SH, Yuan R, Pannellini T, Giannopoulou E, Oliver D, Lu T, Park-Min KH, Ivashkiv LB (2019). The cytokine TNF promotes transcription factor SREBP activity and binding to inflammatory genes to activate macrophages and limit tissue repair. Immunity.

[R96] Lee JY, Park CS, Seo KJ, Kim IY, Han S, Youn I, Yune TY (2023). IL-6/JAK2/STAT3 axis mediates neuropathic pain by regulating astrocyte and microglia activation after spinal cord injury. Exp Neurol.

[R97] Leuti A, Fazio D, Fava M, Piccoli A, Oddi S, Maccarrone M (2020). Bioactive lipids, inflammation and chronic diseases. Adv Drug Deliv Rev.

[R98] Li C, Xu MM, Wang K, Adler AJ, Vella AT, Zhou B (2018). Macrophage polarization and meta-inflammation. Transl Res.

[R99] Li C, Wu Z, Zhou L, Shao J, Hu X, Xu W, Ren Y, Zhu X, Ge W, Zhang K, Liu J, Huang R, Yu J, Luo D, Yang X, Zhu W, Zhu R, Zheng C, Sun YE, Cheng L (2022). Temporal and spatial cellular and molecular pathological alterations with single-cell resolution in the adult spinal cord after injury. Signal Transduct Target Ther.

[R100] Li C, Luo Y, Li S (2024). The roles of neural stem cells in myelin regeneration and repair therapy after spinal cord injury. Stem Cell Res Ther.

[R101] Li D, Li X, Zhang J, Tang Z, Tian A (2023). The immunomodulatory effect of IL-4 accelerates bone substitute material-mediated osteogenesis in aged rats via NLRP3 inflammasome inhibition. Front Immunol.

[R102] Li J, Jing Y, Bai F, Wu Y, Wang L, Yan Y, Jia Y, Yu Y, Jia B, Ali F (2022). Induced pluripotent stem cells as natural biofactories for exosomes carrying miR-199b-5p in the treatment of spinal cord injury. Front Pharmacol.

[R103] Li J, Zheng M, Ouyang F, Ye J, Huang J, Zhao Y, Wang J, Shan F, Li Z, Yu S, Yao F, Tian D, Cheng L, Jing J (2024). Interleukin-3 modulates macrophage phagocytic activity and promotes spinal cord injury repair. CNS Neurosci Ther.

[R104] Li M, Hou Q, Zhong L, Zhao Y, Fu X (2021). Macrophage related chronic inflammation in non-healing wounds. Front Immunol.

[R105] Li M, Qi B, Li Q, Zheng T, Wang Y, Liu B, Guan Y, Bai Y, Jian F, Xu ZD, Xu Q, Chen Z (2024). Human induced pluripotent stem cell/embryonic stem cell-derived pyramidal neuronal precursors show safety and efficacy in a rat spinal cord injury model. Cell Mol Life Sci.

[R106] Li M, Zhang T, Li P, Luan Z, Liu J, Wang Y, Zhang Y, Liu Y, Wang Y (2025). IL-4-primed human umbilical cord mesenchymal stem cells-derived extracellular vesicles facilitate recovery in spinal cord injury via the miR-21-5p/PDCD4-mediated shifting of macrophage M1/M2 polarization. Life Sci.

[R107] Li P, Ma C, Li J, You S, Dang L, Wu J, Hao Z, Li J, Zhi Y, Chen L, Sun S (2022). Proteomic characterization of four subtypes of M2 macrophages derived from human THP-1 cells. J Zhejiang Univ Sci B.

[R108] Li S, Ke Z, Peng X, Fan P, Chao J, Wu P, Xiao P, Zhou Y (2022). Injectable and fast gelling hyaluronate hydrogels with rapid self-healing ability for spinal cord injury repair. Carbohydr Polym.

[R109] Li SY, Guo YL, Tian JW, Zhang HJ, Li RF, Gong P, Yu ZL (2023). Anti-tumor strategies by harnessing the phagocytosis of macrophages. Cancers (Basel).

[R110] Li Y, Hao J, Kong X, Yuan W, Shen Y, Hui Z, Lu X (2024). Rabeprazole mitigates obesity-induced chronic inflammation and insulin resistance associated with increased M2-type macrophage polarization. Biochim Biophys Acta Mol Basis Dis.

[R111] Li Z, Zheng M, Yu S, Yao F, Luo Y, Liu Y, Tian D, Cheng L, Jing J (2021). M2 macrophages promote PDGFRβ(+) pericytes migration after spinal cord injury in mice via PDGFB/PDGFRβ pathway. Front Pharmacol.

[R112] Li Z, Zhang L, Wang Y, Zhu Y, Shen H, Yuan J, Li X, Yu Z, Song B (2025). LA-peptide Hydrogel-Regulation of macrophage and fibroblast fates and their crosstalk via attenuating TGF-β to promote scarless wound healing. Bioact Mater.

[R113] Liang Z, Yang Z, Xie H, Rao J, Xu X, Lin Y, Wang C, Chen C (2024). Small extracellular vesicles from hypoxia-preconditioned bone marrow mesenchymal stem cells attenuate spinal cord injury via miR-146a-5p-mediated regulation of macrophage polarization. Neural Regen Res.

[R114] Lin W, Shen P, Huang Y, Han L, Ba X, Huang Y, Yan J, Li T, Xu L, Qin K, Chen Z, Tu S (2023). Wutou decoction attenuates the synovial inflammation of collagen-induced arthritis rats via regulating macrophage M1/M2 type polarization. J Ethnopharmacol.

[R115] Liu C, Hu F, Jiao G, Guo Y, Zhou P, Zhang Y, Zhang Z, Yi J, You Y, Li Z, Wang H, Zhang X (2022). Dental pulp stem cell-derived exosomes suppress M1 macrophage polarization through the ROS-MAPK-NFκB P65 signaling pathway after spinal cord injury. J Nanobiotechnology.

[R116] Liu D, Huang Y, Li B, Jia C, Liang F, Fu Q (2015). Carvedilol promotes neurological function, reduces bone loss and attenuates cell damage after acute spinal cord injury in rats. Clin Exp Pharmacol Physiol.

[R117] Liu F, Qiu H, Xue M, Zhang S, Zhang X, Xu J, Chen J, Yang Y, Xie J (2019). MSC-secreted TGF-β regulates lipopolysaccharide-stimulated macrophage M2-like polarization via the Akt/FoxO1 pathway. Stem Cell Res Ther.

[R118] Liu S, Huang B, Cao J, Wang Y, Xiao H, Zhu Y, Zhang H (2023). ROS fine-tunes the function and fate of immune cells. Int Immunopharmacol.

[R119] Liu X, Liu J, Zhao S, Zhang H, Cai W, Cai M, Ji X, Leak RK, Gao Y, Chen J, Hu X (2016). Interleukin-4 is essential for microglia/macrophage M2 polarization and long-term recovery after cerebral ischemia. Stroke.

[R120] Liu Z, Xiang C, Zhao X, Aizawa T, Niu R, Zhao J, Guo F, Li Y, Luo W, Liu W, Gu R (2024). Regulation of dynamic spatiotemporal inflammation by nanomaterials in spinal cord injury. J Nanobiotechnology.

[R121] Liu ZC, Yu WW, Zhou HC, Lan ZC, Wu T, Xiong SM, Yan L, Liu HB (2021). Lycium barbarum polysaccharides ameliorate LPS-induced inflammation of RAW264.7 cells and modify the behavioral score of peritonitis mice. J Food Biochem.

[R122] Lu C, Liu Y, Miao L, Kong X, Li H, Chen H, Zhao X, Zhang B, Cui X (2024). Research progress on the role of tumor-associated macrophages in tumor development and their use as molecular targets (Review). Int J Oncol.

[R123] Lu Y, Shang Z, Zhang W, Pang M, Hu X, Dai Y, Shen R, Wu Y, Liu C, Luo T, Wang X, Liu B, Zhang L, Rong L (2024). Global incidence and characteristics of spinal cord injury since 2000-2021: a systematic review and meta-analysis. BMC Med.

[R124] Lu ZJ, Pan QL, Lin FX (2024). Epigenetic modifications of inflammation in spinal cord injury. Biomed Pharmacother.

[R125] Luo F, Li H, Ma W, Cao J, Chen Q, Lu F, Qiu M, Zhou P, Xia Z, Zeng K, Zhan J, Zhou T, Luo Q, Pan W, Zhang L, Lin C, Huang Y, Zhang L, Yang D, Zhao H (2024). The BCL-2 inhibitor APG-2575 resets tumor-associated macrophages toward the M1 phenotype, promoting a favorable response to anti-PD-1 therapy via NLRP3 activation. Cell Mol Immunol.

[R126] Luo P, Du M, Sun Q, Zhao T, He H (2023). IL-38 suppresses macrophage M1 polarization to ameliorate synovial inflammation in the TMJ via GLUT-1 inhibition. Int Immunopharmacol.

[R127] Ma D, Fu C, Li F, Ruan R, Lin Y, Li X, Li M, Zhang J (2024). Functional biomaterials for modulating the dysfunctional pathological microenvironment of spinal cord injury. Bioact Mater.

[R128] Ma Z, Meng C, Wang X, Zhao Y, Wang J, Chen Y, Li Y, Jiang Y, Ouyang F, Li J, Zheng M, Cheng L, Jing J (2025). Trehalose enhances macrophage autophagy to promote myelin debris clearance after spinal cord injury. Cell Biosci.

[R129] Mahajan KR, Herman D, Zheng Y, Androjna C, Thoomukuntla B, Ontaneda D, Nakamura K, Trapp BD (2025). Neurodegeneration and demyelination in the multiple sclerosis spinal cord: clinical, pathological, and 7T MRI PERSPECTIVES. Neurology.

[R130] Maldonado-Lasunción I, Verhaagen J, Oudega M (2018). Mesenchymal stem cell-macrophage choreography supporting spinal cord repair. Neurotherapeutics.

[R131] Manoharan RR, Prasad A, Pospíšil P, Kzhyshkowska J (2024). ROS signaling in innate immunity via oxidative protein modifications. Front Immunol.

[R132] Marrocco A, Ortiz LA (2022). Role of metabolic reprogramming in pro-inflammatory cytokine secretion from LPS or silica-activated macrophages. Front Immunol.

[R133] Marrufo AM, Flores-Mireles AL (2024). Macrophage fate: to kill or not to kill?. Infect Immun.

[R134] Mata R, Yao Y, Cao W, Ding J, Zhou T, Zhai Z, Gao C (2021). The dynamic inflammatory tissue microenvironment: signality and disease therapy by biomaterials. Research (Wash D C).

[R135] Maynard G, Kannan R, Liu J, Wang W, Lam TKT, Wang X, Adamson C, Hackett C, Schwab JM, Liu C, Leslie DP, Chen D, Marino R, Zafonte R, Flanders A, Block G, Smith E, Strittmatter SM (2023). Soluble Nogo-receptor-Fc decoy (AXER-204) in patients with chronic cervical spinal cord injury in the USA: a first-in-human and randomised clinical trial. Lancet Neurol.

[R136] Mey GM, Mahajan KR, DeSilva TM (2023). Neurodegeneration in multiple sclerosis. WIREs Mech Dis.

[R137] Mhaidly N, Journe F, Najem A, Stock L, Trelcat A, Dequanter D, Saussez S, Descamps G (2023). Macrophage profiling in head and neck cancer to improve patient prognosis and assessment of cancer cell-macrophage interactions using three-dimensional coculture models. Int J Mol Sci.

[R138] Milich LM, Choi JS, Ryan C, Cerqueira SR, Benavides S, Yahn SL, Tsoulfas P, Lee JK (2021). Single-cell analysis of the cellular heterogeneity and interactions in the injured mouse spinal cord. J Exp Med.

[R139] Ming-Chin Lee K, Achuthan AA, De Souza DP, Lupancu TJ, Binger KJ, Lee MKS, Xu Y, McConville MJ, de Weerd NA, Dragoljevic D, Hertzog PJ, Murphy AJ, Hamilton JA, Fleetwood AJ (2022). Type I interferon antagonism of the JMJD3-IRF4 pathway modulates macrophage activation and polarization. Cell Rep.

[R140] Miranpuri GS, Bali P, Nguyen J, Kim JJ, Modgil S, Mehra P, Buttar S, Brown G, Yutuc N, Singh H, Wood A, Singh J, Anand A (2021). Role of microglia and astrocytes in spinal cord injury induced neuropathic pain. Ann Neurosci.

[R141] Moritz C (2024). Non-invasive spinal cord electrical stimulation for arm and hand function in chronic tetraplegia: a safety and efficacy trial. Nat Med.

[R142] Mosser DM, Hamidzadeh K, Goncalves R (2021). Macrophages and the maintenance of homeostasis. Cell Mol Immunol.

[R143] Muhammad W, Zhu J, Zhai Z, Xie J, Zhou J, Feng X, Feng B, Pan Q, Li S, Venkatesan R, Li P, Cao H, Gao C (2022). ROS-responsive polymer nanoparticles with enhanced loading of dexamethasone effectively modulate the lung injury microenvironment. Acta Biomater.

[R144] Mussbacher M, Derler M, Basílio J, Schmid JA (2023). NF-κB in monocytes and macrophages - an inflammatory master regulator in multitalented immune cells. Front Immunol.

[R145] Mussttaf RA, Jenkins DFL, Jha AN (2019). Assessing the impact of low level laser therapy (LLLT) on biological systems: a review. Int J Radiat Biol.

[R146] Myatich A, Haque A, Sole C, Banik NL (2023). Clemastine in remyelination and protection of neurons and skeletal muscle after spinal cord injury. Neural Regen Res.

[R147] Mylvaganam S, Freeman SA, Grinstein S (2021). The cytoskeleton in phagocytosis and macropinocytosis. Curr Biol.

[R148] Nakajima H, Honjoh K, Watanabe S, Kubota A, Matsumine A (2020). Distribution and polarization of microglia and macrophages at injured sites and the lumbar enlargement after spinal cord injury. Neurosci Lett.

[R149] Nakao Y, Fukuda T, Zhang Q, Sanui T, Shinjo T, Kou X, Chen C, Liu D, Watanabe Y, Hayashi C, Yamato H, Yotsumoto K, Tanaka U, Taketomi T, Uchiumi T, Le AD, Shi S, Nishimura F (2021). Exosomes from TNF-α-treated human gingiva-derived MSCs enhance M2 macrophage polarization and inhibit periodontal bone loss. Acta Biomater.

[R150] Ni Y, Zhuge F, Ni L, Nagata N, Yamashita T, Mukaida N, Kaneko S, Ota T, Nagashimada M (2022). CX3CL1/CX3CR1 interaction protects against lipotoxicity-induced nonalcoholic steatohepatitis by regulating macrophage migration and M1/M2 status. Metabolism.

[R151] Oronsky B, Caroen S, Reid T (2022). What exactly is inflammation (and what is it not?). Int J Mol Sci.

[R152] Orr MB, Gensel JC (2018). Spinal cord injury scarring and inflammation: therapies targeting glial and inflammatory responses. Neurotherapeutics.

[R153] Pan K, Li Q, Guo Z, Li Z (2025). Healing action of Interleukin-4 (IL-4) in acute and chronic inflammatory conditions: Mechanisms and therapeutic strategies. Pharmacol Ther.

[R154] Pan RY, He L, Zhang J, Liu X, Liao Y, Gao J, Liao Y, Yan Y, Li Q, Zhou X, Cheng J, Xing Q, Guan F, Zhang J, Sun L, Yuan Z (2022). Positive feedback regulation of microglial glucose metabolism by histone H4 lysine 12 lactylation in Alzheimer’s disease. Cell Metab.

[R155] Park SG, An JH, Li Q, Chae HK, Park SM, Lee JH, Ahn JO, Song WJ, Youn HY (2021). Feline adipose tissue-derived mesenchymal stem cells pretreated with IFN-γ enhance immunomodulatory effects through the PGE₂ pathway. J Vet Sci.

[R156] Patil V, Bohara R, Krishna Kanala V, McMahon S, Pandit A (2023). Models and approaches to comprehend and address glial inflammation following spinal cord injury. Drug Discov Today.

[R157] Patilas C, Varsamos I, Galanis A, Vavourakis M, Zachariou D, Marougklianis V, Kolovos I, Tsalimas G, Karampinas P, Kaspiris A, Vlamis J, Pneumaticos S (2024). The role of interleukin-10 in the pathogenesis and treatment of a spinal cord injury. Diagnostics (Basel).

[R158] Pavlov S, Babenko N, Kumetchko M, Litvinova O, Bereznyakov V, Litvinienko T (2025). The effect of photobiomodulation therapy on the expression of regulatory molecules in chronic wound healing. Pol Merkur Lekarski.

[R159] Peng P, Yu H, Xing C, Tao B, Li C, Huang J, Ning G, Zhang B, Feng S (2021). Exosomes-mediated phenotypic switch of macrophages in the immune microenvironment after spinal cord injury. Biomed Pharmacother.

[R160] Peng R, Zhang L, Xie Y, Guo S, Cao X, Yang M (2024). Spatial multi-omics analysis of the microenvironment in traumatic spinal cord injury: a narrative review. Front Immunol.

[R161] Peng W, Xie Y, Liu Y, Xu J, Yuan F, Li C, Qin T, Lu H, Duan C, Hu J (2023). Targeted delivery of CD163(+) macrophage-derived small extracellular vesicles via RGD peptides promote vascular regeneration and stabilization after spinal cord injury. J Control Release.

[R162] Perez-Gianmarco L, Kukley M (2023). Understanding the role of the glial scar through the depletion of glial cells after spinal cord injury. Cells.

[R163] Pomeshchik Y, Kidin I, Korhonen P, Savchenko E, Jaronen M, Lehtonen S, Wojciechowski S, Kanninen K, Koistinaho J, Malm T (2015). Interleukin-33 treatment reduces secondary injury and improves functional recovery after contusion spinal cord injury. Brain Behav Immun.

[R164] Poppell M, Hammel G, Ren Y (2023). Immune regulatory functions of macrophages and microglia in central nervous system diseases. Int J Mol Sci.

[R165] Presta I, Vismara M, Novellino F, Donato A, Zaffino P, Scali E, Pirrone KC, Spadea MF, Malara N, Donato G (2018). Innate immunity cells and the neurovascular unit. Int J Mol Sci.

[R166] Qiao G, Ji W, Sun Z, Wang X, Li P, Jia H, Duan L, Qi F (2022). Isosteviol reduces the acute inflammatory response after burns by upregulating MMP9 in macrophages leading to M2 polarization. Int Immunopharmacol.

[R167] Rahimi Darehbagh R, Seyedoshohadaei SA, Ramezani R, Rezaei N (2024). Stem cell therapies for neurological disorders: current progress, challenges, and future perspectives. Eur J Med Res.

[R168] Rai V, Dilisio MF, Samadi F, Agrawal DK (2022). Counteractive effects of IL-33 and IL-37 on inflammation in osteoarthritis. Int J Environ Res Public Health.

[R169] Ralston DJ, Elberg SS (1969). Serum-mediated immune cellular responses to Brucella melitensis. IV. Infection of macrophages under anaerobic conditions. J Bacteriol.

[R170] Ratajczak MZ, Thetchinamoorthy K, Wierzbicka D, Konopko A, Ratajczak J, Kucia M (2025). Extracellular microvesicles/exosomes-magic bullets in horizontal transfer between cells of mitochondria and molecules regulating mitochondria activity. Stem Cells.

[R171] Ren J, Zhu B, Gu G, Zhang W, Li J, Wang H, Wang M, Song X, Wei Z, Feng S (2023). Schwann cell-derived exosomes containing MFG-E8 modify macrophage/microglial polarization for attenuating inflammation via the SOCS3/STAT3 pathway after spinal cord injury. Cell Death Dis.

[R172] Reyes C, Mokalled MH (2024). Astrocyte-neuron interactions in spinal cord injury. Adv Neurobiol.

[R173] Richards JH, Freeman DD, Detloff MR (2024). Myeloid cell association with spinal cord injury-induced neuropathic pain and depressive-like behaviors in LysM-eGFP mice. J Pain.

[R174] Rumpel N, Riechert G, Schumann J (2024). miRNA-mediated fine regulation of TLR-induced M1 polarization. Cells.

[R175] Ryu JH, Park J, Kim BY, Kim Y, Kim NG, Shin YI (2023). Photobiomodulation ameliorates inflammatory parameters in fibroblast-like synoviocytes and experimental animal models of rheumatoid arthritis. Front Immunol.

[R176] Saad H, El Baba B, Tfaily A, Kobeissy F, Gonzalez JG, Refai D, Rodts GR, Mustroph C, Gimbel D, Grossberg J, Barrow DL, Gary MF, Alawieh AM (2025). Complement-dependent neuroinflammation in spinal cord injury: from pathology to therapeutic implications. Neural Regen Res.

[R177] Salvador AFM, Dykstra T, Rustenhoven J, Gao W, Blackburn SM, Bhasiin K, Dong MQ, Guimarães RM, Gonuguntla S, Smirnov I, Kipnis J, Herz J (2023). Age-dependent immune and lymphatic responses after spinal cord injury. Neuron.

[R178] Saraiva M, Vieira P, O’Garra A (2020). Biology and therapeutic potential of interleukin-10. J Exp Med.

[R179] Saremi J, Mahmoodi N, Rasouli M, Ranjbar FE, Mazaheri EL, Akbari M, Hasanzadeh E, Azami M (2022). Advanced approaches to regenerate spinal cord injury: The development of cell and tissue engineering therapy and combinational treatments. Biomed Pharmacother.

[R180] Scherlinger M, Richez C, Tsokos GC, Boilard E, Blanco P (2023). The role of platelets in immune-mediated inflammatory diseases. Nat Rev Immunol.

[R181] Schmidt J, Quintá HR (2023). Mitochondrial dysfunction as a target in spinal cord injury: intimate correlation between pathological processes and therapeutic approaches. Neural Regen Res.

[R182] Sekiya T, Holley MC (2025). The glial scar: to penetrate or not for motor pathway restoration?. Cell Transplant.

[R183] Seldenrijk CA, Drexhage HA, Meuwissen SG, Pals ST, Meijer CJ (1989). Dendritic cells and scavenger macrophages in chronic inflammatory bowel disease. Gut.

[R184] Shafqat A, Albalkhi I, Magableh HM, Saleh T, Alkattan K, Yaqinuddin A (2023). Tackling the glial scar in spinal cord regeneration: new discoveries and future directions. Front Cell Neurosci.

[R185] Shah R, Ibis B, Kashyap M, Boussiotis VA (2024). The role of ROS in tumor infiltrating immune cells and cancer immunotherapy. Metabolism.

[R186] Shahrezaei A, Sohani M, Sohouli M, Taherkhani S, Nasirinezhad F (2024). The involvement and significance of M2 macrophages in neuropathic pain following spinal cord injury: a systematic review. J Physiol Sci.

[R187] Shao Y, Lan Y, Chai X, Gao S, Zheng J, Huang R, Shi Y, Xiang Y, Guo H, Xi Y, Yang L, Yang T (2023). CXCL8 induces M2 macrophage polarization and inhibits CD8(+) T cell infiltration to generate an immunosuppressive microenvironment in colorectal cancer. FASEB J.

[R188] Shapouri-Moghaddam A, Mohammadian S, Vazini H, Taghadosi M, Esmaeili SA, Mardani F, Seifi B, Mohammadi A, Afshari JT, Sahebkar A (2018). Macrophage plasticity, polarization, and function in health and disease. J Cell Physiol.

[R189] Shen R, Lu Y, Cai C, Wang Z, Zhao J, Wu Y, Zhang Y, Yang Y (2024). Research progress and prospects of benefit-risk assessment methods for umbilical cord mesenchymal stem cell transplantation in the clinical treatment of spinal cord injury. Stem Cell Res Ther.

[R190] Shen T, Zhang W, Wang X, Ren X (2024). Application of”Spinal cord fusion” in spinal cord injury repair and its neurological mechanism. Heliyon.

[R191] Shipman H, Monsour M, Foley MM, Marbacher S, Croci DM, Bisson EF (2024). Interleukin-6 in spinal cord injury: could immunomodulation replace immunosuppression in the management of acute traumatic spinal cord injuries?. J Neurol Surg A Cent Eur Neurosurg.

[R192] Sikora JP, Karawani J, Sobczak J (2023). Neutrophils and the systemic inflammatory response syndrome (SIRS). Int J Mol Sci.

[R193] Slater PG, Domínguez-Romero ME, Villarreal M, Eisner V, Larraín J (2022). Mitochondrial function in spinal cord injury and regeneration. Cell Mol Life Sci.

[R194] Snider BA, Eren F, Reeves RK, Rupp R, Kirshblum SC (2023). The international standards for neurological classification of spinal cord injury: classification accuracy and challenges. Top Spinal Cord Inj Rehabil.

[R195] Song C, Zhang K, Luo C, Zhao X, Xu B (2024). Inhibiting the NF-κB/DRP1 axis affords neuroprotection after spinal cord injury via inhibiting polarization of pro-inflammatory microglia. Front Biosci (Landmark Ed).

[R196] Song LJ, Han QX, Ding ZB, Liu K, Zhang XX, Guo MF, Ma D, Wang Q, Xiao BG, Ma CG (2024). Icariin ameliorates the cuprizone-induced demyelination associated with antioxidation and anti-inflammation. Inflammopharmacology.

[R197] Song Q (2023). Type I interferon signaling facilitates resolution of acute liver injury by priming macrophage polarization. Cell Mol Immunol.

[R198] Sprenkle NT, Serezani CH, Pua HH (2023). MicroRNAs in macrophages: regulators of activation and function. J Immunol.

[R199] Statello L, Maugeri M, Garre E, Nawaz M, Wahlgren J, Papadimitriou A, Lundqvist C, Lindfors L, Collén A, Sunnerhagen P, Ragusa M, Purrello M, Di Pietro C, Tigue N, Valadi H (2018). Identification of RNA-binding proteins in exosomes capable of interacting with different types of RNA: RBP-facilitated transport of RNAs into exosomes. PLoS One.

[R200] Sterner RC, Sterner RM (2022). Immune response following traumatic spinal cord injury: Pathophysiology and therapies. Front Immunol.

[R201] Stevens AR, Hadis M, Alldrit H, Milward MR, Di Pietro V, Gendoo DMA, Belli A, Palin W, Davies DJ, Ahmed Z (2025). Evaluation of transcriptomic changes after photobiomodulation in spinal cord injury. Sci Rep.

[R202] Subbarayan R, Murugan Girija D, Raja STK, Krishnamoorthy A, Srinivasan D, Shrestha R, Srivastava N, Ranga Rao S (2024). Conditioned medium-enriched umbilical cord mesenchymal stem cells: a potential therapeutic strategy for spinal cord injury, unveiling transcriptomic and secretomic insights. Mol Biol Rep.

[R203] Sun C, Deng J, Ma Y, Meng F, Cui X, Li M, Li J, Li J, Yin P, Kong L, Zhang L, Tang P (2023). The dual role of microglia in neuropathic pain after spinal cord injury: Detrimental and protective effects. Exp Neurol.

[R204] Sun J, Liao Z, Li Z, Li H, Wu Z, Chen C, Wang H (2023). Down-regulation miR-146a-5p in Schwann cell-derived exosomes induced macrophage M1 polarization by impairing the inhibition on TRAF6/NF-κB pathway after peripheral nerve injury. Exp Neurol.

[R205] Sun X, Jones ZB, Chen XM, Zhou L, So KF, Ren Y (2016). Multiple organ dysfunction and systemic inflammation after spinal cord injury: a complex relationship. J Neuroinflammation.

[R206] Sundaram B, Pandian N, Kim HJ, Abdelaal HM, Mall R, Indari O, Sarkar R, Tweedell RE, Alonzo EQ, Klein J, Pruett-Miller SM, Vogel P, Kanneganti TD (2024). NLRC5 senses NAD(+) depletion, forming a PANoptosome and driving PANoptosis and inflammation. Cell.

[R207] Taefehshokr N, Yin C, Heit B (2021). Rab GTPases in the differential processing of phagocytosed pathogens versus efferocytosed apoptotic cells. Histol Histopathol.

[R208] Tamaru T, Kobayakawa K, Saiwai H, Konno D, Kijima K, Yoshizaki S, Hata K, Iura H, Ono G, Haruta Y, Kitade K, Iida KI, Kawaguchi KI, Matsumoto Y, Kubota K, Maeda T, Okada S, Nakashima Y (2023). Glial scar survives until the chronic phase by recruiting scar-forming astrocytes after spinal cord injury. Exp Neurol.

[R209] Tan Y, Lai T, Li Y, Tang Q, Zhang W, Liu Q, Wu S, Peng X, Sui X, Reggiori F, Jiang X, Chen Q, Wang C (2024). An oil-in-gel type of organohydrogel loaded with methylprednisolone for the treatment of secondary injuries following spinal cord traumas. J Control Release.

[R210] Tang H, Gu Y, Jiang L, Zheng G, Pan Z, Jiang X (2023). The role of immune cells and associated immunological factors in the immune response to spinal cord injury. Front Immunol.

[R211] Tang P, Zhang Y, Chen C, Ji X, Ju F, Liu X, Gan WB, He Z, Zhang S, Li W, Zhang L (2015). In vivo two-photon imaging of axonal dieback, blood flow, and calcium influx with methylprednisolone therapy after spinal cord injury. Sci Rep.

[R212] Tang Y, Xu Z, Tang J, Xu Y, Li Z, Wang W, Wu L, Xi K, Gu Y, Chen L (2023). Architecture-engineered electrospinning cascade regulates spinal microenvironment to promote nerve regeneration. Adv Healthc Mater.

[R213] Telegin GB, Chernov AS, Malyavina EV, Minakov AN, Kazakov VA, Rodionov MV, Belogurov AA, Spallone A (2023). CSF - injected contrast medium enhances post-traumatic spinal cord cysts. An experimental study in rats. Eur Rev Med Pharmacol Sci.

[R214] Tian S, Wang Y, Wan J, Yang M, Fu Z (2024). Co-stimulators CD40-CD40L, a potential immune-therapy target for atherosclerosis: A review. Medicine (Baltimore).

[R215] Tian T, Zhang S, Yang M (2023). Recent progress and challenges in the treatment of spinal cord injury. Protein Cell.

[R216] Tomé D, Dias MS, Correia J, Almeida RD (2023). Fibroblast growth factor signaling in axons: from development to disease. Cell Commun Signal.

[R217] Tran N, Mills EL (2024). Redox regulation of macrophages. Redox Biol.

[R218] Tuo L, Song H, Jiang D, Bai X, Song G (2021). Mesenchymal stem cells transfected with anti-miRNA-204-3p inhibit acute rejection after heart transplantation by targeting C-X-C motif chemokine receptor 4 (CXCR4) in vitro. J Thorac Dis.

[R219] Van Broeckhoven J, Sommer D, Dooley D, Hendrix S, Franssen A (2021). Macrophage phagocytosis after spinal cord injury: when friends become foes. Brain.

[R220] Walter RJ, Berlin RD, Pfeiffer JR, Oliver JM (1980). Polarization of endocytosis and receptor topography on cultured macrophages. J Cell Biol.

[R221] Wan Y, Lin Y, Tan X, Gong L, Lei F, Wang C, Sun X, Du X, Zhang Z, Jiang J, Liu Z, Wang J, Zhou X, Wang S, Zhou X, Jing P, Zhong Z (2024). Injectable hydrogel to deliver bone mesenchymal stem cells preloaded with azithromycin to promote spinal cord repair. ACS Nano.

[R222] Wang C, Liu S, Li C, Wang Z, Ming R, Huang L (2024). Monitoring the cascade of monocyte-derived macrophages to influenza virus infection in human alveolus chips. ACS Appl Mater Interfaces.

[R223] Wang J, Tian F, Cao L, Du R, Tong J, Ding X, Yuan Y, Wang C (2023). Macrophage polarization in spinal cord injury repair and the possible role of microRNAs: A review. Heliyon.

[R224] Wang J, Zhao X, Wang Q, Zheng X, Simayi D, Zhao J, Yang P, Mao Q, Xia H (2024). FAM76B regulates PI3K/Akt/NF-κB-mediated M1 macrophage polarization by influencing the stability of PIK3CD mRNA. Cell Mol Life Sci.

[R225] Wang J, Wu Q, Wang X, Liu H, Chen M, Xu L, Zhang Z, Li K, Li W, Zhong J (2024). Targeting macrophage phenotypes and metabolism as novel therapeutic approaches in atherosclerosis and related cardiovascular diseases. Curr Atheroscler Rep.

[R226] Wang LT, Liu KJ, Sytwu HK, Yen ML, Yen BL (2021). Advances in mesenchymal stem cell therapy for immune and inflammatory diseases: Use of cell-free products and human pluripotent stem cell-derived mesenchymal stem cells. Stem Cells Transl Med.

[R227] Wang LX, Zhang SX, Wu HJ, Rong XL, Guo J (2019). M2b macrophage polarization and its roles in diseases. J Leukoc Biol.

[R228] Wang MJ, Zhang HL, Chen F, Guo XJ, Liu QG, Hou J (2024). The double-edged effects of IL-6 in liver regeneration, aging, inflammation, and diseases. Exp Hematol Oncol.

[R229] Wang Q, Wang X, Shang Z, Zhao L (2024). Mechanism and prospects of mitochondrial transplantation for spinal cord injury treatment. Stem Cell Res Ther.

[R230] Wang R, Xu B (2021). TGF-β1-modified MSC-derived exosomal miR-135b attenuates cartilage injury via promoting M2 synovial macrophage polarization by targeting MAPK6. Cell Tissue Res.

[R231] Wang T, Xue Y, Zhang W, Zheng Z, Peng X, Zhou Y (2024). Collagen sponge scaffolds loaded with Trichostatin A pretreated BMSCs-derived exosomes regulate macrophage polarization to promote skin wound healing. Int J Biol Macromol.

[R232] Wang X, Zhang Z, Zhu Z, Liang Z, Zuo X, Ju C, Song Z, Li X, Hu X, Wang Z (2021). Photobiomodulation promotes repair following spinal cord injury by regulating the transformation of A1/A2 reactive astrocytes. Front Neurosci.

[R233] Wang X, Wu H, Fang C, Li Z (2024). Insights into innate immune cell evasion by Chlamydia trachomatis. Front Immunol.

[R234] Wang Y, Wang X, Zhang H, Han B, Ye Y, Zhang M, Wang Y, Xue J, Wang C (2021). Transforming growth factor-β1 promotes M1 alveolar macrophage polarization in acute lung injury by up-regulating DNMT1 to mediate the microRNA-124/PELI1/IRF5 axis. Front Cell Infect Microbiol.

[R235] Wang Y, Wang X, Zou Z, Hu Y, Li S, Wang Y (2023). Conditioned medium from bone marrow mesenchymal stem cells relieves spinal cord injury through suppression of Gal-3/NLRP3 and M1 microglia/macrophage polarization. Pathol Res Pract.

[R236] Wang Y, Kyauk RV, Shen YA, Xie L, Reichelt M, Lin H, Jiang Z, Ngu H, Shen K, Greene JJ, Sheng M, Yuen TJ (2023). TREM2-dependent microglial function is essential for remyelination and subsequent neuroprotection. Glia.

[R237] Wang Y, Lin Q, Zhang H, Wang S, Cui J, Hu Y, Liu J, Li M, Zhang K, Zhou F, Jing Y, Geng Z, Su J (2023). M2 macrophage-derived exosomes promote diabetic fracture healing by acting as an immunomodulator. Bioact Mater.

[R238] Wang Z, Li D, Wang Y, Yuan P, Zhang W, Zhang Y, He F, Yang J, Bi H, Duan H (2025). Hyaluronic acid methacryloyl hydrogel with sustained IL-10 release promotes macrophage M2 polarization and motor function after spinal cord injury. J Biomater Appl.

[R239] Watanabe Y, Fukuda T, Hayashi C, Nakao Y, Toyoda M, Kawakami K, Shinjo T, Iwashita M, Yamato H, Yotsumoto K, Taketomi T, Uchiumi T, Sanui T, Nishimura F (2022). Extracellular vesicles derived from GMSCs stimulated with TNF-α and IFN-α promote M2 macrophage polarization via enhanced CD73 and CD5L expression. Sci Rep.

[R240] Wei L, Yang X, Wang J, Wang Z, Wang Q, Ding Y, Yu A (2023). H3K18 lactylation of senescent microglia potentiates brain aging and Alzheimer’s disease through the NFκB signaling pathway. J Neuroinflammation.

[R241] Widerström-Noga E (2023). Neuropathic pain and spinal cord injury: management, phenotypes, and biomarkers. Drugs.

[R242] Wu C, Pan Y, Wang L, Liu M, Tu P, Chen S, Shi L, Yan D, Ma Y, Guo Y (2024). Inhibition of HDAC6 promotes microvascular endothelial cells to phagocytize myelin debris and reduces inflammatory response to accelerate the repair of spinal cord injury. CNS Neurosci Ther.

[R243] Wu J, Tang J, Zhang L, Wang W, Li Z, Zhou L, Jiang X, Huang Y, Guo Q, Wang W, Ding Z, Cai F, Xi K, Gu Y, Chen L (2025). Biomimetic “trojan horse” fibers modulate innate immunity cascades for nerve regeneration. ACS Nano.

[R244] Wu W, Jia S, Xu H, Gao Z, Wang Z, Lu B, Ai Y, Liu Y, Liu R, Yang T, Luo R, Hu C, Kong L, Huang D, Yan L, Yang Z, Zhu L, Hao D (2023). Supramolecular hydrogel microspheres of platelet-derived growth factor mimetic peptide promote recovery from spinal cord injury. ACS Nano.

[R245] Wu X, Lu W, Xu C, Jiang C, Zhuo Z, Wang R, Zhang D, Cui Y, Chang L, Zuo X, Wang Y, Mei H, Zhang W, Zhang M, Li C (2023). Macrophages phenotype regulated by IL-6 are associated with the prognosis of platinum-resistant serous ovarian cancer: integrated analysis of clinical trial and omics. J Immunol Res.

[R246] Wu Y, Tang Z, Zhang J, Wang Y, Liu S (2022). Restoration of spinal cord injury: From endogenous repairing process to cellular therapy. Front Cell Neurosci.

[R247] Xia T, Fu S, Yang R, Yang K, Lei W, Yang Y, Zhang Q, Zhao Y, Yu J, Yu L, Zhang T (2023). Advances in the study of macrophage polarization in inflammatory immune skin diseases. J Inflamm (Lond).

[R248] Xia Y, Yang R, Wang H, Hou Y, Li Y, Zhu J, Xu F, Fu C (2022). Biomaterials delivery strategies to repair spinal cord injury by modulating macrophage phenotypes. J Tissue Eng.

[R249] Xu W, Zhao X, Daha MR, van Kooten C (2013). Reversible differentiation of pro- and anti-inflammatory macrophages. Mol Immunol.

[R250] Xuan-Qing C, Xiang-Yu LV, Shi-Jia LIU (2021). Baitouweng decoction alleviates dextran sulfate sodium-induced ulcerative colitis by regulating intestinal microbiota and the IL-6/STAT3 signaling pathway. J Ethnopharmacol.

[R251] Xuan W, Qu Q, Zheng B, Xiong S, Fan GH (2015). The chemotaxis of M1 and M2 macrophages is regulated by different chemokines. J Leukoc Biol.

[R252] Yagura K, Ohtaki H, Tsumuraya T, Sato A, Miyamoto K, Kawada N, Suzuki K, Nakamura M, Kanzaki K, Dohi K, Izumizaki M, Hiraizumi Y, Honda K (2020). The enhancement of CCL2 and CCL5 by human bone marrow-derived mesenchymal stem/stromal cells might contribute to inflammatory suppression and axonal extension after spinal cord injury. PLoS One.

[R253] Yamanaka K, Nakamura K, Shibahara T, Takashima M, Takaki H, Hidaka M, Komori M, Yoshikawa Y, Wakisaka Y, Ago T, Kitazono T (2023). Deletion of Nox4 enhances remyelination following cuprizone-induced demyelination by increasing phagocytic capacity of microglia and macrophages in mice. Glia.

[R254] Yan L, Wang J, Cai X, Liou YC, Shen HM, Hao J, Huang C, Luo G, He W (2024). Macrophage plasticity: signaling pathways, tissue repair, and regeneration. MedComm (2020).

[R255] Yang CY, Chang PY, Chen JY, Wu BS, Yang AH, Lee OK (2021). Adipose-derived mesenchymal stem cells attenuate dialysis-induced peritoneal fibrosis by modulating macrophage polarization via interleukin-6. Stem Cell Res Ther.

[R256] Yang L, Gao X, Tian D, Yang W, Xue S, Cao Z, Sun T (2023). Resolvin D2 activates anti-inflammatory microglia via restoring autophagy flux and alleviate neuropathic pain following spinal cord injury in rats. Exp Neurol.

[R257] Yang L, Tao W, Xie C, Chen Q, Zhao Y, Zhang L, Xiao X, Wang S, Zheng X (2024). Interleukin-37 ameliorates periodontitis development by inhibiting NLRP3 inflammasome activation and modulating M1/M2 macrophage polarization. J Periodontal Res.

[R258] Yang R, Zhang Y, Kang J, Zhang C, Ning B (2024). Chondroitin sulfate proteoglycans revisited: its mechanism of generation and action for spinal cord injury. Aging Dis.

[R259] Yang S, Yu B, Zhang Q, Zhang Y, Fu L, Zhou B, Wu H, Li J, Gong S (2025). Amantadine modulates novel macrophage phenotypes to enhance neural repair following spinal cord injury. J Transl Med.

[R260] Yang S, Fan L, Yin L, Zhao Y, Li W, Zhao R, Jia X, Dong F, Zheng Z, Zhao D, Wang J (2025). Ginseng exosomes modulate M1/M2 polarisation by activating autophagy and target IKK/IкB/NF-кB to alleviate inflammatory bowel disease. J Nanobiotechnology.

[R261] Ye S, Theotokis P, Lee JY, Kim MJ, Nheu D, Ellen O, Bedford T, Ramanujam P, Wright DK, McDonald SJ, Alrehaili A, Bakhuraysah M, Kang JH, Siatskas C, Tremblay CS, Curtis DJ, Grigoriadis N, Monif M, Strittmatter SM, Petratos S (2023). Nogo receptor-Fc delivered by haematopoietic cells enhances neurorepair in a multiple sclerosis model. Brain Commun.

[R262] Yi S, Tao X, Wang Y, Cao Q, Zhou Z, Wang S (2022). Effects of propofol on macrophage activation and function in diseases. Front Pharmacol.

[R263] Yin J, Gong G, Wan W, Liu X (2022). Pyroptosis in spinal cord injury. Front Cell Neurosci.

[R264] Yolcu YU, Wahood W, Goyal A, Alvi MA, Reeves RK, Qu W, Gerberi DJ, Bydon M (2020). Pharmacologic prophylaxis for heterotopic ossification following spinal cord injury: A systematic review and meta-analysi. Clin Neurol Neurosurg.

[R265] Yu Y, Yue Z, Xu M, Zhang M, Shen X, Ma Z, Li J, Xie X (2022). Macrophages play a key role in tissue repair and regeneration. PeerJ.

[R266] Yu Y, Hao J, Wang L, Zheng X, Xie C, Liu H, Wu J, Qiao S, Shi J (2023). Astragaloside IV antagonizes the malignant progression of breast cancer induced by macrophage M2 polarization through the TGF-β-regulated Akt/Foxo1 pathway. Pathol Res Pract.

[R267] Yuan F, Peng W, Yang Y, Xu J, Liu Y, Xie Y, Huang T, Shi C, Ding Y, Li C, Qin T, Xie S, Zhu F, Lu H, Huang J, Hu J (2023). Endothelial progenitor cell-derived exosomes promote anti-inflammatory macrophages via SOCS3/JAK2/STAT3 axis and improve the outcome of spinal cord injury. J Neuroinflammation.

[R268] Zai LJ, Wrathall JR (2005). Cell proliferation and replacement following contusive spinal cord injury. Glia.

[R269] Zeng F, Chen A, Chen W, Cheng S, Lin S, Mei R, Mei X (2024). Knockout of TNF-α in microglia decreases ferroptosis and convert microglia phenotype after spinal cord injury. Heliyon.

[R270] Zhang B, Lin F, Dong J, Liu J, Ding Z, Xu J (2021). Peripheral macrophage-derived exosomes promote repair after spinal cord injury by inducing local anti-inflammatory type microglial polarization via increasing autophagy. Int J Biol Sci.

[R271] Zhang C, Zhao S, Huang Z, Xue A, Liu H, Dai S, Zheng Z, Li Y, Guo X, Gu J, Zhang F, Wang F, Wang Y, Zhou X, Zhang S, Zhang H, Shen J, Chen J, Yin G (2025). Macropinocytosis enhances foamy macrophage formation and cholesterol crystallization to activate NLRP3 inflammasome after spinal cord injury. Redox Biol.

[R272] Zhang HM, Chen XJ, Li SP, Zhang JM, Sun J, Zhou LX, Zhou GP, Cui B, Sun LY, Zhu ZJ (2022). ILC2s expanded by exogenous IL-33 regulate CD45+CD11b+F4/80high macrophage polarization to alleviate hepatic ischemia-reperfusion injury. Front Immunol.

[R273] Zhang M, Han X, Yan L, Fu Y, Kou H, Shang C, Wang J, Liu H, Jiang C, Wang J, Cheng T (2024). Inflammatory response in traumatic brain and spinal cord injury: The role of XCL1-XCR1 axis and T cells. CNS Neurosci Ther.

[R274] Zhang MZ, Yao B, Yang S, Jiang L, Wang S, Fan X, Yin H, Wong K, Miyazawa T, Chen J, Chang I, Singh A, Harris RC (2012). CSF-1 signaling mediates recovery from acute kidney injury. J Clin Invest.

[R275] Zhang Q, Yu B, Zhang Y, Tian Y, Yang S, Chen Y, Wu H (2023). Combination of single-cell and bulk RNA seq reveals the immune infiltration landscape and targeted therapeutic drugs in spinal cord injury. Front Immunol.

[R276] Zhang Q, Zhang S, Chen H, Chen G, Cui C, Zhang J, Wang W, Zhang Q, Guo S (2023). Targeting of MALT1 may improve functional recovery and attenuate microglia M1 polarization-mediated neuroinflammation during spinal cord injury. Mol Neurobiol.

[R277] Zhang Q, Zheng J, Li L, Yeh JM, Xie X, Zhao Y, Li C, Hou G, Yan H (2025). Bioinspired conductive oriented nanofiber felt with efficient ROS clearance and anti-inflammation for inducing M2 macrophage polarization and accelerating spinal cord injury repair. Bioact Mater.

[R278] Zhang Z, Li M, Wang Y, Wu J, Li J (2014). Higenamine promotes M2 macrophage activation and reduces Hmgb1 production through HO-1 induction in a murine model of spinal cord injury. Int Immunopharmacol.

[R279] Zhang Z, Yue P, Lu T, Wang Y, Wei Y, Wei X (2021). Role of lysosomes in physiological activities, diseases, and therapy. J Hematol Oncol.

[R280] Zhang Z, Zhou H, Gu W, Wei Y, Mou S, Wang Y, Zhang J, Zhong Q (2024). CGI1746 targets σ(1)R to modulate ferroptosis through mitochondria-associated membranes. Nat Chem Biol.

[R281] Zhang Z, Wang P, Xiong Q, Xu S, Kang D, He Z, Yao C, Jian G (2024). Advancements in the study of IL-6 and its receptors in the pathogenesis of gout. Cytokine.

[R282] Zhang ZH, Wu TY, Ju C, Zuo XS, Wang XK, Ma YG, Luo L, Zhu ZJ, Song ZW, Yao Z, Zhou J, Wang Z, Hu XY (2024). Photobiomodulation increases M2-Type polarization of macrophages by inhibiting versican production after spinal cord injury. Mol Neurobiol.

[R283] Zhao B, Sun L, Yuan Q, Hao Z, An F, Zhang W, Zhu X, Wang B (2023). BAP31 knockout in macrophages affects CD4(+)T cell activation through upregulation of MHC class II molecule. Int J Mol Sci.

[R284] Zhao G, Miao H, Li X, Chen S, Hu Y, Wang Z, Hou Y (2016). TGF-β3-induced miR-494 inhibits macrophage polarization via suppressing PGE2 secretion in mesenchymal stem cells. FEBS Lett.

[R285] Zheng G, Yu W, Xu Z, Yang C, Wang Y, Yue Z, Xiao Q, Zhang W, Wu X, Zang F, Wang J, Wang L, Yuan WE, Hu B, Chen H (2024). Neuroimmune modulating and energy supporting nanozyme-mimic scaffold synergistically promotes axon regeneration after spinal cord injury. J Nanobiotechnology.

[R286] Zheng Q, Zhang J, Zuo X, Sun J, Liang Z, Hu X, Wang Z, Li K, Song J, Ding T, Shen X, Ma Y, Li P (2021). Photobiomodulation promotes neuronal axon regeneration after oxidative stress and induces a change in polarization from M1 to M2 in macrophages via stimulation of CCL2 in neurons: relevance to spinal cord injury. J Mol Neurosci.

[R287] Zhong WJ, Liu T, Yang HH, Duan JX, Yang JT, Guan XX, Xiong JB, Zhang YF, Zhang CY, Zhou Y, Guan CX (2023). TREM-1 governs NLRP3 inflammasome activation of macrophages by firing up glycolysis in acute lung injury. Int J Biol Sci.

[R288] Zhou HY, Wang X, Li Y, Wang D, Zhou XZ, Xiao N, Li GX, Li G (2025). Dynamic development of microglia and macrophages after spinal cord injury. Neural Regen Res.

[R289] Zhou P, Li Q, Su S, Dong W, Zong S, Ma Q, Yang X, Zuo D, Zheng S, Meng X, Xu D, Zeng Q (2020). Interleukin 37 suppresses M1 macrophage polarization through inhibition of the Notch1 and nuclear factor kappa B pathways. Front Cell Dev Biol.

[R290] Zhou R, Li J, Wang R, Chen Z, Zhou F (2023). The neurovascular unit in healthy and injured spinal cord. J Cereb Blood Flow Metab.

[R291] Zhou X, Wahane S, Friedl MS, Kluge M, Friedel CC, Avrampou K, Zachariou V, Guo L, Zhang B, He X, Friedel RH, Zou H (2020). Microglia and macrophages promote corralling, wound compaction and recovery after spinal cord injury via Plexin-B2. Nat Neurosci.

[R292] Zhu B, Gu G, Ren J, Song X, Li J, Wang C, Zhang W, Huo Y, Wang H, Jin L, Feng S, Wei Z (2023). Schwann cell-derived exosomes and methylprednisolone composite patch for spinal cord injury repair. ACS Nano.

[R293] Zhu D, Peng T, Zhang Z, Guo S, Su Y, Zhang K, Wang J, Liu C (2024). Mesenchymal stem cells overexpressing XIST induce macrophage M2 polarization and improve neural stem cell homeostatic microenvironment, alleviating spinal cord injury. J Tissue Eng.

[R294] Zhu J, Huang F, Hu Y, Qiao W, Guan Y, Zhang ZJ, Liu S, Liu Y (2023). Non-coding RNAs regulate spinal cord injury-related neuropathic pain via neuroinflammation. J Inflamm Res.

[R295] Zhu X, Xu Y, Wang J, Xue Z, Qiu T, Chen J (2023). Loss of NLRP3 reduces oxidative stress and polarizes intratumor macrophages to attenuate immune attack on endometrial cancer. Front Immunol.

